# Genome-Wide Association Studies in Diverse Spring Wheat Panel for Stripe, Stem, and Leaf Rust Resistance

**DOI:** 10.3389/fpls.2020.00748

**Published:** 2020-06-03

**Authors:** Deepender Kumar, Animesh Kumar, Vinod Chhokar, Om Prakash Gangwar, Subhash Chander Bhardwaj, M. Sivasamy, S. V. Sai Prasad, T. L. Prakasha, Hanif Khan, Rajender Singh, Pradeep Sharma, Sonia Sheoran, Mir Asif Iquebal, Sarika Jaiswal, Ulavappa B. Angadi, Gyanendra Singh, Anil Rai, Gyanendra Pratap Singh, Dinesh Kumar, Ratan Tiwari

**Affiliations:** ^1^Department of Bio and Nanotechnology, Guru Jambheshwar University of Science and Technology, Hisar, India; ^2^ICAR-Indian Institute of Wheat and Barley Research, Karnal, India; ^3^ICAR-Indian Agricultural Statistics Research Institute, New Delhi, India; ^4^ICAR-Indian Institute of Wheat and Barley Research, Regional Station, Shimla, India; ^5^ICAR-Indian Agricultural Research Institute, Regional Station, Wellington, India; ^6^ICAR-Indian Agricultural Research Institute, Regional Station, Indore, India

**Keywords:** 35K SNP array, GWAS, leaf rust, resistance, stripe rust, stem rust, wheat

## Abstract

Among several important wheat foliar diseases, Stripe rust (YR), Leaf rust (LR), and Stem rust (SR) have always been an issue of concern to the farmers and wheat breeders. Evolution of virulent pathotypes of these rusts has posed frequent threats to an epidemic. Pyramiding rust-resistant genes are the most economical and environment-friendly approach in postponing this inevitable threat. To achieve durable long term resistance against the three rusts, an attempt in this study was made searching for novel sources of resistant alleles in a panel of 483 spring wheat genotypes. This is a unique and comprehensive study where evaluation of a diverse panel comprising wheat germplasm from various categories and adapted to different wheat agro-climatic zones was challenged with 18 pathotypes of the three rusts with simultaneous screening in field conditions. The panel was genotyped using 35K SNP array and evaluated for each rust at two locations for two consecutive crop seasons. High heritability estimates of disease response were observed between environments for each rust type. A significant effect of population structure in the panel was visible in the disease response. Using a compressed mixed linear model approach, 25 genomic regions were found associated with resistance for at least two rusts. Out of these, seven were associated with all the three rusts on chromosome groups 1 and 6 along with 2B. For resistance against YR, LR, and SR, there were 16, 18, and 27 QTL (quantitative trait loci) identified respectively, associated at least in two out of four environments. Several of these regions got annotated with resistance associated genes *viz.* NB-LRR, E3-ubiquitin protein ligase, ABC transporter protein, etc. Alien introgressed (on 1B and 3D) and pleiotropic (on 7D) resistance genes were captured in seedling and adult plant disease responses, respectively. The present study demonstrates the use of genome-wide association for identification of a large number of favorable alleles for leaf, stripe, and stem rust resistance for broadening the genetic base. Quick conversion of these QTL into user-friendly markers will accelerate the deployment of these resistance loci in wheat breeding programs.

## Introduction

Among the many foliar diseases of wheat, rusts are the economically most significant fungal diseases threatening the food security of the world’s growing population. There are three types of rusts in wheat, stripe, or yellow rust (YR) caused by the fungus *Puccinia stritiformis* Westend. f.sp. *tritici* Eriks. (*Pst*), leaf, or brown rust (LR) caused by *Puccinia triticina* Eriks. (*Pt*), and stem or black rust (SR) caused by *Puccinia graminis* Pers. f. sp. *tritici* Eriks. & Henn. (*Pgt*). More than sixty wheat-producing countries distributed in all continents other than Antarctica have encountered the rusts proving their widespread presence. The damage caused by YR can be as high as 70% in case cultivars are susceptible and the climatic conditions are favorable for an early infection (Chen, 2005). LR has a widespread geographical presence causing considerable yield losses (Marasas et al., 2002). At an early onset, it causes much more damage to crop resulting in yield losses as compared to stem and stripe rusts (Bolton et al., 2008; Huerta-Espino et al., 2011). SR inflicts losses up to USD 1.12 billion worldwide which results particularly due to a reduction in yield and hampered end-use quality of the crop (Pardey et al., 2013). All three rusts pose a major threat to Indian farmers as well. North Indian conditions support the survival and spread of the YR fungal spores. LR is more prevalent in the whole of India as the disease is favored by intermediate temperatures. Also, LR is the most widely distributed amongst rusts and commonly visible in all wheat growing areas during the season. SR is contained mostly in the southern states of the country and survives throughout the year in the Nilgiri hills of southern India (Joshi et al., 1985; Nagarajan and Joshi, 1985).

In disease management practices, planting resistant varieties is the most economical, efficient, and ecologically acceptable tool to manage wheat rusts worldwide (McIntosh et al., 1995; Wiesner-Hanks and Nelson, 2016). Wheat cultivars with diverse resistance are deployed in different areas keeping in mind the pathotype distribution of three *Puccinia* species on wheat. To date, the reported number of officially designated resistance genes are more than seventy against both YR (78) and LR (77) in addition to about sixty designated resistance genes against SR (McIntosh et al., 2017). Two recently identified *Yr* genes *Yr*79 (Feng et al., 2018) and *Yr80* (Nsabiyera et al., 2018) have been added to the rust-resistant gene library. Resistance to multiple rusts can be broadly categorized as all stage or seedling resistance (ASR) and adult-plant resistance (APR). Of these reported genes, many genes provide resistance at the seedling stage of plants (McIntosh et al., 2017). Since seedling genes are pathogen race-specific, the cell death phenomenon is activated due to plant hypersensitive response preventing pathogen spread (Ellis et al., 2014; Mondal et al., 2016). This also puts intense selection pressure on the pathogen for its survival. In such cases, pathogen evades and evolves itself which in result renders the deployed all stage resistance gene to be ineffective in a very short time (Line and Qayoum, 1992; Burdon et al., 2014; Li et al., 2014; Niks et al., 2015). The rust resistant wheat with ASR favors the selection of new pathotypes that multiply without any competition on the resistant host resulting in susceptibility of the cultivar/gene. Several all stage resistant *Yr* genes have become susceptible for instance *Yr2*, *Yr6* to *Yr9*, *Yr17*, and *Yr 27* (Singh et al., 2008). Other genes such as *Lr1*, *Lr13, Lr24, Lr26, Lr37*; *Sr6, Sr8a*, and *Sr11* against LR and SR also succumbed (Kolmer et al., 2007; McIntosh et al., 2009, 2014; Ellis et al., 2014). A well-known outbreak causing great loss of yield was witnessed in the year 1998 due to the new *Pgt* virulent race Ug99. This resulted in the failure of ASR *Sr* genes *Sr24*, *Sr31*, *Sr36*, and *SrTmp* in subsequent years with the emergence of its new variants (Pretorius et al., 2000, 2010; Jin et al., 2007, 2008, 2009; Visser et al., 2011; Newcomb et al., 2016). On the other hand, APR resistance is usually governed by multiple genes and quantitatively gets less influenced by race-specific pathogens. The involved genes provide non-race-specific partial resistance to all the pathotypes of a given pathogen species, thus making it more durable (Lagudah, 2011; Burdon et al., 2014). Despite the fact that incorporating APR into new cultivars can be difficult when compared to ASR, it was found that many wheat cultivars possessing APR showed durable resistance (Mcintosh, 1992; Boyd, 2005; Navabi et al., 2005; Singh et al., 2005; Ren et al., 2012b; Chen, 2013; Randhawa et al., 2018). Some APR genes when used in combinations have been known to possess durable pleiotropic resistance against multiple wheat rusts and powdery mildew, *i.e., Lr34/Yr18/Sr57/Pm38* (on chromosome 7DS), *Lr46/Yr29/Sr58/Pm39* (on chromosome 1BL), and *Lr67/Yr46/Sr55/Pm46* (on chromosome 4DL) (Lagudah, 2011; Risk et al., 2012; Ellis et al., 2014), of which *Lr34* has been studied extensively in different crops including rice, barley, maize, and sorghum (Krattinger et al., 2013, 2019). Deployment of seedling (all-stage resistance), adult plant (non-race specific and race-specific) and slow rusting resistance is to create diversity for resistance (Bhardwaj et al., 2019b). The wheat lines possessing rust resistance at the seedling stage will remain resistant during the whole life of a wheat variety. Therefore, both types of resistance can be amalgamated to strengthen more extended and durable resistance in the future.

As most of the ASR genes go ineffective in the long run and APR genes have only minor effects, more resistance associated genomic regions are needed to be identified and utilized in wheat genetic improvement for rust resistance. Not all of the QTL identified so far were explored for use in marker-assisted selection (MAS). Therefore, finding user-friendly markers becomes even more necessary (Zeng et al., 2019). With the advent of NGS technologies, it became possible to develop high-throughput Single Nucleotide Polymorphism (SNP) markers which are abundant, co-dominant, and present throughout the wheat genome (Allen et al., 2013; Rasheed et al., 2016; Wu et al., 2017, 2018). These markers have advantages over traditional PCR based markers (Chen et al., 1998; Röder et al., 1998; Ren et al., 2012b; Rosewarne et al., 2013; Zhou et al., 2014) for being high-throughput, low cost for genotyping, high efficiency, and allele specificity (Gupta et al., 2008; Colasuonno et al., 2014; Semagn et al., 2014; Long et al., 2017). Genotyping by sequencing (GBS) and array-based SNP genotyping platforms are utilized globally. Several SNP arrays are now available in the market *viz*. 9K (Cavanagh et al., 2013), 15K (Boeven et al., 2016), 35K (Allen et al., 2017), 50K (Bayer et al., 2017), 90K (Wang et al., 2014), 55K, 660K (Jia and Zhao, 2016), and 820K (Winfield et al., 2016). Genome-wide association studies (GWAS) using these chips have certain advantages over bi-parental QTL mapping. Genetic architecture of complex traits in diverse germplasm collections can be studied using GWAS, which detects the genomic regions present in linkage disequilibrium (LD) with genes associated with the trait under study (Hall et al., 2010; Zhao et al., 2011; Riedelsheimer et al., 2012). Due to the accumulation of historical chromosomal recombinations over several generations in a natural population, QTL can be identified using GWAS at a higher mapping resolution (Yu and Buckler, 2006; Semagn et al., 2010). GWAS has been successfully used to study various traits in wheat such as grain yield (Sukumaran et al., 2018), eyespot disease resistance (Zanke et al., 2017), pre-harvest sprouting resistance (Zhou et al., 2017), 36 agro-morphological traits (Sheoran et al., 2019) and so on.

The current study was undertaken with the objective of performing a large-scale association study for identifying genomic regions responsible for resistance to the three rusts from a very diverse set of germplasm comprising of improved genotypes, released varieties, exotic collection, genetic stocks, landraces, and some mutant lines. The study was focused on both seedling and adult plant stage rust response under controlled and natural field conditions, respectively. To identify seedling resistance, the most virulent and predominantly prevalent pathotypes were used to challenge the wheat material in this study. Care was taken to select pathotypes with varied avirulence and virulence structure which would knock down most of the genes in present-day wheat material (Bhardwaj, 2011). Keeping the aforesaid points into consideration seven pathotypes of *Pgt* (Prasad et al., 2018), five pathotypes of *Pst* (Gangwar et al., 2019), and six pathotypes of *Pt* (Bhardwaj et al., 2019a) were selected for screening wheat material at seedling stage under controlled conditions. The optimum conditions ensure foolproof evaluation and selection of rust resistant material. The SNP marker enabled the identification of genomic regions associated with disease resistance will be further firmed up so as to harness less exploited resistant genes for broadening the resistance base thereby avoiding any substantial losses due to rust diseases.

## Materials and Methods

### Plant Materials and Genotyping

The diverse panel of 483 genotypes mentioned in our previous report (Kumar et al., 2020) was used for this study. The germplasm comprised of the exotic collection (34), genetic stocks (44), improved genotypes (120), landraces (96), mutant lines (2), and varieties (187). Genotypes in the panel are adapted to different agro-climatic zones of India. Henceforth, this diverse panel will be recalled as rust association mapping panel (RAMP). Genotyping of the panel was done using 35K Axiom^®^ Wheat Breeder’s Array (Allen et al., 2017, Affymetrix UK Ltd., United Kingdom) as per manufacturer’s guidelines with 35,143 SNP markers. The genotyping data was filtered by retaining markers with minor allele frequency (MAF) > 5%, genotypes with <10% missing SNP calls, and markers with <10% missingness. The obtained markers were further ordered according to the genetic map available at CerealDb (Wilkinson et al., 2012; Allen et al., 2017). This resulted in a selection of 14,650 polymorphic SNP markers as described previously (Kumar et al., 2020) for subsequent genetic analyses.

### Phenotypic Evaluation for Stripe Rust, Stem Rust, and Leaf Rust

The RAMP was evaluated for stripe rust (YR), stem rust (SR), and leaf rust (LR) both at the seedling stage in controlled conditions and at the adult plant stage in the field under natural disease pressure.

The seedling resistance test (SRT) was performed on this panel against five, six, and seven rust pathotypes/races for *Pst, Pt*, and *Pgt* respectively. The selection of pathotypes was based on the virulence and their prominence. They are namely YR_110S84, YR_110S119, YR_T, YR_238S119, YR_46S119 for *Pst*, LR_77-1, LR_77-5, LR_77-9, LR_12-5, LR_104, LR_106-2 for *Pt* and SR_40A, SR_21A2, SR_11, SR_34-1, SR_40-3, SR_117-6, SR_122 for *Pgt*. SRT was done under glasshouse conditions at Regional Station, ICAR-Indian Institute of Wheat and Barley Research (IIWBR), Shimla, India. The avirulence/virulence formulae for the 18 pathotypes are provided in Supplementary Table S1. The experiments were performed as mentioned by Bhardwaj (2011). The disease response recorded as infection type (IT) using Stakman scale (Stakman et al., 1962) was converted to a modified linear 0-9 scale (Riaz et al., 2016) as follows: 0; = 0, ;- = 0.5, ; = 1, 1- = 1.5, 1 = 2, 1 + = 3, 2- = 4, 2 = 5, 2 + = 6, 3- = 6.5, 3 = 7, 3 + = 8 and 4 = 9, where average was considered in case of complex scores. Genotypes with an IT score of >0 to <3, >3 to <6, and >6 to <9 were considered as resistant, moderate, and susceptible, respectively (Table 1). Taking pedigree relationships into consideration, the postulation of resistance genes were made by comparing the IT response of the pathotypes on the test lines with controls of known resistance genes.

**TABLE 1:** Summary of yellow rust, leaf rust, and stem rust responses at the seedling and adult plant stages.

**(A) Disease response summary at the seedling stage**							
***P. stritiformis (Pst)***	**Number of Genotypes (Percentage)**						
**Races**	**YR_110S119**	**YR_T**	**YR_238S119**	**YR_46S119**	**YR_110S84**		

Resistant	17 (3.5)	131 (27.8)	101(21.5)	227 (48.1)	137 (28.3)		
Moderate	17 (3.5)	71 (15.2)	69 (14.7)	72 (15.2)	49 (10.2)		
***P. triticina (Pt)***							
**Races**	**LR_106**	**LR_77-1**	**LR_77-5**	**LR_77-9**	**LR_12-5**	**LR_104-2**	

Resistant	361 (75.7)	166 (36.8)	63 (13.0)	51 (11.1)	119 (25.5)	98 (20.3)	
Moderate	37 (7.7)	116 (25.7)	61 (12.6)	53 (11.6)	156 (33.5)	75 (15.5)	
Susceptible	79 (16.5)	168 (37.3)	359 (74.3)	354 (77.3)	190 (40.8)	310 (64.1)	
***P. graminis (Pgt)***							
**Races**	**SR_40A**	**SR_21A2**	**SR_11**	**SR_34-1**	**SR_40-3**	**SR_117-6**	**SR_122**

Resistant	76 (15.7)	248 (54.7)	162 (34.3)	357 (75.9)	246 (55.0)	255 (52.8)	273 (57.6)
Moderate	217 (45.0)	149 (32.8)	146 (31.0)	53 (11.3)	115 (25.7)	110 (22.8)	126 (26.6)
Susceptible	189 (39.2)	57 (12.5)	164 (34.7)	60 (12.7)	86 (19.1)	118 (24.4)	75 (15.8)
**(B) Disease response summary at the adult plant stage**							
**Stripe rust**							
**Environment**	**COI_YR_E1**	**COI_YR_E2**	**COI_YR_E3**	**COI_YR_E4**			

Resistant	183 (37.9)	217 (45.0)	142 (29.4)	218 (45.1)			
Moderate	148 (30.7)	158 (32.7)	156 (32.3)	168 (34.8)			
Susceptible	152 (31.4)	108 (22.3)	184 (38.2)	96 (19.9)			
**Leaf rust**							
**Environment**	**COI_LR_E1**	**COI_LR_E2**	**COI_LR_E3**	**COI_LR_E4**			

Resistant	367 (76.0)	343 (74.0)	373 (78.6)	382 (79.1)			
Moderate	56 (11.5)	75 (16.0)	59 (12.4)	71 (14.6)			
Susceptible	6 (12.5)	46 (10.0)	43 (9.1)	30 (6.2)			
**Stem rust**							
**Environment**	**COI_SR_E1**	**COI_SR_E2**	**COI_SR_E3**	**COI_SR_E4**			

Resistant	299 (62.3)	316 (65.6)	233 (49.0)	300 (62.3)			
Moderate	129 (26.8)	137 (28.4)	177 (37.2)	152 (31.5)			
Susceptible	52 (10.8)	28 (5.8)	65 (13.6)	30 (6.2)			

Field evaluation of YR was done at two locations for two consecutive years *viz.* 2017-18 and 2018-19. By counting each location and year as one environment, a total of four environments (E1-E4) were considered. The plants were sown at seed farm, Uchani (29°42′48.7^″^N, 76°59′51.3^″^E), Haryana, India and ICAR-IIWBR, Karnal (29°42′10.0^″^N, 76°59′29.7^″^E), Haryana, India. These locations come under stripe rust-prone areas in the Northern region of India suitable to study natural disease pressure. Similarly, field evaluation for SR and LR was done at two locations for two years. The locations were ICAR-Indian Agricultural Research Institute (IARI), Regional Station, Wellington (11°22′47.5^″^N, 76°46′26.1^″^E), Tamil Nadu, India and ICAR-IARI, Regional Station, Indore (22°42′31.3^″^N, 75°53′29.2^″^E), Madhya Pradesh, India. These locations are prone to natural disease pressure of stem and leaf rusts. A total of 12 environments (4 for each rust) were considered for this study (Supplementary Table S2). The seeds were planted in a non-replicated augmented block design with single row of 1 m and the distance between two rows was 0.3 m. The planting was done in the first fortnight of November at Indore and Wellington and in the second fortnight at Karnal and Uchani, each year. A mixture of check lines susceptible to multiple rusts was planted as infector rows (at every 20th single row) and in spreader rows (perpendicular to the 1 m rows) surrounding the plot for establishing sufficient inoculum and uniform disease development. To ensure uniform disease distribution, spores were collected from the early infections that appeared naturally in the spreader rows and were used to inoculate the infector rows. The response to rust was recorded using disease severity (DS) and infection response (IR) as the two measures. DS was measured using the modified Cobb scale (Peterson et al., 1948) as an estimation of percentage coverage (0, 5, 10, 20, 40, 60, 80, and 100) of rust pustules (uredinia) over the flag leaf. IR was scored as a host reaction to rust pustules and converted to a 0–1 scale (Roelfs et al., 1992). The lines showing the mixed response of moderately resistant to moderately susceptible or vice-versa, were considered as the fifth category other than mentioned in Roelfs et al. (1992). Therefore, five scoring categories considered for the evaluation were: Resistant (R) = 0.2, Moderately Resistant (MR) = 0.4, Mixed response (M) = 0.6, Moderately Susceptible (MS) = 0.8, and Susceptible (S) = 1. Data were recorded at weekly intervals for three times when the flag leaves of the susceptible checks showed a disease score of 60S (DS: 60; IR: S). Out of these multiple scores of a test line, the one with the score tending toward susceptibility was kept for the study. These were further considered for GWAS in each environment by combining the two measures into a single value as coefficient of infection (COI). It is the product of DS and IR on a 0–100 linear scale (Loegering, 1959; Roelfs et al., 1992). Genotypes with COI scores of 0 to 20 were considered as resistant, with score ≥ 60 as susceptible and the remaining as moderate (Table 1). COI was assumed to be suitable for GWAS and as a primary trait for the identification of significant marker-trait associations (MTAs) since it combines both the information from DS and IR for rust response (Yu et al., 2012; Gao et al., 2016, 2017; Mihalyov et al., 2017).

### Statistical Analysis

The phenotype data (IT, IR, DS, COI) for the three rusts were visualized and considered for studying Pearson’s correlation in R statistical programming. Correlation plots for each rust describing the correlation of disease scores between different pathotypes and environments were created using corrplot R package. Mean comparison tests were performed for IT and COI among population structure based groups using Levene’s test (Levene, 1960) dependent two-way *t*-test at a significance level of *P* < 0.05 in the R program. The normality of the original data was tested using the Shapiro-Wilk test in IBM SPSS Statistics v.22.

A Restricted Maximum Likelihood (REML) approach (Corbeil and Searle, 1976) in R package “lme4” (Bates et al., 2015) was used for estimating variance components for IR and DS from field experiments for the three rusts. A linear mixed model was fitted by considering the overall mean as fixed effect and other factors as random effects. The random effects included genotype (*g*), location (*e*), genotype × location interaction (*g* × e) and year (as replication, r). Following model was applied to estimate variance components:

$$y_{p\,q\,r}=\mu +{\text{g}}_{p}+{\text{r}}_{q}+e_{q}+{\text{ge}}_{p\,q}+{\text{e}}_{p\,q}$$

Where, *y*_*pq*_ is the observation for the genotype *p* at location *q* in season r, μ is the overall mean, g*_*p*_* the effect of the genotype *p*, e*_*q*_* the effect of location *q*, r*_*q*_* the effect of year (season) r, ge*_*pq*_* the interaction between accession *p* within location *q*, and e*_*pq*_* the residual.

Broad sense heritability (*H*^2^) was estimated by using the following equation:

$$H^{2}=\sigma_{g}^{2}/\left\{\sigma_{g}^{2}+\left(\sigma_{e}^{2}/t\right)+\left(\sigma_{g\times e}^{2}/t\right)+\left(\sigma_{e\,r\,r\,o\,r}^{2}/t\right)\right\}$$

Where, $\sigma_{g}^{2}$ is the genotypic variance, $\sigma_{e}^{2}$ is the environmental variance, $\sigma_{g\times e}^{2}$ is the genotype by environment interaction variance, and $\sigma_{e\,r\,r\,o\,r}^{2}$ is the residual error variance and *t* is the number of years for each location or location by year for the estimates of heritability across all environments. To estimate the stability of genotypes in response to YR, LR, and SR infection in their respective four environments, COI scores were used to perform GGE biplot (genotype × environment interaction) in R package GGEBiplotGUI (Frutos et al., 2014). The COI scores were reversed with reference to maximum score so that the resistant genotypes can have higher scores aiding the interpretation of stable resistant genotypes across environments.

### Linkage Disequilibrium (LD), Population Structure, and Genetic Diversity

For the complete RAMP, LD analysis was performed across A, B, and D genomes separately. Intra-chromosomal pairwise marker LD as squared allele-frequency correlations (*r*^2^) values were calculated in TASSEL v5.2 (Bradbury et al., 2007) using a sliding window approach with default parameters. As a function of genetic distance, the estimated *r*^2^− values for significant SNP marker pairs were plotted to understand the extent of LD. A second-degree “loess” function (Cleveland, 1981) in the R statistical program was fitted to estimate the rate of LD decay over genetic distance (cM). Critical *r*^2^− values were estimated as the 95^th^ percentile of square root transformed *r*^2^− values of the unlinked SNP marker pairs (Breseghello and Sorrells, 2006) showing a distance of more than 50 cM for each genome. It indicates the point beyond which the LD is caused by genetic linkage (Breseghello and Sorrells, 2006). The genetic distance at which LD fell below the critical *r*^2^− value was considered as the confidence interval (CI) of quantitative trait loci (QTL) in this study. In other words, the point of intersection between LD decay loess curve and critical *r*^2^ was used to adjust the QTL-CI in terms of genetic distance.

The population structure and genetic diversity for the RAMP were previously described by Kumar et al. (2020). Briefly, the population structure was estimated with LD-based pruned markers using a Bayesian based model. The optimal number of subpopulations (*i.e., K* = 2) was determined using parallel runs in STRUCTURE 2.3.4 (Pritchard et al., 2000) with the Linux based python program “StrAuto” (Chhatre and Emerson, 2017). In addition, principal component analysis was also performed on the study panel using 14,650 SNP markers using ‘prcomp’ function in the R statistical programming language.

### Marker-Trait Association Analysis

The seedling response under greenhouse condition and field evaluation for adult plant response were considered for finding out associations. In order to identify the loci associated with the response, 14,650 SNP markers and phenotypic trait values for seedling (IT) and adult plant (COI) responses were used to conduct genome-wide association analyses. For the estimation of marker-trait associations (MTAs), Genomic Association and Prediction Integrated Tool (GAPIT) (Lipka et al., 2012) was used by implementing compressed mixed linear model (CMLM) (Yu et al., 2006; Zhang et al., 2010) in R environment. A marker-based VanRaden kinship (K) matrix (VanRaden, 2008) for the 483 accessions was also generated using GAPIT. The K and Q (Kumar et al., 2020) matrices were considered as random and fixed components, respectively, to avoid any spurious associations caused by population structure. Some additional association testing models were analyzed to compare the observed probability deviations from the expected distribution based on the Q-Q plots. A general linear model with kinship only and no correction for population structure (GLM_K), GLM (GLM_PC3.K), and mixed linear model (MLM_PC3.K) with kinship and the first three principal components, MLM with kinship and correction for population structure (MLM_Q.K), and MLM with kinship only (MLM_K) were considered. The comparison was done based on *P-*values and respective Q-Q plots for the MTAs obtained from GAPIT. All the comparative Q-Q plots and circular Manhattan plots were generated using the CMplot R package^1^.

GWAS was conducted on each rust pathotypes separately to identify race-specific seedling resistance loci. In the case of identifying resistance loci in the adult plant stage, COI was considered for each rust in four environments. Each environment (Supplementary Table S2) was considered as a unique set of phenotypic data to be considered for GWAS. The percentage of phenotypic variation explained by the MTA (*R*^2^) was calculated as the difference of *R*^2^ without SNP from *R*^2^ with SNP of the GAPIT model (Abed and Belzile, 2019; Garcia et al., 2019). High stringency was observed in GAPIT for false discovery rate (FDR) adjusted *p*-values. SNP markers were declared significantly associated at *p* ≤ 0.001 {-log_10_(*p*) ≥ 3} in the selected model. Hence, a liberal approach similar to previous studies (Pasam et al., 2012; Zegeye et al., 2014; Gao et al., 2016; Visioni et al., 2018), was considered so as to reduce the chance of neglecting any significantly associated marker annotating for disease resistance. A putative QTL was designated for intra-chromosomal SNPs of the MTAs detected and fall within the range for QTL-CI defined by LD. A representative SNP for such putative QTL was selected with the lowest *p*-value as the most significantly associated SNP.

Significant markers observed were further subjected to *in silico* annotation. The flanking sequence of these markers was obtained from the EnsemblPlants database spanning 1 kb both upstream and downstream of the SNP position^2^. In order to obtain the reference physical map positions of these markers, the flanking sequence was used to make a query against IWGSC RefSeq v1.0 (Appels et al., 2018). The flanking region of the significant SNP was then used to explore candidate gene overlapping with this region by using JBrowse (Skinner et al., 2009) from URGI (Unité de Recherche Génomique Info/research unit in genomics and bioinformatics). Annotations of found overlapping candidate genes were obtained from IWGSC Annotation v1.1. For candidate genes with unavailable annotations, the orthologous genes from *Triticum urartu* and *Aegilops tauschii* with highly similar sequences were considered for prediction of gene function in wheat by implementing BLASTn^3^. Further, annotations for SNPs (e.g., intergenic variants) with no overlapping gene were searched using a similar approach. The annotations were then confirmed against the protein sequences for determining putative molecular functions in wheat using BLASTx with default parameters in the Blast2Go v5.2 tool (Conesa and Götz, 2008). This could provide aid in the identification of putative candidate genes for disease resistance.

## Results

### Phenotypic Evaluation of Seedling and Adult Plant Stage

The phenotypic data recorded for disease response to pathotypes of three *Puccinia* species on wheat at seedling stage (IT) under controlled conditions and adult plant stage under field conditions (IR, DS, and COI) have been provided in Supplementary Table S3. In the panel, rust gene postulation was successfully done in about 240 genotypes based on response to multi-pathotype testing at seedling stage. Known resistance genes for the three rusts were postulated mostly in varieties (139) followed by improved genotypes (93) and few genetic stocks (12) (Supplementary Table S3).

#### Seedling Response to *P. stritiformis* Pathotypes

**FIGURE 1:**
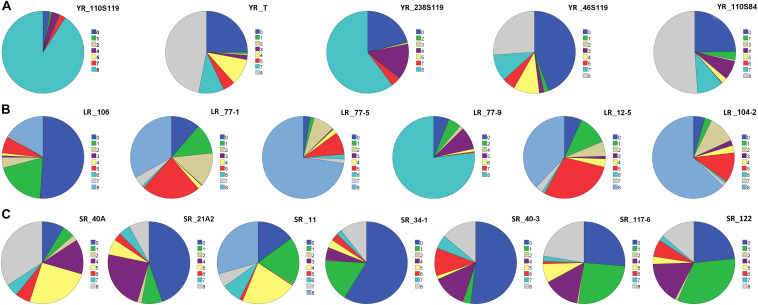
Pie chart representation of seedling response against **(A)** five pathotypes of stripe rust (YR), **(B)** six pathotypes of leaf rust (LR), and **(C)** seven pathotypes of stem rust (SR) of rust association mapping panel (RAMP). The color legend on the right side of each pie chart represents the infection type (IT) score. The magnitude of arc length is directly proportional to the frequency of genotypes showing corresponding IT scores.

On the linear scale of 0–9, IT scores ranged from 0 (as most resistant) to 8 (as susceptible) for the *Pst* pathotypes. Of the 5 *Pst* pathotypes, based on IT scores, 93, 63.7, 61.4, and 56.9% of the tested lines were found susceptible toYR_110S119, YR_238S119, YR_110S84, and YR_T, respectively (Table 1 and Figure 1A). This indicates YR_110S119 being the most virulent in the current study panel. On the other hand, pathotype YR_46S119 was found to be avirulent with 48.1% of the tested lines as resistant and 36.6% as susceptible. The mean IT scores for YR_110S119, YR_238S119, YR_110S84, YR_T, and YR_46S119 were 7.56, 5.64, 5.26, 5.11, and 3.51, respectively. Except for YR_46S119, the IT scores for other pathotypes were skewed toward susceptibility (Supplementary Table S4). The phenotypic correlation coefficients among pairs of the five pathotypes were observed to be significant (at *P* < 0.01, 0.001) and in the range of 0.09 (YR_110S119-YR_T) to 0.23 (YR_46S119-YR_110S84) (Supplementary Table S5 and Supplementary Figure S1).

#### Seedling Response to *P. triticina* Pathotypes

Based on the IT score with a range of 8, pathotype LR_106 was found to be relatively avirulent based on response to the current study panel. More than two-third (75.7%) of the tested lines were found resistant against LR_106 (Table 1 and Figure 1B). Among other *Pt* pathotypes, LR_77-9 was the most virulent followed by LR_77-5 and LR_104-2 with 77.3, 74.3, and 64.1% of the tested lines to be scored as susceptible, respectively. LR_12-5 and LR_77-1 were found to have an intermediate virulent response on the tested lines (Table 1). Mean IT scores for LR_106, LR_77-5, LR_104-2, LR_77-9, LR_12-5, and LR_77-1 were 1.87, 6.68, 6.10, 6.80, 5.00, and 4.43, respectively, with skewness toward susceptibility except for LR_106 (Supplementary Table S4). Significant correlation coefficients (at *P* < 0.001) among pairs of six *Pt* pathotypes ranged from 0.26 (LR_106-LR_77-9) to 0.57 (LR_106-LR_77-1) (Supplementary Table S5 and Supplementary Figure S1).

#### Seedling Response to *P. graminis* Pathotypes

Of the 7 *Pgt* pathotypes, none of them were found to be virulent on a range of 8 IT score. SR_40A can be designated as mildly virulent where only 39.2% of the tested lines were susceptible. For pathotypes SR_21A2, SR_34-1, SR_40-3, SR_117-6, and SR_122, resistant reactions were observed in 54.7, 75.9, 55.0, 52.8, and 57.6% genotypes. For SR_11, the tested lines showed similar type of response in resistant (34.3%), intermediate (31.0%), and susceptible (34.7%) category (Table 1 and Figure 1C). Mean IT responses for SR_40A, SR_21A2, SR_11, SR_34-1, SR_40-3, SR_117-6, and SR_122 were 5.299, 2.452, 4.206, 1.617, 2.721, 3.079, and 2.623, respectively, with skewness toward resistance except for SR_40A and SR_11 (Supplementary Table S4). Correlation coefficients between the seven *Pgt* pathotypes were found significant (at *P* < 0.001) and ranged from 0.34 (SR_21A2- SR 40-3) to 0.68 (SR_34-1-SR_117-6) (Supplementary Table S5 and Supplementary Figure S1).

#### Adult Plant Field Response to Stripe Rust

**FIGURE 2:**
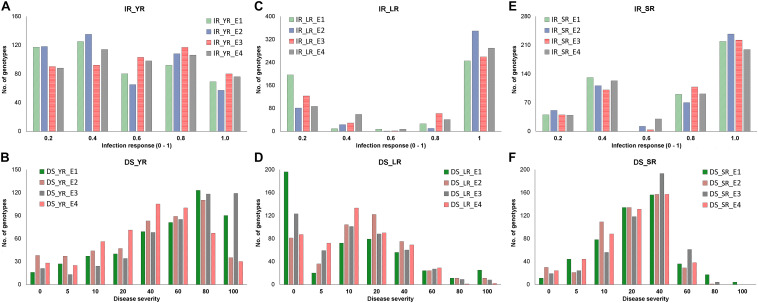
Distribution of adult plant stage disease response as infection response (IR) and disease severity (DS) in corresponding four environments (E1-E4) in the panel. Frequency of genotypes having IR (*y*-axis) for YR **(A)**, LR **(C)**, and SR **(E)** was recorded on the scale of 0-1 (*x*-axis), whereas the frequency of genotypes having DS (*y*-axis) for YR **(B)**, LR **(D)**, and SR **(F)** was recorded on the scale of 0-100 (*x*-axis). Each environment is represented with different colors as indicated by the color legend.

**FIGURE 3:**
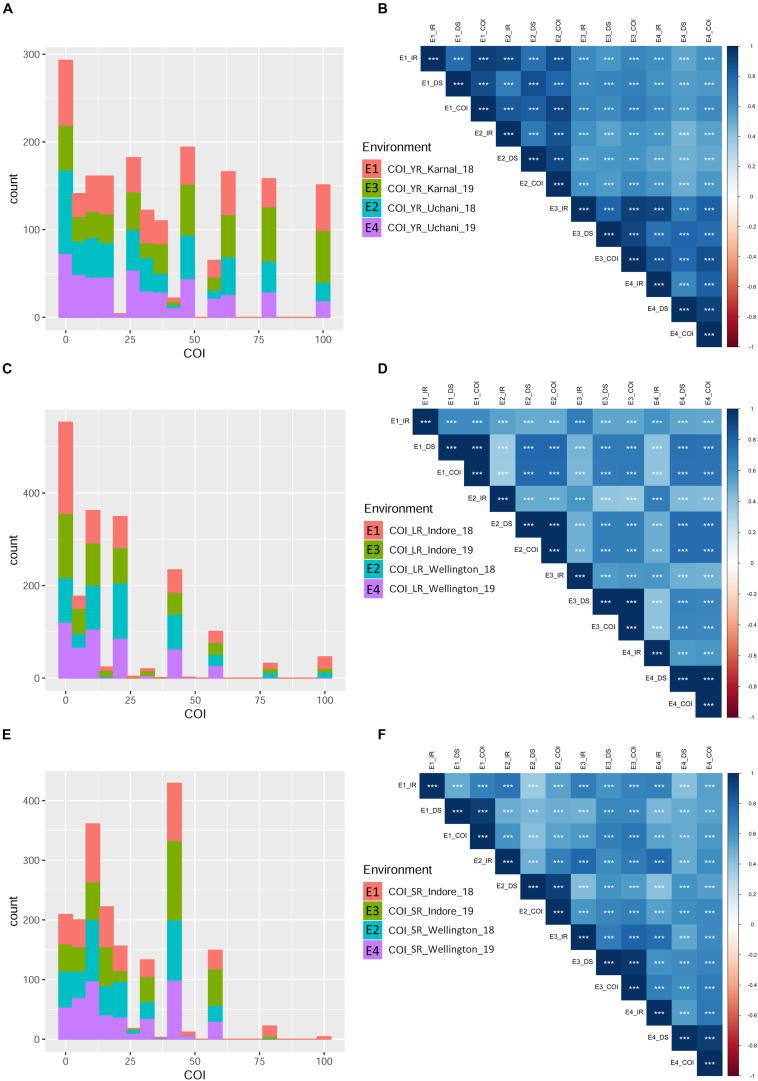
Phenotypic distribution based on the coefficient of infection (COI) in different environments for the three rust diseases. Different environments are represented with different colors in the bar-plot. Histograms **(A,C,E)** represent COI distribution for YR, LR, and SR, respectively. Pearson correlation coefficients (Supplementary Table S6) heat-map for infection response (IR), disease severity (DS), and COI between four environments are represented for YR **(B)**, LR **(D)**, and SR **(F)**. All correlation coefficients are highly significant at ^∗∗∗^*P* < 0.001.

Infection response (IR) with an average score of 0.5 was recorded at environments YR_E1 and YR_E2, whereas at YR_E3 and YR_E4, it was 0.6 (Supplementary Table S3). The distribution of IR was found to be non-biased for any single response category (Figure 2A). An average disease severity (DS) score of 62.5 at YR_E3 was found maximum among the four environments. DS with scores of 60-80 was found in most of the tested lines in all environments (Figure 2B and Supplementary Table S3). Based on the coefficient of infection (COI) as the disease response at four environments (COI_YR_E1-E4), stripe rust-resistant lines were found to be falling in more than one environment. In the RAMP, 37.9, 45.0, and 45.1% of the genotypes were recorded as resistant with a COI score from 0 to 20, in environments COI_YR_E1, COI_YR_E2, and COI_YR_E4, respectively (Table 1 and Figure 3A). Highly significant (at *P* < 0.001) Pearson’s correlation coefficients between IR, DS, and COI across four environments showed positive correlations (Supplementary Table S6 and Figure 3B). Strong correlation coefficients (*R* = 0.8–0.9) were observed between COI with IR and DS within the environment, whereas, it ranged from 0.6-0.8 between IR and DS. For COI among environments, strong values of *R* were detected between YR_E1 with YR_E2 and YR_E3 with YR_E4.

#### Adult Plant Field Response to Leaf Rust

An average high score of IR (0.8) and DS (21.7) was recorded in environment LR_E2 (Supplementary Table S3). The IR with score 1.0 (S) was seen in most of the genotypes irrespective of the environment (Figure 2C). DS with pustules coverage score 0–20 was present in more than 300 genotypes in each environment (Figure 2D). Likewise, more than seventy percent of the tested lines were observed to be resistant in the panel as per the COI score in all four environments (COI_LR_E1- E4) (Table 1 and Figure 3C). All correlations across four environments for IR, DS, and COI were found positive and highly significant (at *P* < 0.001) (Supplementary Table S6 and Figure 3D). Interestingly, unlike most genotypes featuring susceptible IR score, a very strong correlation (*R* = 0.99) was found between DS and COI per environment. This may suggest that a large proportion of the tested lines were able to resist the spreading of rust pustules. IR and DS showed a correlation coefficient value of *R* = 0.5–0.6 and COI with a correlation coefficient of 0.7, within and among the four environments.

#### Adult Plant Field Response to Stem Rust

Like leaf rust, stem rust also had a similar IR frequency distribution in the panel for the score 1.0 (S) with an overall average of 0.7 in each environment (Supplementary Table S3 and Figure 2E). Pustules coverage was found in a limited number of genotypes beyond the DS score of 40, where only 4 genotypes (HTW-6, IC212182, IC28617, and LGM69) attained rust coverage of 100 (Supplementary Table S3 and Figure 2F). The average DS was higher in SR_E3 followed by SR_E1. In the study panel, ∼62% of the tested lines were observed resistant to stem rust in environment COI_SR_E1 and E4 (Table 1 and Figure 3E). Results of correlations between DS and COI were found similar to those for leaf rust field response, whereas IR and DS showed *R* ranging from 0.4 to 0.6 within the environment. It varied from 0.5 to 0.7 for COI across the environments (Supplementary Table S6 and Figure 3F). All correlation coefficients were observed to be highly significant at *P* < 0.001.

For each of the three rusts in four environments, it was observed that the susceptible check lines present at regular intervals of the test genotypes showed maximum disease response with a score of 100S. This indicates that any possible variations observed in the reaction of the genotypes in RAMP would be genetic in nature and not due to the environment or inoculum load. The disease response at seedling and adult plant stages were observed to have less than 10% missing observations in the study panel. The distribution of phenotypic disease response (IT and COI) were found deviating from the normal distribution. No improvement in the normality was observed after using logarithmic and square root transformation. Therefore, the original data were used for subsequent genetic analyses.

#### Variance Components Estimation and Broad-Sense Heritability (*H*^2^)

Estimation and analysis of variance components, using a linear mixed model approach, revealed highly significant (*P* < 0.001) differences for IR, DS, and COI among the genotypes (*g)* across all environments in each rust type. Significant differences for location (*e*) and genotype-location interactions (*g* × *e*) were not observed in all cases (Table 2), therefore, by and large, indicating ample inoculum load and congenial atmospheric conditions for the appearance of the disease. High *H*^2^ estimates were observed across the four environments for each rust type, ranging from 0.78 to 0.92 for IR, 0.78 to 0.90, and 0.83 to 0.90 for COI (Table 2). The first two principal components explained together about 95.05, 88.68, and 86.76% of the total variation in GGE Biplot analysis for YR, LR, and SR field response, respectively. In reference to ranking resistant genotypes across environments, 22.15% (for YR), 61.49% (for LR), and 43.89% (for SR) were found in proximity to the ideal genotype (Supplementary Figure S2). In this study, a genotype would be considered as an ideal genotype, which would show a uniformly low and stable disease response in the tested four environments for all the three rusts.

**TABLE 2:** Variance component estimates for random variables for IR, DS, and COI across four environments per rust type^@^.

	Stripe rust			Leaf rust			Stem rust		
Subject	IR	DS	COI	IR	DS	COI	IR	DS	COI
$\sigma_{g}^{2}$	5.30e-02***	6.51e + 02***	6.63e + 02***	6.80e-02***	3.49e + 02***	3.47e + 02***	6.37e-02***	1.73e + 02***	2.23e + 02***
$\sigma_{e}^{2}$	4.77e-05^ns^	1.16e + 02***	5.80e + 01***	5.49e-03***	9.03e-02^ns^	1.16e-01^ns^	9.43e-05^ns^	1.08e + 01***	1.02e + 01***
$\sigma_{g\times e}^{2}$	0.00e + 00^ns^	0.00e + 00^ns^	0.00e + 00^ns^	1.31e-02***	1.99e + 00^ns^	4.60e + 00^ns^	0.00e + 00^ns^	2.50e + 01***	2.58e + 01***
$\sigma_{r}^{2}$	1.30e-03***	0.00e + 00^ns^	3.27e + 00**	0.00e + 00^ns^	4.90e + 00***	6.36e + 00***	0.00e + 00^ns^	6.85e-01^ns^	9.03e-01*
$\sigma_{e\,r\,r\,o\,r}^{2}$	2.14e-02^ns^	3.36e + 02^ns^	2.59e + 02^ns^	3.92e-02^ns^	1.49e + 02^ns^	1.51e + 02^ns^	2.24e-02^ns^	1.20e + 02^ns^	1.11e + 02^ns^
Mean	0.57	52.37	36.08	0.74	19.08	18.50	0.74	27.55	23.20
Minimum	0.2	0	0	0.2	0	0	0.2	0	0
Maximum	1	100	100	1	100	100	1	100	100
*H*^2^	0.91	0.82	0.88	0.78	0.90	0.90	0.92	0.78	0.83

**^@^IR, Infection response; DS, Disease severity; COI, Coefficient of infection. Asterisks ^∗^, ^∗∗^, and ^∗∗∗^ indicates P < 0.05, 0.01, and < 0.001, respectively, and ns = not significant.**

#### SNP Markers, Population Structure, and Linkage Disequilibrium

Of the 35K SNP markers, 14,650 polymorphic mapped markers were used for LD analysis and subsequently for GWAS. Genome D was found evident with lower marker density as compared to A and B genomes. Population structure analysis on RAMP was previously described by Kumar et al. (2020). The two subpopulations observed comprised of 106 (in SP1) and 377 (in SP2) genotypes (Supplementary Table S3). Genotypes that have been involved mostly in the breeding programs were observed as a major subpopulation. Principal component analysis (PCA) concurred with the result of STRUCTURE. Two separate clusters were observed in the PCA where PC1 (principal component 1) and PC2 contributed with 13.04 and 4.11%, of the total variation, respectively (Supplementary Figure S3). The effect of population structure was evident in the seedling response to multiple rust races. All pathotypes had a significant mean difference (at *P* < 0.05) for subpopulation based seedling disease response except for the pathotypes YR_110S119, YR_238S119, and YR_46S119 (Supplementary Table S7). The mean of IT scores for genotypes in subpopulation one (SP1) was higher than that of those in subpopulation two (SP2). This shows that in the current study genotypes were more resistant in SP2 than in SP1 to most of the pathotypes. A similar trend was observed in adult plant disease response where SP2 was significantly more resistant than SP1 based on COI (Supplementary Table S7). Therefore, the influence of population structure on rust infection was considered as covariates for subsequent association analyses.

**FIGURE 4:**
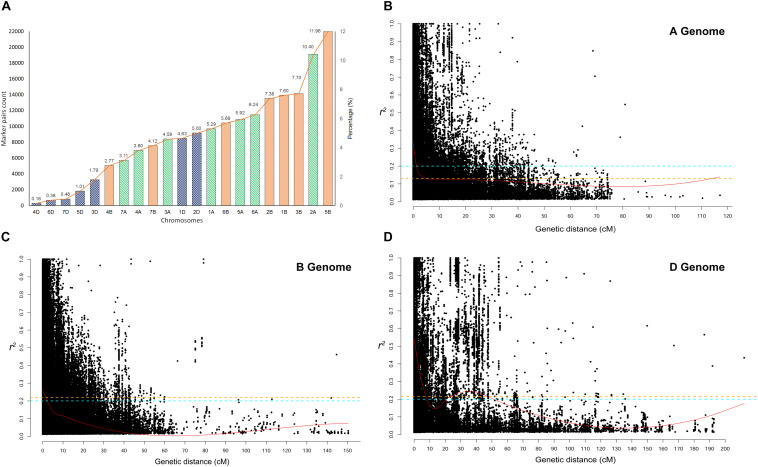
**(A)** Chromosome wise significant marker pairs that showed Linkage disequilibrium (LD) due to genetic linkage. LD decay plot is shown as a scatter plot of pairwise SNP LD *r*^2^− value over the genetic distance between each intra-chromosomal marker pairs for **(B)** A genome, **(C)** B genome, and **(D)** D genome. LOESS smoothening curve (red curved line) was fitted to the LD decay. Orange dash line represents the 95th percentile unlinked *r*^2^ line for **(A)** (*r*^2^ = 0.126), **(B)** (*r^2^* = 0.219), and **(D)** (*r*^2^ = 0.215) genomes. The Cyan dash line represents the critical *r*^2^ (=0.201) of the whole genome.

Linkage disequilibrium *r*^2^− values were estimated for the three genomes separately and for all chromosomes within each genome. The significant marker pairs at *P* < 0.01 were considered for the study. Genome A had 69.56% of significant marker pairs with an average *r*^2^− value of 0.250. Chromosome 2A had the highest (21.2%) and chromosome 4A had the lowest (10.1%) percentage of significant marker pairs of LD *r*^2^. Genome B had 73.67% of significant markers with an average *r*^2^− value of 0.212. All chromosomes of B genome had a similar percentage (∼16-18%) of significant marker pairs except for chromosome 4B (5.2%) and 7B (9.6%). Chromosome 1D (31.9%) and 2D (29.4%) had the highest number of significant marker pairs from the D genome with chromosome 4D (2.7%) bearing the lowest count (Figure 4A).

The critical *r*^2^-values were 0.126, 0.219, and 0.215 for genome A, B, and D, respectively. The difference in LD decay analysis was observed between A, B, and D genome. Whereas, predicted genome-wide LD decay was below critical *r*^2^ = 0.201. As LD beyond the critical value is considered to be caused by genetic linkage, chromosome-wise significant marker pairs with *r*^2^ > 0.201 were also estimated (Figures 4B–D). At a genome-wide scale, 36.35% of significant SNP pairs showed LD beyond critical *r*^2^ (> 0.201). Among the marker pairs in LD due to linkage, chromosome 5B contained the highest percentage (11.96%) of these markers, while chromosome 4D contained the lowest percentage (<< 1%). The percentage of these marker pairs in the A, B, and D genomes were 39.35, 47.21, and 13.43%, respectively. The map distance at which the fitted decay curve intersected with the critical *r*^2^ provided an estimate of QTL-CI. For genome A, B, and D, the estimated QTL-CI was 5, 3, and 8 cM, respectively (Figures 4B–D).

### Marker Trait Associations for Race-Specific Rust Response

**FIGURE 5:**
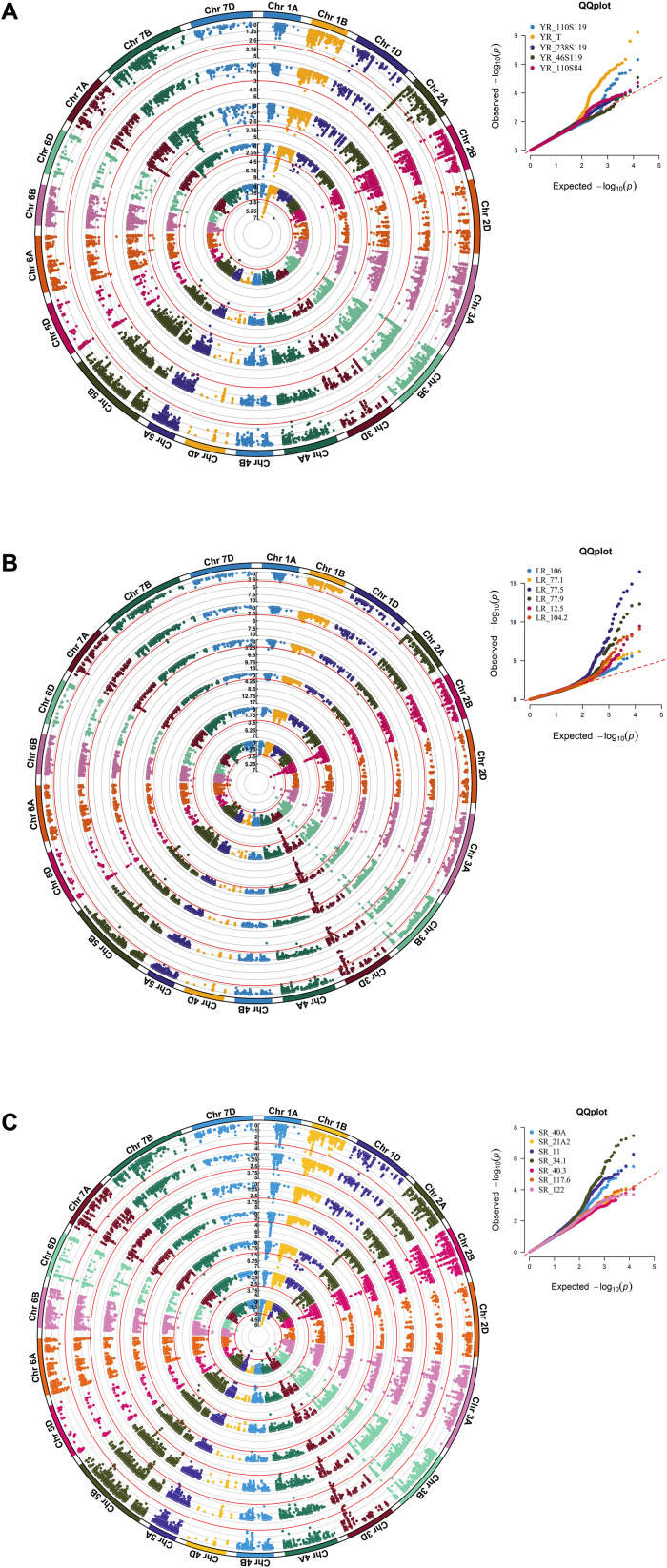
A circular Manhattan plot for significance [–log_10_(*p*-values)] of the association of 14,650 SNPs based on CMLM located on 21 chromosomes with the seedling disease responses against pathotypes of the three rusts. Associations for the **(A)** five YR pathotypes YR_110S119, YR_T, YR_238S119, YR_46S119, and YR_110S84; **(B)** six LR pathotypes LR_106, LR_77-1, LR_77-5, LR_77-9, LR_12-5, and LR_104-2; and **(C)** seven SR pathotypes SR_40A, SR_21A2, SR_11, SR_34-1, SR_40-3, SR_117-6, and SR_122 were plotted from inside to outside, respectively. A multi-track Q-Q plot for each case is presented at the right upper corner of the circular Manhattan plot. The threshold value at –log_10_(*p)* ≥ 3 is indicated as red-colored circle for each pathotype. A rectangular version of the plots is shown in Supplementary Figure S4.

A CMLM adjusted for population structure and relatedness (kinship) was selected to detect marker-trait associations (MTAs) (Figure 5). This approach gave a minimum deviation in the observed *p-*values from the expected values presented as Q-Q plots when compared with other tested models for controlling effects of population structure and relatedness (Supplementary File S1) for all GWAS analyses in this study. The number of MTAs found significant (at *p* < 0.001) for race-specific stripe rust (YR) response were 11, 25, 33, 62, and 99 for YR_46S119, YR_110S119, YR_238S119, YR_110S84, and YR_T, respectively. In case of leaf rust (LR), the counts were 41, 42, 47, 53, 69, and 100 for pathotypes LR_77-1, LR_106, LR_12-5, LR_104-2, LR_77-9, and LR_77-5, respectively. Stem rust (SR) pathotypes SR_40-3, SR_21A2, SR_122, SR_117-6, SR_40A, SR_11, and SR_34-1 were observed to have 12, 23, 28, 30, 64, 92, and 100 MTAs, respectively (Supplementary Table S8 and Supplementary Figure S4). Clearly, LR and SR possessed a large number of MTAs. *In silico* annotation of these MTAs associated with seedling disease response are also reported. Based upon these annotations, some of the commonly known resistance associated genes were found such as F-box/LRR-repeat protein At3g26922-like and protein 23, disease resistance RPP13-like protein 4, ABC transporter G family member 25, and more (Supplementary Table S8). The cluster of MTAs on the same chromosomes was considered as a putative QTL based on the confidence interval defined by LD. Therefore, MTAs within range of 5, 3, and 8 cM on chromosomes of genome A, B, and D, respectively were considered as a single putative QTL. The SNP marker with the lowest *p-*value (or most significant association) was used to represent such QTL obtained as the representative SNP. Based on this approach, YR pathotypes bearing 230 MTAs were grouped into 46 distinct loci. Similarly, MTAs for LR (352) and SR (349) pathotypes were grouped into 62 and 64 distinct loci, respectively (Supplementary Table S9). The MTAs observed can be considered in two categories. The first category comprised of loci found associated with two or more pathotypes and those associated with only one pathotype may fall in the second category. For YR pathotypes, nine loci mapped on chromosomes 1A (1), 1B (2), 1D (1), 2A (1), 2B (1), 3B (1), 5A (1), and 6A (1) were considered for the first category. Similarly, eighteen loci were observed on chromosomes 1A (1), 1B (1), 2B (2), 3A (2), 3B (4), 3D (5), 5B (2), and 7A (1) for LR pathotypes. Likewise, twenty six loci were observed for SR pathotypes on chromosomes 1A (3), 1B (2), 1D (2), 2A (1), 2B (2), 3A (3), 3B (4), 3D (3), 5A (1), 5B (2), 6B (1), 6D (1), and 7A (1) (Table 3).

**TABLE 3:** Putative QTL significantly (*p* < 0.001) associated with seedling stage resistance against at least two pathotypes for each rust pathogen.

Putative QTL*	Representative SNP						Associated SNPs	No. of MTAs	−log_10_(*p*)						
									YR pathotypes						
	SNP (35K)	Chrom^a^	Position (cM)	Allele^#^	MAF^b^	%PVE (*R*^2^)^$^			YR_46S119	YR_110S119	YR_238S119	YR_110S84	YR_T		
*QYr.ramp-1A.1*	AX-94448779	1A	57.21–58.94	T(128)/C(348)	0.27	5.24	AX-94838936	16	–	–	–	3.3448	8.2227		
*QYr.ramp-1B.1*	AX-95119512	1B	8.24–9.93	T(94)/C(388)	0.20	3.91	AX-94741250	9	–	6.3342	–	–	6.3456		
*QYr.ramp-1B.3*	AX-94768106	1B	23.90–26.22	T(106)/C(377)	0.22	4.81	AX-94500816 AX-94451305	75	–	5.6034	3.3111	–	7.6201		
*QYr.ramp-1D.2*	AX-94911855	1D	34.26–35.95	A(387)/G(91)	0.19	2.75	AX-95173669	3	–	–	–	3.4822	4.6689		
*QYr.ramp-2A.1*	AX-94691448	2A	0.00–1.59	A(29)/G(451)	0.06	4.27	AX-94448314	52	–	5.3691	–	4.7365	–		
*QYr.ramp-2B.4*	AX-94637676	2B	102.12–103.81	T(86)/C(396)	0.18	3.05	AX-94670661	3	–	–	3.3727	–	5.1017		
*QYr.ramp-3B.1*	AX-94573417	3B	0.79–2.24	A(394)/G(82)	0.17	2.67	AX-94632746	6	4.1367	–	–	–	4.5486		
*QYr.ramp-5A.1*	AX-95229410	5A	12.21–12.25	A(177)/G(302)	0.37	2.22	AX-94592812	2	3.0734	–	–	–	3.8789		
*QYr.ramp-6A.5*	AX-94926681	6A	217.8–221.75	T(367)/C(112)	0.23	4.04	AX-95094933	3	5.0844	–	3.6115	–	–		

									**LR pathotypes**						
									**LR_106**	**LR_12-5**	**LR_104-2**	**LR_77-9**	**LR_77-5**	**LR_77-1**	

*QLr.ramp-1A.2*	AX-94979481	1A	69.85–74.11	A(203)/G(277)	0.42	1.96	AX-94449010 AX-94897502	7	3.0647	–	–	3.2634	3.2910	–	
*QLr.ramp-1B.3*	AX-94904215	1B	24.64–26.22	C(158)/G(319)	0.33	2.30	AX-94908596 AX-94899483	6	3.0970	3.1977	–	3.2543	–	–	
*QLr.ramp-2B.3*	AX-94441179	2B	76.24–76.62	T(213)/C(267)	0.44	3.72	AX-94838738	11	3.1461	–	–	–	–	5.3291	
*QLr.ramp-2B.7*	AX-94554810	2B	103.81–104.59	T(172)/C(306)	0.36	1.56	AX-94705091 AX-94492853	8	4.2393	3.1461	3.1748	–	–	–	
*QLr.ramp-3A.6*	AX-95226287	3A	239.09	T(50)/C(426)	0.11	3.54–9.37	-	5	–	7.0931	7.5785	10.5717	12.9567	5.1067	
*QLr.ramp-3A.7*	AX-94883935	3A	250.41	A(428)/C(54)	0.11	3.73–10.34	AX-94477684	7	3.3933	5.5415	7.2823	12.1087	14.0301	5.6772	
*QLr.ramp-3B.5*	AX-95176613	3B	83.69–85.27	T(452)/C(25)	0.05	2.83	AX-94852024	22	–	3.8968	–	–	4.5076	–	
*QLr.ramp-3B.8*	AX-94871275	3B	202.68–204.37	C(424)/G(53)	0.11	3.06–8.87	AX-94915269	23	–	4.6575	6.5729	7.7139	12.3379	5.0884	
*QLr.ramp-3B.9*	AX-94977862	3B	226.84	T(106)/C(372)	0.22	2.38–3.05	–	3	–	–	3.5205	3.4349	4.8088	–	
*QLr.ramp-3B.11*	AX-94805563	3B	245.19–245.57	T(47)/C(434)	0.10	4.39–12.32	–	19	–	6.3846	9.1131	12.3215	16.503	6.2255	
*QLr.ramp-3D.3*	AX-94968108	3D	158.35	A(151)/G(326)	0.32	2.45–3.20	–	3	–	–	3.5997	3.8469	5.0179	–	
*QLr.ramp-3D.4*	AX-94874313	3D	160.44–167.71	A(414)/G(67)	0.14	3.51–7.84	–	10	–	5.2404	7.6624	9.1212	11.0555	5.205	
*QLr.ramp-3D.5*	AX-94436339	3D	169.4	T(416)/G(47)	0.12	3.41–10.97	–	10	–	5.1111	8.178	11.0265	14.8984	5.9561	
*QLr.ramp-3D.6*	AX-95224631	3D	174.29–178.60	A(55)/G(422)	0.12	2.56–10.09	AX-94681641	94	–	5.6468	8.0652	9.9604	13.8254	5.8167	
*QLr.ramp-3D.7*	AX-95082799	3D	191.16–194.75	T(255)/C(220)	0.46	3.64	AX-94958018	11	–	–	3.252	–	5.6019	–	
*QLr.ramp-5B.2*	AX-94673667	5B	163.54	A(332)/G(149)	0.31	2.15–3.94	–	4	–	3.4472	4.3824	4.8432	6.0107	–	
*QLr.ramp-5B.7*	AX-94480370	5B	270.33–272.82	T(433)/C(40)	0.09	2.10–3.75	–	14	–	–	–	3.0122	5.7506	–	
*QLr.ramp-7A*	AX-95233333	7A	27.21–29.90	T(444)/C(36)	0.08	3.45	AX-94644716	11	–	–	3.6353	4.6082	–	–	

									**SR pathotypes**						
									**SR_40-3**	**SR_21A2**	**SR_122**	**SR_117-6**	**SR_40A**	**SR_11**	**SR_34-1**

*QSr.ramp-1A.1*	AX-94448779	1A	58.79–61.06	T(128)/C(348)	0.27	2.57	AX-94432182 AX-94979364 AX-94405956 AX-94963297	17	–	–	3.3467	3.8465	4.7283	4.6978	3.0231
*QSr.ramp-1A.2*	AX-94467908	1A	72.25–74.86	A(91)/C(387)	0.19	3.26	AX-94845461 AX-95080736 AX-94627776	24	3.6987	–	3.3512	–	3.1361	4.2983	7.2645
*QSr.ramp-1A.3*	AX-94976474	1A	86.34–88.71	A(374)/C(100)	0.21	1.75	AX-94601315	2	–	–	3.4656	3.9451	–	–	–
*QSr.ramp-1B.3*	AX-94567638	1B	15.65	A(391)/G(91)	0.19	2.05	AX-94518139	6	–	–	–	–	3.0735	3.5196	–
*QSr.ramp-1B.4*	AX-94608698	1B	23.90–24.64	A(333)/G(145)	0.30	2.20–4.01	AX-95166367 AX-95124337	52	–	–	3.674	–	5.4927	6.2792	–
*QSr.ramp-1D.2*	AX-95015596	1D	30.82–35.95	A(90)/C(392)	0.19	1.59–2.89	AX-94722864	6	–	–	–	–	3.6869	4.7119	–
*QSr.ramp-1D.3*	AX-94546280	1D	72.10–76.08	T(91)/C(368)	0.20	2.61	AX-94693694	2	–	–	3.344	–	–	4.3155	–
*QSr.ramp-2A.1*	AX-94850836	2A	1.59	A(136)/G(344)	0.28	1.93–2.83	AX-94856367	59	–	3.5327	–	–	–	–	6.4025
*QSr.ramp-2B.5*	AX-95180258	2B	89.05	A(395)/G(84)	0.18	1.92–3.37	AX-94621294	11	–	4.0995	–	–	–	–	7.4726
*QSr.ramp-2B.6*	AX-94778579	2B	102.12–104.59	C(108)/G(370)	0.23	1.71	AX-94425581 AX-95017376 AX-94492853	11	–	3.3367	-	3.5125	-	3.8347	4.1357
*QSr.ramp-3A.1*	AX-94407346	3A	70.27–74.06	T(388)/G(87)	0.18	2.55	AX-94518742	6	–	–	–	3.0052	–	–	5.8434
*QSr.ramp-3A.6*	AX-95226287	3A	239.09	T(50)/C(426)	0.11	1.38–2.93	–	6	3.2747	3.032	3.1341	3.5889	4.0977	4.7693	–
*QSr.ramp-3A.7*	AX-94883935	3A	250.41	A(428)/C(54)	0.11	1.80–2.53	–	2	–	3.0239	–	–	–	4.2038	–
*QSr.ramp-3B.1*	AX-94573417	3B	0.79	A(394)/G(82)	0.17	1.79–2.55	–	2	–	–	–	–	3.4756	4.2301	–
*QSr.ramp-3B.3*	AX-94779453	3B	83.69–85.27	T(348)/C(125)	0.26	1.40	AX-94902398	2	–	–	–	–	–	3.2842	3.4922
*QSr.ramp-3B.6*	AX-94871275	3B	202.68–204.37	C(424)/G(53)	0.11	1.53–2.54	AX-94978418	4	–	–	–	3.5242	4.6767	3.5767	–
*QSr.ramp-3B.7*	AX-94805563	3B	245.19–245.57	T(47)/C(434)	0.10	1.67–3.46	AX-94989774	8	–	3.7911	3.6742	4.1798	4.2937	5.5216	–
*QSr.ramp-3D.2*	AX-94874313	3D	160.44–167.71	A(414)/G(67)	0.14	1.51–3.51	AX-94391473	6	3.2765	–	3.5944	3.4926	4.161	4.1827	–
*QSr.ramp-3D.3*	AX-94436339	3D	169.4	T(416)/G(47)	0.12	1.39–3.25	AX-94962712	8	3.7705	–	3.525	3.2463	4.0488	5.2282	–
*QSr.ramp-3D.4*	AX-94681641	3D	174.67–178.60	C(415)/G(54)	0.12	1.33–3.10	AX-94862539	29	–	–	3.3016	4.0116	3.7167	5.0067	–
*QSr.ramp-5A.3*	AX-94472861	5A	90.15–91.37	A(49)/G(432)	0.10	1.88	AX-95107770	2	–	–	–	3.1316	3.6164	–	–
*QSr.ramp-5B.10*	AX-94459834	5B	233.87	C(79)/G(393)	0.17	2.40	AX-95100204	2	–	–	3.197	–	–	4.0229	–
*QSr.ramp-5B.11*	AX-94677476	5B	270.33	T(114)/C(366)	0.24	1.61–3.11	–	2	–	–	–	–	3.1796	5.0269	–
*QSr.ramp-6B.2*	AX-94426734	6B	61.67–62.83	T(395)/C(88)	0.18	2.38	AX-94735973 AX-95023059	15	–	3.8498	–	–	–	3.9954	3.6213
*QSr.ramp-6D*	AX-94409568	6D	17.65–18.61	C(97)/G(386)	0.20	2.18	AX-94413225 AX-94803211	4	–	–	3.7012	–	3.2614	3.7021	–
*QSr.ramp-7A.1*	AX-95092895	7A	29.86–33.34	T(290)/C(191)	0.40	2.66	AX-94634646 AX-95251246	3	3.0999	–	3.0966	–	–	4.3851	–

*Remaining QTL associated with single pathotype is provided in Supplementary Table S9. ^#^The numbers in parenthesis indicate the frequency of the allele in the panel;^$^*percentage of phenotypic variation explained (PVE, R^2^). ^*a*^Chrom, Chromosome. ^*b*^MAF, Minor allele frequency.*

Several representative SNPs were observed for more than one rust type. Among those SNP markers AX-94573417, AX-94677476, and AX-94842331 representing loci for YR and SR seedling response had a bidirectional allelic effect. Whereas, AX-94871275, AX-94874313, AX-94883935, AX-94977862, and AX-95226287 had unidirectional allelic effect while representing loci for LR and SR (Supplementary Tables S8, S9). With respect to YR, only *QYr.ramp-1B.3* was observed in more than 2 pathotypes *viz*. YR_110S119, YR_238S119, and YR_T, also having the maximum number of MTAs. Loci *QYr.ramp-2A.1* had the second most numbers of MTAs found after *QYr.ramp-1B.3.* The most significant association among the nine QTL were observed for YR_T, more than twice (Table 3). In the case of LR, *QLr.ramp-3A.7* was seen associated with all the 6 pathotypes. Six loci *QLr.ramp-3A.6*, *QLr.ramp-3B.8*, *QLr.ramp-3B.11*, *QLr.ramp-3D.4*, *QLr.ramp-3D.5*, and *QLr.ramp-3D.6* were associated with all LR pathotypes except for LR_106, located on group 3 chromosomes. Most significant associations were present for LR_77-5 at more than four loci. The highest number of MTAs were found located on chromosome 3D at 174.29–178.60 cM (Table 3). For SR pathotypes, seven loci were found associated with more than 3 but limited to 6 pathotypes. These loci were present on chromosomes 1A (*QSr.ramp-1A.1, QSr.ramp-1A.2*), 3A (*QSr.ramp-3A.6*), 3B (*QSr.ramp-3B.7*), and 3D (*QSr.ramp-3D.2, QSr.ramp-3D.3, QSr.ramp-3D.4*). Most significant association was seen for SR_34-1 at more than two loci (Table 3).

When compared to other YR seedling resistance loci, *QYr.ramp-1A.1* exhibited a higher level of contribution to IT with phenotypic variance (*R*^2^) of 5.24% being associated with YR_110S84 and YR_T. Followed by *QYr.ramp-1B.3* accounting for *R*^2^ of 4.81% in association with YR_110S119, YR_238S119, and YR_T. Thirty-seven loci were associated with response to single YR pathotype, including 12 loci each with YR_238S119 and YR_T, 8 loci with YR_110S84, 4 loci with YR_46S119, and single loci with YR_110S119 (Supplementary Table S9). Similarly, of LR seedling resistance loci, *QLr.ramp-3B.11* exhibited the most contribution to IT within an *R*^2^ range of 4.39–12.32% in association with all pathotypes but LR_106. Also in decreasing order, *QLr.ramp-3D.5, QLr.ramp-3A.7*, and *QLr.ramp-3D.6* contributed to IT exhibiting a range of *R*^2^ from 3.41 to 10.97%, 3.73 to 10.34%, and 2.56 to 10.09%, respectively, associated with multiple LR pathotypes. Whereas, *QLr.ramp-5D* contributed with *R*^2^ of 6.86% and significantly associated (−log_10_*p* = 9.4711) with single pathotype LR_12-5. Forty four loci were associated with response to single LR pathotype, including 12 loci with LR_106, 10 loci with LR_77-9, 9 loci with LR_77-5, 6 loci with LR_12-5, 4 loci with LR_104-2, and 3 loci with LR_77-1 (Supplementary Table S9). In SR seedling resistance, loci *QSr.ramp-1B.4* was accounted for maximum contribution to IT with *R*^2^ ranging from 2.20 to 4.01% and was associated with SR_122, SR_40A, and SR_11. Another locus *QSr.ramp-1A.2* demonstrated a considerable *R*^2^ of 3.26% being associated with five SR pathotypes showing the most significant association with SR_34-1. Thirty-eight loci were associated with response to single SR pathotype, including 10 loci with SR_11, 7 loci each with SR_40-3 and SR_122, 6 loci with SR_21A2, 5 loci with SR_117-6, and 3 loci with SR_34-1 (Supplementary Table S9).

### Marker Trait Associations for the Three Rusts Field Response

**FIGURE 6:**
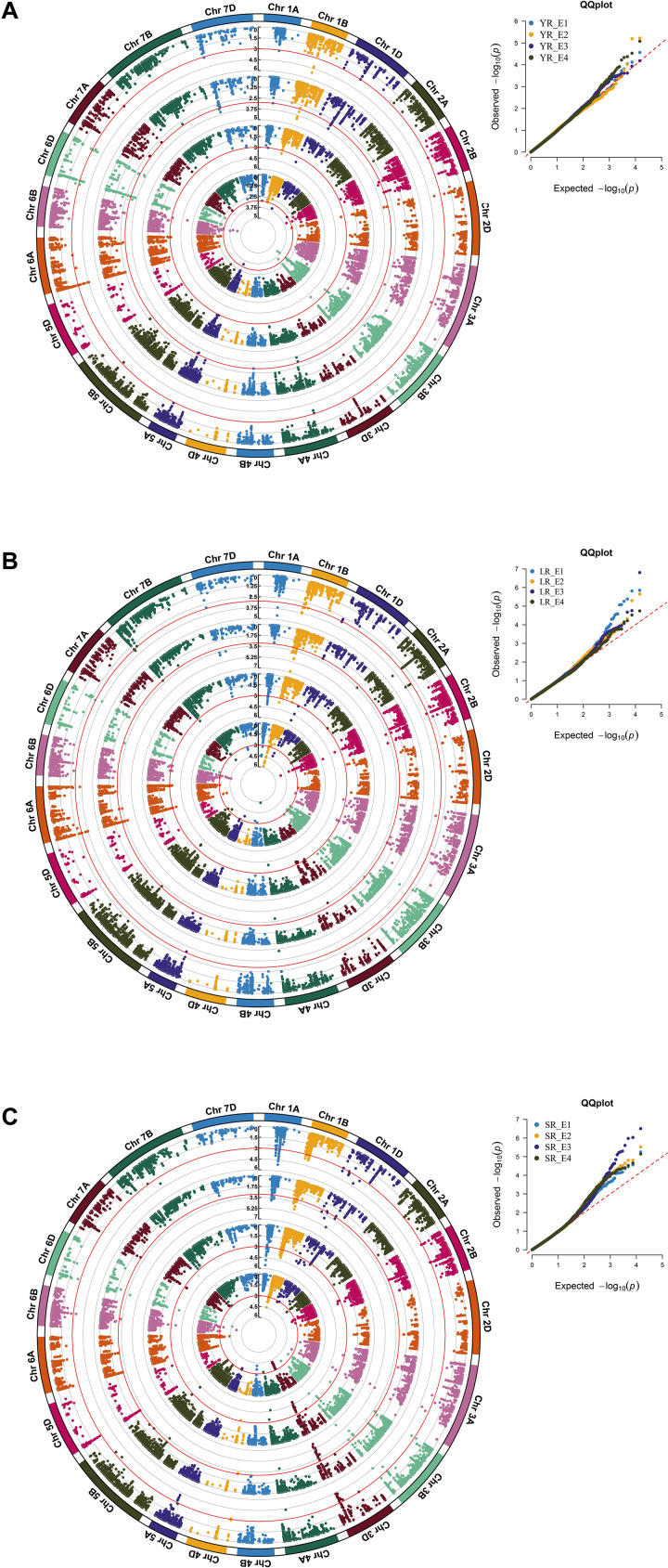
A circular Manhattan plot for significance [–log_10_(*p*-values)] of the association of 14,650 SNPs based on CMLM located on 21 chromosomes with the adult plant disease responses in the respective four environments for the three rusts. Associations in the four **(A)** YR environments YR_E1, YR_E2, YR_E3, and YR_E4; **(B)** LR environments LR_E1, LR_E2, LR_E3, and LR_E4; and **(C)** SR environments SR_E1, SR_E2, SR_E3, and SR_E4 were plotted from inside to outside, respectively. A multi-track Q-Q plot for each case is presented at the right upper corner of the circular Manhattan plot. The threshold value at –log_10_(*p)* ≥ 3 is indicated as red-colored circle for each environment. A rectangular version of the plots is shown in Supplementary Figure S5.

Under natural disease pressure in respective environments, 78, 151, and 252 significant (at −log_10_*p* ≥ 3) MTAs were observed using the CMLM for YR, LR, and SR field response in adult plant stage, respectively (Figure 6, Supplementary Table S10, and Supplementary Figure S5). *In silico* annotation of these MTAs associated with adult plant disease response are also reported. Several resistance associated genes were found in adult plant responses such as E3 ubiquitin-protein ligase, NB-LRR protein, *Lr10* disease resistance locus receptor-like protein kinase, serine/threonine kinase protein, DEAD-box ATP- dependent RNA helicase, disease resistance protein RGA3, etc. (Supplementary Table S10). LD based putative QTL identification revealed 29 distinct loci on all chromosomes except for 3D, 4D, 5D, and 7B to be associated with YR response, where 16 loci were observed in more than one environment. In case of LR response, 45 distinct loci were observed on all chromosomes except for 2D, 4D, 5D, and 7A. For SR response, none of the 44 distinct loci were observed on chromosomes 2D, 5D, and 7D. Eighteen and twenty-seven loci were observed in more than one environment for LR and SR, respectively (Table 4 and Supplementary Table S11). Among the identified representative SNPs, haplotype analysis using Haploview v4.2 (Barrett et al., 2005) markers with LD value *r*^2^ > 0.8 were observed in the case of LR and SR but not in YR. For LR, only one set of marker pairs comprising two markers was in strong LD, whereas for SR, three sets of marker pairs comprising of 2, 7, and 11 SNPs were observed in LD (Supplementary Figure S6 and Supplementary Table S11).

**TABLE 4:** Putative QTL significantly (*p* < 0.001) associated with resistance at the adult plant stage observed in at least two environments corresponding to the three rust diseases.

Putative QTL*	Representative SNP						Associated SNPs	No. of MTAs	−log_10_(*p*)			
				
	SNP (35K)	Chrom^a^	Position (cM)	Allele^#^	MAF^b^	%PVE (*R*^2^)^$^			YR_E1	YR_E2	YR_E3	YR_E4
*QYr.ramp-1B.3*	AX-95222999	1B	24.64	A(405)/C(77)	0.16	2.15	AX-94903617	2	3.0259	3.3988	–	–
*QYr.ramp-1B.4*	AX-94515822	1B	61.73	C(36)/G(440)	0.08	2.42–3.55	AX-94765876	6	–	–	4.1825	5.0791
*QYr.ramp-1D.1*	AX-94508418	1D	3.38	T(448)/C(26)	0.06	2.14–2.44	–	2	3.4973	3.7765	–	–
*QYr.ramp-1D.3*	AX-94444583	1D	95.56–95.70	A(93)/G(386)	0.20	2.19	AX-94918964	4	–	–	3.6071	3.0063
*QYr.ramp-2B.3*	AX-94761935	2B	88.33	C(43)/G(440)	0.09	1.87–2.09	–	2	3.4389	–	3.1554	–
*QYr.ramp-2D.1*	AX-94842790	2D	25.72	T(22)/C(453)	0.05	1.81–2.59	–	5	3.0408	3.4617	3.4908	3.8662
*QYr.ramp-3B.3*	AX-94541758	3B	85.27	A(123)/G(348)	0.26	2.10–2.59	–	3	4.1191	3.333	–	–
*QYr.ramp-3B.4*	AX-94680284	3B	245.57	T(255)/C(221)	0.46	2.08–2.62	–	2	3.4187	4.0166	–	–
*QYr.ramp-4A.1*	AX-94437374	4A	56.59	A(33)/G(442)	0.07	2.19–2.27	–	4	–	–	3.6111	3.4591
*QYr.ramp-5A.2*	AX-95684352	5A	70.36–73.80	A(76)/G(400)	0.16	1.85–1.99	AX-95002679	3	3.2955	–	3.2128	–
*QYr.ramp-6B.4*	AX-95209190	6B	147.99	C(24)/G(401)	0.10	2.53–3.55	AX-94777981	4	4.5647	5.2201	–	–
*QYr.ramp-6D.1*	AX-94953259	6D	0	T(274)/G(192)	0.41	1.93–2.02	–	2	–	–	3.2415	3.1352
*QYr.ramp-6D.2*	AX-95215612	6D	17.65	C(87)/G(385)	0.18	1.80–2.39	AX-95632341	12	–	–	3.5407	3.6068
*QYr.ramp-6D.3*	AX-94385810	6D	173.03–175.55	T(37)/C(436)	0.09	2.20–2.53	–	4	–	–	3.6192	3.786
*QYr.ramp-6D.4*	AX-95176310	6D	183.03	A(415)/G(62)	0.13	2.09–2.22	–	2	–	–	3.4617	3.3988
*QYr.ramp-7D.2*	AX-95205886	7D	6.96–10.82	T(369)/C(107)	0.22	1.20–2.09	–	3	–	–	3.4674	3.1032
									**LR_E1**	**LR_E2**	**LR_E3**	**LR_E4**
*QLr.ramp-1A.2*	AX-95080736	1A	72.25–74.11	A(428)/G(45)	0.10	2.50–2.72	–	11	4.5164	4.098	–	4.5249
*QLr.ramp-1B.1*	AX-95092361	1B	0.79	A(296)/C(185)	0.38	1.77–2.59	AX-94456747, AX-95222141	5	–	3.5824	3.8295	3.7671
*QLr.ramp-1B.2*	AX-94692514	1B	8.24	C(266)/G(212)	0.44	2.55	AX-94997422	4	–	–	3.776	3.089
*QLr.ramp-1B.3*	AX-94517050	1B	24.64–26.22	C(425)/G(48)	0.10	5.03	AX-94571235, AX-94538509	26	5.8111	–	6.8053	3.0183
*QLr.ramp-1D*	AX-94541931	1D	3.38	A(360)/G(113)	0.24	2.35–3.37	AX-94641589	6	4.0322	5.3001	–	3.0538
*QLr.ramp-2B.7*	AX-94962080	2B	103.81–104.59	T(297)/G(181)	0.38	2.22	AX-95019501, AX-95149329	7	3.8429	3.7829	–	3.0912
*QLr.ramp-2B.8*	AX-94481202	2B	126.67	A(29)/G(448)	0.06	2.48–3.62	–	3	5.0863	5.6499	3.69	–
*QLr.ramp-3A.1*	AX-94394356	3A	30.91	A(340)/C(136)	0.29	1.90–1.94	–	2	–	3.3009	–	3.3312
*QLr.ramp-3B.6*	AX-94925132	3B	117.54	T(359)/C(105)	0.23	1.75–1.99	–	2	3.1401	–	3.0643	–
*QLr.ramp-4A.4*	AX-94708164	4A	214.72	A(131)/G(343)	0.28	2.44–3.61	–	4	5.8523	4.0148	3.9499	4.7453
*QLr.ramp-5A*	AX-94889598	5A	12.25–16.13	A(226)/C(256)	0.47	2.08	AX-95168494	2	3.6276	–	–	3.1295
*QLr.ramp-5B.2*	AX-94515669	5B	161.41–161.85	T(316)/C(156)	0.33	2.26	AX-94996993	4	–	3.7482	3.1212	–
*QLr.ramp-6A.1*	AX-94653398	6A	0.00–0.82	T(157)/G(316)	0.33	1.74–2.67	AX-94585745, AX-94800842	14	3.9121	3.5047	3.9293	3.7568
*QLr.ramp-6A.4*	AX-95147877	6A	217.80–221.75	T(87)/C(385)	0.19	1.83–2.04	AX-94411794, AX-95014734	6	3.1277	3.3368	–	3.5439
*QLr.ramp-6B.5*	AX-94638655	6B	142.73	T(265)/C(215)	0.45	1.75–2.53	–	2	4.294	3.027	–	–
*QLr.ramp-6B.6*	AX-94877284	6B	147.99–150.38	T(393)/C(76)	0.16	1.84–2.73	–	4	3.1583	4.5831	–	–
*QLr.ramp-7B.1*	AX-95109168	7B	1.72–2.51	A(286)/G(174)	0.38	1.69–2.21	AX-94767893, AX-94617870	5	3.8311	3.3579	3.0418	3.1978
*QLr.ramp-7D.2*	AX-94674448	7D	18.37	T(69)/C(408)	0.15	1.94–2.79	–	4	3.4277	–	4.0777	–
									**SR_E1**	**SR_E2**	**SR_E3**	**SR_E4**
*QSr.ramp-1A.1*	AX-94629244	1A	54.04–58.94	T(355)/C(122)	0.26	2.76–4.07	AX-94831912	16	–	5.5236	3.8788	3.9088
*QSr.ramp-1A.2*	AX-95092467	1A	74.11–74.86	T(389)/C(92)	0.19	3.80	AX-94739433, AX-94845461	17	–	3.6209	3.579	5.1442
*QSr.ramp-1B.2*	AX-95108068	1B	8.24–9.93	A(376)/G(87)	0.19	2.19–2.63	AX-94602901	12	3.6636	3.7892	3.7174	3.5776
*QSr.ramp-1B.3*	AX-94518139	1B	15.65	C(384)/G(92)	0.19	2.06–2.46	–	3	–	3.5756	3.5198	3.0799
*QSr.ramp-1B.4*	AX-94608698	1B	24.64–26.22	A(333)/G(145)	0.30	1.81–2.83	AX-94414930, AX-94538509	34	4.4578	4.1201	4.6302	3.7215
*QSr.ramp-1D.1*	AX-94842940	1D	1.69	A(88)/G(394)	0.18	1.96–2.60	–	3	–	3.7503	3.3773	3.3816
*QSr.ramp-1D.2*	AX-94911855	1D	30.82–35.95	A(387)/G(91)	0.19	1.68–2.57	AX-94722864	8	3.1839	3.9222	4.0705	3.4796
*QSr.ramp-1D.3*	AX-94546280	1D	76.08	T(91)/C(368)	0.20	1.57–3.24	–	4	3.0095	3.3603	5.209	4.1243
*QSr.ramp-1D.4*	AX-94842331	1D	95.7	T(395)/C(88)	0.18	1.88–2.52	–	3	–	3.6553	3.2692	3.3673
*QSr.ramp-2B.6*	AX-94637676	2B	102.12–104.59	T(86)/C(396)	0.18	2.07–2.26	AX-94623285	5	3.2455	3.3308	–	3.0644
*QSr.ramp-2B.7*	AX-94784324	2B	126.67	T(215)/C(265)	0.45	1.71	AX-94481202	2	3.461	3.465	–	–
*QSr.ramp-3A.6*	AX-95226287	3A	239.09	T(50)/C(426)	0.11	1.83–3.83	–	4	3.4185	4.0281	6.0299	4.1187
*QSr.ramp-3A.7*	AX-94883935	3A	250.41	A(428)/C(54)	0.11	2.39–3.16	–	3	–	3.4977	5.1024	3.4855
*QSr.ramp-3B.1*	AX-94479164	3B	0.79	A(106)/G(375)	0.22	1.89	AX-94573417, AX-94581339	5	3.5179	3.3687	3.2188	3.439
*QSr.ramp-3B.6*	AX-94915269	3B	204.37	C(428)/G(50)	0.10	2.43–2.67	–	5	–	3.5456	4.4059	3.7619
*QSr.ramp-3B.7*	AX-94805563	3B	245.19–245.57	T(47)/C(434)	0.10	1.96–4.18	–	5	3.6265	4.5159	6.5041	4.532
*QSr.ramp-3D.1*	AX-94901185	3D	76.05	T(374)/C(88)	0.20	2.02–2.06	–	2	–	3.0328	–	3.0419
*QSr.ramp-3D.2*	AX-94874313	3D	160.44–167.71	A(414)/G(67)	0.14	2.09–2.40	AX-94391473	6	4.1716	–	5.2554	3.4058
*QSr.ramp-3D.3*	AX-94436339	3D	167.71–169.40	T(416)/G(47)	0.12	2.28–3.79	–	9	4.1324	4.7983	5.9778	4.2819
*QSr.ramp-3D.4*	AX-95224631	3D	175.97–178.60	A(55)/G(422)	0.12	1.83–3.53	AX-94681641, AX-94724171	52	4.1038	4.3769	5.6016	4.3799
*QSr.ramp-4A.2*	AX-94897136	4A	188.85	A(72)/G(392)	0.16	2.27–3.33	–	3	–	4.646	3.8328	4.3236
*QSr.ramp-4A.3*	AX-94508043	4A	214.31	T(67)/C(415)	0.14	2.31	AX-94630583	2	3.1965	3.387	–	–
*QSr.ramp-4D.1*	AX-94516693	4D	0	T(352)/C(125)	0.26	1.79–2.10	–	2	–	–	3.137	3.0959
*QSr.ramp-5A.1*	AX-94560538	5A	59.99	T(441)/G(35)	0.08	2.93	AX-94519690	9	3.2318	–	–	4.1142
*QSr.ramp-5B.10*	AX-94459834	5B	233.87	C(79)/G(393)	0.17	1.70–3.06	–	3	–	4.3091	3.0007	3.4288
*QSr.ramp-6B.2*	AX-94426734	6B	62.83	T(395)/C(88)	0.18	1.88–2.52	–	3	–	3.6553	3.2692	3.3673
*QSr.ramp-6D*	AX-94805944	6D	18.24–18.61	T(68)/C(413)	0.14	2.56	AX-94409568	3	4.5629	3.6741	3.9384	–

**^∗^Remaining QTL associated with single environment are provided in Supplementary Table S11. ^#^The numbers in parenthesis indicate the frequency of the allele in the panel.****^$^****Percentage of phenotypic variation explained (PVE, R^2^). ^*a*^Chrom, Chromosome. ^*b*^MAF, Minor allele frequency.**

For YR field response, *QYr.ramp-2D.1* was the only locus observed in all the four environments (YR_E1-E4), whereas the remaining 15 loci represented at least two environments. Most significant association was seen for *QYr.ramp-6B.4* in YR_E2 followed by *QYr.ramp-1B.4* in YR_E4. For LR field response, three loci were seen associated in all four environments (LR_E1-E4) namely *QLr.ramp-4A.4*, *QLr.ramp-6B.3*, and *QLr.ramp-7B.1*. Most significant association was found for *QLr.ramp-1B.3* in LR_E3 which also holds the most numbers of MTAs observed. In the case of SR, the most number of loci (nine) affiliated with all four environments (SR_E1-E4) were observed. Amongst these and other loci, *QSr.ramp-3D.4* holds the most number of MTAs followed by *QSr.ramp-1B.4*, where the most significant association was observed for *QSr.ramp-3B.*7 in SR_E3 (Table 4).

Among YR field response associated loci, *QYr.ramp-6B.4* and *QYr.ramp-1B.4* exhibited similar and higher contribution for COI with *R*^2^ ranging from 2.42 to 3.55% in at least two environments. Thirteen loci were associated with YR field response in single environment, including 6 loci in YR_E4, 3 loci in YR_E3, and 2 loci each in YR_E2 and YR_E1 (Supplementary Table S11). For LR field response, of 45 loci, *QLr.ramp-1B.3* accounted for the higher level of *R*^2^ of 5.03% while contributing to COI. To LR field response in single environment, 27 loci were seen associated where 10 loci in LR_E1, 7 loci in LR_E2, 6 loci inLR_E3, and 4 loci in LR_E4 were observed (Supplementary Table S11). In case of SR field response, *QSr.ramp-3B.7* explained the higher level of contribution for COI with *R*^2^ ranging from 1.96 to 4.18%. Seventeen loci were associated with a single environment, where 8 loci in SR_E1, 4 loci each in SR_E2 and SR_E4, and single loci in SR_E3 were observed (Supplementary Table S11).

Like seedling response, some representative SNPs were found common between two rust type field responses. Among those SNPs, AX-94638655 and AX-95176310 representing loci for YR and LR; AX-94874313 and AX-94877284 representing loci for LR and SR showed a bidirectional allelic effect. Whereas, AX-94805944 representing loci for LR and SR showed unidirectional allelic effect (Supplementary Tables S10, S11). Despite having different representative SNP markers, certain QTL observed for resistance in the adult plant stage were seen to be sharing QTL-CI based similar to identical genomic region with those observed for seedling stage resistance (Supplementary Table S12). In such case, QTL observed for both seedling and adult plant disease responses were considered as ASR loci and those observed only for the adult plant disease response were considered as APR loci. There were few ASR loci which had identical representative SNP marker for the QTL observed in seedling and adult plant stages. Of 12 YR ASR loci, *QYr.ramp-3B.4* was represented by SNP AX-94680284. Similarly, of 15 LR ASR loci, *QLr.ramp-3B.3* and *QLr.ramp-3D.4* were represented by SNPs AX-94741529 and AX-94874313, respectively. In the case of SR, with the most number of ASR loci (31), there were fourteen such representative SNP markers (Supplementary Table S12). The most number of APR loci were observed against LR (30) disease response followed by that of YR (17) and SR (13). Several genomic regions in terms of QTL-CI were found common in more than one rust field response. These robust genomic regions bearing QTL-CI based co-localized ASR and APR loci can be harnessed for further exploration and usage (Table 5). Among these genomic regions, seven of them been found on chromosomes 1A, 1B, 1D, 2B, 6A, 6B, and 6D, had QTL for all the three rusts.

**TABLE 5:** Robust genomic regions observed in more than one rust type field response.

Chromosome	Position (cM)*	QTL for field response^$^			References^#^
			
		YR	LR	SR	
1A	**72.25–75.74**	*QYr.ramp-1A.2*	*QLr.ramp-1A.2*	*QSr.ramp-1A.2*	Rosewarne et al., 2012; Jighly et al., 2015; Laidò et al., 2015; Gao et al., 2016
1B	8.24–9.93	–	***QLr.ramp-1B.2***	*QSr.ramp-1B.2*	Singla et al., 2017
1B	**24.64–26.22**	*QYr.ramp-1B.3*	*QLr.ramp-1B.3*	*QSr.ramp-1B.4*	Schlegel and Korzun, 1997; Maccaferri et al., 2015a; Mago et al., 2005; Ren et al., 2009
1D	**1.69–3.38**	*QYr.ramp-1D.1*	***QLr.ramp-1D***	*QSr.ramp-1D.1*	Sambasivam et al., 2008; Zwart et al., 2010; Ren et al., 2012a; Vazquez et al., 2012; Gill et al., 2019
1D	95.56–95.70	*QYr.ramp-1D.3*	*–*	*QSr.ramp-1D.4*	Godoy et al., 2018
2A	83.23–83.60	*–*	*QLr.ramp-2A.3*	*QSr.ramp-2A.2*	
2A	176.07–179.61	***QYr.ramp-2A.4***	***QLr.ramp-2A.5***	*–*	Li et al., 2014; Zhang et al., 2017
2B	**102.12–104.59**	*QYr.ramp-2B.4*	*QLr.ramp-2B.7*	*QSr.ramp-2B.6*	Feng et al., 2015; Aoun et al., 2019; Liu et al., 2020
2B	126.67–126.67	*–*	***QLr.ramp-2B.8***	*QSr.ramp-2B.7*	
2B	182.52–182.52	*–*	*QLr.ramp-2B.9*	***QSr.ramp-2B.8***	
3A	84.43–84.43	*–*	***QLr.ramp-3A.3***	*QSr.ramp-3A.3*	
3A	248.16–250.41	*–*	*QLr.ramp-3A.7*	*QSr.ramp-3A.7*	
3B	245.19–245.57	*QYr.ramp-3B.4*	*–*	*QSr.ramp-3B.7*	
3D	160.44–167.71	*–*	*QLr.ramp-3D.4*	*QSr.ramp-3D.2*	
3D	167.71–169.40	*–*	*QLr.ramp-3D.5*	*QSr.ramp-3D.3*	
3D	175.97–178.60	*–*	*QLr.ramp-3D.6*	*QSr.ramp-3D.4*	Friebe et al., 1993; Jiang et al., 1993
4A	214.31–214.72	*–*	***QLr.ramp-4A.4***	***QSr.ramp-4A.3***	
4B	89.57–92.16	***QYr.ramp-4B***	***QLr.ramp-4B.2***	*–*	
6A	80.46–80.46	*QYr.ramp-6A.2*	***QLr.ramp-6A.2***	*–*	
6A	**215.37–221.75**	*QYr.ramp-6A.5*	***QLr.ramp-6A.4***	***QSr.ramp-6A.5***	Muleta et al., 2017a; Kolmer et al., 2019; Sharma et al., 2019
6B	109.86–109.86	***QYr.ramp-6B.2***	***QLr.ramp-6B.4***	*–*	
6B	142.73–142.73	***QYr.ramp-6B.3***	***QLr.ramp-6B.5***	*–*	
6B	**147.99–150.38**	***QYr.ramp-6B.4***	***QLr.ramp-6B.6***	***QSr.ramp-6B.3***	Santra et al., 2008; Maccaferri et al., 2015b; Bulli et al., 2016; Nirmala et al., 2016; Li et al., 2020
6D	**17.65–18.61**	*QYr.ramp-6D.2*	***QLr.ramp-6D.1***	*QSr.ramp-6D*	Mebrate et al., 2008; Gebrewahid et al., 2020
6D	183.03–183.03	***QYr.ramp-6D.4***	***QLr.ramp-6D.3***	*–*	

**^∗^Regions found common for all three rusts are highlighted in bold.****^$^****QTL highlighted in bold represents the APR loci. ^#^Details are given in the text.**

### Pyramiding Effects of Favorable Alleles on Field Response

**FIGURE 7:**
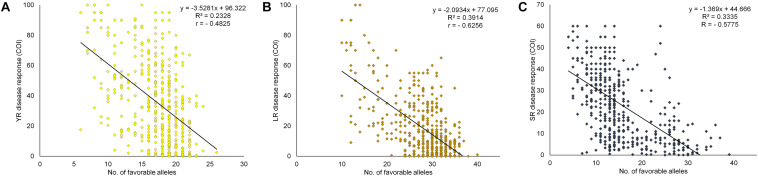
Linear regression plots of field disease response toward **(A)** YR, **(B)** LR, and **(C)** SR, to favorable alleles of representative SNPs of identified QTL in each of the 483 genotypes in the panel and averaged COI score over different environments. All regressions were highly significant at *P* < 0.0001.

The cumulative effect of favorable MTAs for field resistance was studied based on the estimated number of favorable alleles in each specific genotype. For this, SNPs representing the distinct loci for each rust were considered. Alleles associated with a reduction in disease response were considered as favorable alleles at each locus of the representative SNP. With 29 SNPs for YR, the number of favorable alleles ranged from 6 to 26. Similarly, for LR and SR, it ranged from 10 to 40 and 4 to 39 with 45 and 44 SNPs, respectively (Supplementary Table S13). The accumulating resistant alleles were expected to be strongly correlated with the disease response. A significant negative correlation (at *p* < 0.0001) was observed in the case of YR (-0.4825), LR (-0.6256), and SR (-0.5775) between the number of favorable alleles in individual genotypes and averaged disease response under their respective environments. This observation was further supported by the linear regression coefficients (R^2^) found significant at *p* < 0.0001 in all three cases (Figure 7). The total SNPs (118) estimated as field response based QTL representative markers were examined using a hierarchical clustering approach by selecting 72 genotypes having average COI score ≤ 20 in all the three rusts. Clearly, these genotypes were found to have the most number of favorable alleles compared to moderately resistant to susceptible genotypes except for some resistant genotypes *viz.* E3257, H957, HUW679, PBW138, IC321918, and LBRL4. The genotypes were grouped in same clusters based on the SNP array pattern having favorable alleles in those genotypes (Supplementary Figure S7). Pedigree based closeness was also visible in genotypes showing allelic similarity in terms of favorable alleles, e.g., HPW360-HPW361, DBW50-DBW88, and VL802-VL804. The present finding provides valuable information to the wheat breeders in deciding the selection of parents for the crossing program, particularly those to be utilized as source of rust resistance. Incorporation of diverse alleles can be ensured if parents are chosen from the different clusters (Supplementary Figure S7).

## Discussion

Crop scientists face dual challenges of increasing productivity and sustaining it by managing stresses occurring due to biotic and abiotic factors. Among biotic factors, the three rusts of wheat cause continuous threat through the rapid evolution of new races. The development and deployment of resistant cultivars is the most economical and environmentally safe method to control diseases. Primary gene pool including indigenous collections comprising of landraces, old cultivars and breeding lines are considered as a valuable genetic resource for providing new and durable resistance that can be exploited for the development of current day high-yielding varieties (Mujeeb-Kazi et al., 2013). Utilization of primary gene pool is advantageous over secondary (comprising of wild relatives) as they carry homologous genomic regions having a better opportunity as combiner with hexaploid wheat (Wulff and Moscou, 2014). To widen the spectrum of rust resistance in modern wheat varieties, finding lines having new sources of resistance along with newer alleles for known resistance genes, is the way forward. High throughput genotyping technologies coupled with bioinformatics enable us to mine valuable information from genome database paving the way for a better understanding of vast germplasm collections.

With this objective, a diverse panel of 483 genotypes found suitable for GWAS (Kumar et al., 2020) was utilized for identifying genomic regions associated with rust resistance in the form of diverse resistant alleles and their combinations for durable resistance. This panel comprised diverse genotypes both in temporal and spatial dimensions, *i.e.*, genotypes adapted to various agro-climatic conditions spanning India and varieties released during pre and post green revolution era. This panel encompassing of hitherto less engaged germplasm such as landraces offers a rich and new accessible source for disease resistance. Studies have supported the utilization of landraces for exploring novel resistant alleles (Muleta et al., 2017b; Pasam et al., 2017; Riaz et al., 2018). The same diverse panel was evaluated against all the three rusts without aiming any biasness toward specific rust. The phenotypic distribution of disease response did not follow a normal distribution, and original phenotypic data was utilized as such, similar to the previous studies (Gao et al., 2016; Edae et al., 2018). In this study, more than 50% of genotypes in the panel showed resistance to five out of seven SR pathotypes. Similar proportion of genotypes were found resistant to SR in four environments at the adult plant stage under field disease situations. This observation strongly suggested the presence of ASR loci in the panel. Whereas, in the case of LR, the presence of APR loci was supported by the fact that more than 70% of the lines were resistant in each of the four LR environments in spite of the panel showing susceptibility to five out of six LR pathotypes under seedling stage disease response. This was in concurrence with most number of ASR and APR loci observed against SR and LR disease response, respectively. Significant variations were observed among the genotypes in the panel as indicated by analysis of variance (ANOVA). High heritability estimates observed in this study for field disease responses were supported by significant positive correlations among traits and occurrence of consistent data recorded across environments. This indicated reproducibility of the data reliable for GWAS (Godoy et al., 2018).

Wheat breeder’s 35K array derived from wheat 820K SNP array provides breeder oriented informative markers (Allen et al., 2017). It has an added advantage of capturing information on germplasm derived from secondary and tertiary gene pools as the array is developed using exome capture of wheat and its wild relatives (Allen et al., 2017; Rasheed and Xia, 2019). In our study, LD decay was found strongly different in the three genomes with B genome to have the quickest decay followed by A genome, which is supported by previous reports (Gao et al., 2017; Riaz et al., 2018). This supports the practical use of D genome markers suitable for association tests even though less in number (Gao et al., 2017). D genome was found to have the highest LD than other genomes which has also been mentioned in several previous studies (Nielsen et al., 2014; Wang et al., 2014; Zegeye et al., 2014; Voss-Fels et al., 2015; Riaz et al., 2018). The number of significant marker pairs was found lowest in the chromosome 4D similar to a previous study (Muleta et al., 2017a).

Population structure in the panel showed two subpopulations (Kumar et al., 2020) which was further supported by PCA. A significant effect of population stratification was observed in differences based on disease response at seedling and adult plant stage. The resistant subpopulation of the two subpopulations, comprised of majorly improved genotypes and released varieties. It appeared that landraces in the minor subpopulation were found mostly susceptible. The landraces under study have originated from central and northwestern states of India namely Gujarat, Rajasthan, and Madhya Pradesh (Kumar et al., 2020). Landraces adapted to these regions are mostly tolerant to abiotic stresses *viz.* drought, heat, and salinity, e.g., genotype KHARCHIA LOCAL (origin: Rajasthan, India) is adapted to long-term salinity stress (Mahajan et al., 2017, 2020). We hypothesize that landraces adapted to harsh environments, such as abiotic stresses and low-input conditions give an eluded appearance of diseases. But under high input conditions, we observed that landraces appeared to be more susceptible when compared to improved genotypes. Since not all landraces were found susceptible (Supplementary Table S3), they may still be beneficial in the identification of novel resistant alleles. Of 96 landraces in the panel, twenty-one were found resistant in field response against LR and eleven each against YR and SR. Landrace IC321998 (origin: Uttarakhand, India) was found resistant for all three rusts. This suggests an indirect selection for disease resistance in spite of the fact that landraces are traditionally selected by the farmers preferring better agronomic traits (Zeven, 2002).

Care was taken to avoid spurious association due to population stratification by treating it as a covariate along with kinship accounting relationship among genotypes for association studies in this panel (Stich et al., 2008; Lipka et al., 2012; Yang et al., 2014). The selection of CMLM applied in this study was found suitable from the comparative study of different association models. In most of the cases, based on the Q-Q plots, CMLM was found to have minimum deviations in observed probabilities with respect to expected probabilities (Supplementary File S1). Several studies have been successfully conducted using the CMLM model (Arruda et al., 2016; Muleta et al., 2017a, b; Sheoran et al., 2019). It also provides efficiently high statistical power over and is an improvement to the MLM model for a large sample size (Zhang et al., 2010).

Three ASR loci *QYr.ramp-1A.2*, *QLr.ramp-1A.2*, and *QSr.ramp-1A.2* were found co-localized on chromosome 1A against each rust located at 72.25–75.74 cM. Except for *QYr.ramp-1A.2* exhibiting minor effect, other loci were found associated with disease response to multiple pathotypes and environments. This was in agreement with the QTL observed to have a minor effect but also showed seedling resistance on chromosome 1AL (Rosewarne et al., 2012; Jighly et al., 2015). *QLr.umn-1AL* was reported to be associated with both field and seedling disease response mapped at 149.8 cM on chromosome 1AL (Gao et al., 2016). In this study, *QLr.ramp-1A.2* was observed proximal to this previously reported QTL. APR for LR is not known to be present on chromosome 1AL (Li et al., 2014) and the chance of having an association with *Lr59* is highly unlikely as source of such alien introgression (Marais et al., 2008) is not present in the current study panel. A MTA exhibiting seedling resistance against *Pgt* race TTKSK represented by DArT marker *wPt-*2014 was reported on chromosome 1AL mapped at 70.5 cM (Laidò et al., 2015). This could be present in close proximity to the loci *QSr.ramp-1A.2* identified in this study.

LR APR loci *QLr.ramp-1B.2* was mapped on the short arm of chromosome 1B located at 8.24–9.93 cM. It was identified in close proximity to SSR marker *Xswm271* linked to known APR gene *Lr75* (Singla et al., 2017) and proximal to another APR gene *Lr71* (Singh et al., 2013). Genomic region 23.90–26.22 cM (1B) was found to have a large number of MTAs in all the three rusts for seedling disease response (Figure 5 and Table 3). This region located on chromosome 1BL harbors several known *Yr* genes such as *Yr9, Yr10, Yr15, Yr 64, Yr65*, etc. In concurrence ASR loci *QYr.ramp-1B.3* was found in a close proximity to *Xbarc119* (27.4 cM) and *Xgwm413* (29.9 cM) which are linked to *Yr15* and *YrH52* (Maccaferri et al., 2015a). This region also showed a possible association with wheat-alien translocation (1RS.1BL) derived from *Secale cereale* (Schlegel and Korzun, 1997). Resistance genes for all the three rusts are known to be associated with this translocation *viz. Yr9* (Ren et al., 2009), *Lr26*, and *Sr31* (Mago et al., 2005). Race-specific gene postulations demonstrated the presence of these genes in the panel, possibly being reflected as the co-localized ASR QTL *QYr.ramp-1B.3*, *QLr.ramp-1B.3*, and *QSr.ramp-1B.4*. The most virulent *Pgt* race Ug99 and its lineage have not been observed in India and its neighboring countries inferring the effectiveness of *Sr31* in Indian germplasm (Prasad et al., 2018; Bhardwaj et al., 2019b).

So far, five QTL have been reported for resistance against YR on chromosome 1D, with three present at the distal end (Zwart et al., 2010; Ren et al., 2012a; Vazquez et al., 2012) and two present at the proximal end of its short arm (Maccaferri et al., 2015b; Godoy et al., 2018). Not many reports are present for ASR genes against YR at locations close to the centromere on chromosome 1DS where Godoy et al. (2018) reported QTL present at 89.58 cM. Two potential ASR loci *QYr.ramp-1D.1* (AX-94508418: 1.03 Mb) and *QYr.ramp-1D.3* (AX-94444583: 34.6 Mb) were observed both at distal and proximal regions of 1DS, respectively, in this study. The chromosome 1DS is also known to harbor *Lr42* gene conferring resistance against LR at both seedling and adult plant stages and recently it has been fine mapped on 1DS flanked by markers *TC387992* and *Xwmc432* to a 3.7 cM genetic interval (Gill et al., 2019). *QLr.ramp-1D* (3.38 cM) was observed very close to this location at adult plant stage only. *QSr.ramp-1D.1* located at 1.69 cM on 1DS was observed in close proximity of gene *Sr33* which is known to be flanked by *Xbarc152* and *Xcfd15* at a distance of 1.8 cM on either end on 1DS (Sambasivam et al., 2008). The gene was introgressed in bread wheat from its wild relative *Aegilopes taushii* providing resistance against Ug99 group races (Periyannan et al., 2013).

The genomic region 102.12–104.59 cM was observed to have ASR loci for each rust. This region was present on the chromosome 2BL supported by the physical positions of QTL associated SNPs spanning ∼612–722 Mb region. Corresponding to this physical region, Aoun et al. (2019) identified *QSr.ace-2B* conferring resistance to *Pgt* races TTKSK and JRCQC (virulent to *Sr9e*) linked to *Sr9* gene flanked by SNP markers *IWA6399* and *IWA7955* (92.7–105.3 cM) on chromosome 2BL. SR ASR loci *QSr.ramp-2B.6* was identified within this genomic region supporting possible affiliation to *Sr9*. In co-localization to which YR ASR loci *QYr.ramp-2B.4* was identified in a single field environment possibly due to its minor effect. It was located 5 cM proximal to another minor effect QTL *QYrAvS.wgp-2BL* (Liu et al., 2020). It was further located 3 cM proximal to SNP marker *IWA638* (724 Mb: 107.58 cM) (Blake et al., 2016) which has been identified in a close proximity of *YrSp* gene (Feng et al., 2015). Another ASR loci *QLr.ramp-2B.7* against LR was identified at 103.81–104.59 cM on chromosome 2BL. Gao et al. (2016) reported a QTL *2B_*3 at 102.28–108.35 cM interval on chromosome 2B in response to field experiments only which reduced any possible similarity. Two formally known LR resistance genes *Lr50* and *Lr58* are present on the terminal region of chromosome 2BL (Brown-Guedira et al., 2003; Kuraparthy et al., 2007). SSR markers *Xgdm87* (*Lr50*) and *Xcfd50 (Lr58*) linked to these genes are present distant to the identified QTL *QLr.ramp-2B.7* based on the integrated consensus map (Maccaferri et al., 2015a). In a previous report, multiple resistance QTL against YR and LR *QYr.ifa-2BL/QLr.ifa-2BL* was identified on chromosome 2BL. It was flanked by DArT markers *wPt-3378* and *wPt-7360* (Crossa et al., 2007; Buerstmayr et al., 2014). However these markers were also present distant (Maccaferri et al., 2015a) to *QLr.ramp-2B.7*, leaving it for being an unexplored ASR loci.

In our study, it was observed that the genomic region 174.29–178.60 cM on chromosome 3DL harbored the most number of MTAs for seedling disease response against LR. Pathotypes LR_12-5, LR_104-2, LR_77-9, LR_77-5, and LR_77-1 collectively contributed to these MTAs observed (Figure 5 and Table 3). This region was also found highlighted in seedling disease response against SR pathotypes SR_122, SR_117-6, SR_40A, and SR_11. Wheat-alien translocations were known to be carried out for incorporating resistance genes *Lr24/Sr24* (T3DS.3DL-3Ae#1L) *and Lr38* (T3DL.3DS-7Ai#2L) in the chromosome 3D derived from *Agropyron elongatum* and *Agropyron intermedium*, respectively (Friebe et al., 1993; Jiang et al., 1993). ASR loci *QLr.ramp-3D.6* and *QSr.ramp-3D.4* were found associated with these translocations possibly harboring these resistance genes in our panel as evident by race-specific genes postulated.

Two APR and one ASR loci were identified co-localized on chromosome 6AL located at 215.37-221.75 cM (∼605–615 Mb). *QYr.ramp-6A.5* was the ASR loci which provided resistance against YR in this region. It has been found distal to a previously reported ASR QTL tagging SNP *IWA2129* on chromosome 6AL mapped at 212.2 cM (596.89 Mb) (Blake et al., 2016; Muleta et al., 2017a). Chromosome 6AL is also known to harbor the YR ASR gene *Yr38* which is linked with another ASR gene *Lr56* for LR (Park, 2016). None of these genes were postulated in the current study panel. In a previous study, *Lr64* gene was mapped on chromosome 6AL tightly linked to the KASP marker which was developed for the SNP marker *IWB59855* (Kolmer et al., 2019). The LR APR QTL *QLr.ramp-6A.4* identified was represented by SNP marker AX-95147877 (615.46 Mb) in this study, found to be present close to SNP marker *IWB59855* (614.17 Mb). Therefore, *QLr.ramp-6A.4* could be strongly associated with the gene *Lr64*. Interestingly, *Lr64* has not been characterized as ASR or APR gene so far (Park, 2016), whereas *QLr.ramp-6A.4* was identified exhibiting APR response. This could provide supporting evidence for the identification of the true resistance nature of *Lr64* in the future. An ASR QTL *QSr.fcu-6A* was mapped earlier on chromosome 6AL to the genomic region harboring *Sr13* gene conferring resistance against *Pgt* races TMLKC, TRTTK, and TTKSK in emmer wheat (Sharma et al., 2019). An SNP-based semi-thermal asymmetric reverse PCR (STARP) marker *rwgsnp7* was developed from the SNP *IWB34398* (6AL: 615.45 Mb) to map *QSr.fcu-6A*. Validation of marker *rwgsnp7* in hexaploid wheat provided evidence that chromosome 6D harbors a homoeologous allele similar to that present on chromosome 6A for this marker (Sharma et al., 2019). In this study, *QSr.ramp****-****6A.5* was identified as APR loci mapped at 215.37–221.75 cM (∼605–615 Mb) interval on chromosome 6AL where the SNP *IWB34398* overlaps with this region. Therefore, *QSr.ramp-6A.5* could be present in the genomic region harboring *Sr13*. Since none of the *Pgt* races mentioned above were used in this study, seedling resistance could not be confirmed governed by this region in this study.

Chromosome 6B was observed to have co-localized APR loci for the three rusts. Based on MTAs associated with these QTL, an estimated physical location of ∼712–714 Mb was obtained (IWGSC RefSeq v.1.0) representing the long arm of the chromosome. Among several other reported YR APR QTL on 6BL (Mu et al., 2019), three QTL *viz. QYr.wsu-6B.1* (Bulli et al., 2016), *QYr.ucw-6B* (Maccaferri et al., 2015b), and *QYr.wgp-6B.1* (Santra et al., 2008) spanning ∼631–720 Mb genomic region were found overlapping to some extent with *QYr.ramp-6B.4* identified in this study. LR APR QTL *QLr.ramp-6B.6* was identified distally close to a recently reported APR QTL *QLr.cim-6BL* mapped in a durum mapping population on chromosome 6BL (Li et al., 2020). It was located in the interval of SNP markers AX-95155193 and AX-94562707 spanning 691.07–698.26 Mb genomic region. *Lr3* (Kolmer, 2015) and *Lr9* (Schachermayr et al., 1994) are well known genes present on chromosome 6BL. *QLr.ramp-6B.6* was unlikely to be linked with either of these genes as *Lr3* is an ASR gene and *Lr9* has not been deployed in the current study panel. Similarly, *Sr11* gene is also present on chromosome 6BL but exhibits ASR (origin: *Triticum turgidum* ssp. *durum*) (Park, 2016). It was mapped within the marker interval of *Tdurum_contig55744_822* (IWB72471: 709.53 Mb) and *BS00074288_51* (IWB10724: 715.97 Mb) (Blake et al., 2016; Nirmala et al., 2016). This region was found to be overlapping with those of QTL *QSr.ramp-6B.3*. Several genotypes were postulated to have this gene in the study panel. Since *Sr11* provides a race-specific ASR against *Pgt* race TKTTF which has not been used in this study, *QSr.ramp-6B.3* could have been mistakenly identified as an APR loci. Although, without further investigation a conclusion cannot be made.

The region (17.65–18.61 cM) harboring one ASR loci against YR and SR each, and one APR loci against LR were identified on the long arm of chromosome 6D (∼459–460 Mb). Gebrewahid et al. (2020) reported an APR QTL *QYr.hebau-6DL* located at 464.6–472.0 Mb interval flanked by SNP markers AX-108848475 and AX-109273869. QTL *QYr.ramp-6D.2* identified in this study was present proximal to *QYr.hebau-6DL*, showing ASR property. There was a clear difference in the nature and position of these two QTL. LR APR QTL *QLr.ramp-6D.1* was observed in a single environment depicting a possible minor effect. Although, chromosome 6DL harbors a known ASR gene *Lr38* inheritably linked with SSR marker *Xwmc773* (Mebrate et al., 2008) could be present relatively close to *QLr.ramp-6D.1.* Of the four known *Sr* genes on chromosome 6D, only the ASR gene *Sr29* is present on the long arm (Dyck and Kerber, 1977). In a report, an APR QTL *QSr.cim-6DL* was mapped on chromosome 6DL. In our study, *QSr.ramp-6D* was identified as an ASR loci which could be in close proximity of *Sr29*.

The telomeric region of chromosomes 2AL and 7DS bears significant importance in YR resistance along with other resistant systems for barley yellow dwarf virus, powdery mildew and fusarium head blight (Boukhatem et al., 2002). Apart from the three known YR seedling resistance genes *viz. Yr1* (Bansal et al., 2009), *Yr32* (Eriksen et al., 2004), and *YrJ22* (Chen et al., 2016), a recently identified and temporarily designated YR seedling resistance gene *YrH9017* (Dong-fang et al., 2019) has been found on chromosome 2AL. None of the genotypes in the panel harbored these genes based on their pedigree and race-specific gene postulations. Instead, an unexplored YR APR loci *QYr.ramp-2A.4* (179.61 cM) was observed at the telomeric region represented by SNP AX-94463225 (764.1 Mb). In co-localization to which, a LR APR loci *QLr.ramp-2A.5* (AX-94995671: 779.6 Mb) was observed 33.07 cM and 73.07 cM distal to APR QTL *QLr.ubo-2A* (Li et al., 2014) and *QLr.hebau-2AL* (Zhang et al., 2017), respectively, in this study. APR loci *QYr.ramp-7D.2* and *QLr.ramp-7D.2* on chromosome 7D (6.96-18.37 cM) were found associated possibly with pleiotropic resistance gene *Lr34/Yr18/Sr57*, where SNPs representing these QTL *viz.* AX-95205886 (5.38 Mb) and AX-94674448 (72.94 Mb), respectively being in high LD (D’ = 0.87) are present on the short arm of chromosome 7D based on the IWGSC RefSeq v1.0 (Appels et al., 2018) reference physical map. The association to this pleiotropic gene can be further supported by the fact that forty genotypes in the current study panel were characterized for *Lr34* in a previous report (Pawar et al., 2013). When compared to 90K SNP array, 35K SNP array used in this study was able to capture association with this pleiotropic resistance gene, since none of the 90K SNP markers are known to be linked to this gene (Muleta et al., 2017b). Since this array is capable of capturing conserved regions associated with yield-related traits present on the 1RS.1BL translocation region (Luján Basile et al., 2019) and was designed using wild relatives (Allen et al., 2017), provided an advantage over the most utilized 90K SNP array.

Evidence of true QTL can be derived from the results of having significant associations seen in at least two pathotypes or environments for a rust type (Edae et al., 2018). Validation of markers associated with these QTL can be done by comparing them with the positions of the markers of previously mapped QTL (Pasam et al., 2012; Hwang et al., 2014; Kertho et al., 2015; Maccaferri et al., 2015a). In this study, MTAs found associated with multiple pathotypes may be broadly useful in the enhancement of future germplasm and breeding programs (Liu et al., 2017). Accumulation and detection of favorable resistance alleles often pose challenges in the breeding program upon consideration (Riaz et al., 2018). In our study, a large number of favorable resistant alleles were detected in some landraces and these showed moderate to resistant behavior against the three rusts. As a separate observation, SNP AX-94394356, AX-94674448, and AX-95176310 being the QTL representing markers in field response against LR, had a poor percentage of resistance alleles accumulated in improved genotypes and released varieties when compared to landraces, in the panel. Similarly, AX-94608698, AX-95226309, AX-94485764, and AX-94805944 observed for SR field response, had more accumulated favorable alleles in landraces than that in other genotype categories. Such observations were not distinctively visible in case of YR response. This observation is possible when these favorable alleles have accumulated in landraces over time with varying frequencies (Riaz et al., 2018). The concept of ‘Speed breeding’ introduced (Watson et al., 2018) with the aim of utilizing latest advancements in phenotyping and high throughput data generation, can be helpful in developing rust-resistant wheat cultivars at an accelerated rate (Hickey et al., 2012; Riaz et al., 2016). To determine the identified QTL to be potentially novel or are associated with previously mapped QTL, allelism tests will be required.

Breeders engaged in wheat genetic improvement have a keen interest in looking for desirable parents or donor lines to enrich their crossing block. While grain yield is prime focus area along with end product making quality parameters, yet stabilization of yield potential through tolerance against abiotic and biotic stresses will always be a major concern. Therefore, any information on diverse or novel sources of resistance, in this case against the three rusts, is of considerable importance to the breeders. Since wheat cultivation in India corresponds to many of the agro-climatic regions growing wheat globally, the information becomes universally important in combating rust diseases. Our finding of hitherto less utilized sources of stripe, leaf and stem rust *viz.* IC321918, IC212153AMB, IC321998, H957, LBRL4, and some exotic lines shall attract the attention of the wheat breeders. When present alone, the APR genes/QTL do not confer adequate resistance especially under high disease pressure; however, combinations of 4 or 5 minor genes usually result in “near-immunity” or a high level of resistance (Singh et al., 2014). Increasing the frequency of QTL for rust resistance through divergent crossing in the population improvement program of wheat can provide durable rust resistance by broadening the genetic base for resistance. The pleiotropic QTL can confer slow rusting resistance against the three rusts of wheat. Bi-parental and multi-parent populations developed using resistant landraces and modern varieties shall enable toward this endeavor.

## Data Availability Statement

All datasets generated for this study are included in the article/Supplementary Material.

## Author Contributions

RT, AR, and DiK conceived the theme of study. DeK, AK, SS, SJ, MI, and UA analyzed the data. DeK, VC, and RT drafted the manuscript. DeK conducted the field condition phenotyping at Karnal, India. OG and SB conducted the phenotyping of the seedling resistance test. MS conducted the field condition phenotyping at Wellington, India. SP and TP the conducted field condition phenotyping at Indore, India. HK, RS, and PS provided insights into reported genes for rust resistance. VC, RT, GS, AR, GPS, and DiK provided overall guidance and edited the manuscript. All authors read and approved the final version of the manuscript.

## Conflict of Interest

The authors declare that the research was conducted in the absence of any commercial or financial relationships that could be construed as a potential conflict of interest.

## Footnotes

1 https://github.com/YinLiLin/R-CMplot

2 http://plants.ensembl.org/Triticum_aestivum/Info/Index

3 https://blast.ncbi.nlm.nih.gov/Blast.cgi

## Supplementary Material

FIGURE S1 Heatmap of correlation coefficients showing the correlation between the 18 pathotypes representing the three rusts used in the study. The magnitude of the correlation is represented by the color gradient legend on the right side of the heatmap. Significant correlations are marked as ^∗^(*p* < 0.05), ^∗∗^(*p* < 0.01), and ^∗∗∗^(*p* < 0.001).

FIGURE S2 GGE Biplot representing variations explained in the first two principal components for field disease response against YR, LR, and SR. Concentric circles represent the ranking of genotypes with respect to being an ideal genotype stable in corresponding environments.

FIGURE S3 Population stratification study by using principal component analysis on 483 genotypes in the panel with variation partitioned between the first two principal components. **(A)** Two subpopulations observed in STRUCTURE analysis are represented with separate colors in the PCA plot. Also, **(B)** different categories of genotypes in the panel are represented in different colors.

FIGURE S4 **(A)** Q-Q plot, **(B)** Manhattan plot, and **(C)** minor allele frequency of the MTAs observed for seedling stage disease response against 18 pathotypes representing the three rusts using CMLM.

FIGURE S5 **(A)** Q-Q plot, **(B)** Manhattan plot, and **(C)** minor allele frequency of the MTAs observed for adult plant field disease response against the three rusts in different environments using CMLM.

FIGURE S6 Haplotype analysis of the representative SNPs observed for putative QTL in the study for **(A)** stripe rust, **(B)** leaf rust, and **(C)** stem rust field responses. The number and variations in the grayscale represent the percentage magnitude of LD *r*^2^-value between marker pairs.

FIGURE S7 Hierarchical clustering based dendrogram representing 72 genotypes resistant to multiple rusts. The numbers suffixed to genotypes in parenthesis represents the total number of favorable alleles observed for field response based QTL representative SNPs.

TABLE S1 Avirulence/virulence formula of predominant Indian wheat rust pathotypes used in the present study.

TABLE S2 Nomenclature of environments (12) considered for the evaluation of field disease response at adult plant stage.

TABLE S3 Phenotypic evaluation of RAMP at seedling and adult plant stage for multiple rusts.

TABLE S4 Basic statistics of disease response on RAMP at seedling and adult plant stages.

TABLE S5 Pearson’s Correlation matrix of seedling resistance test (SRT) for stripe (5), stem (7), and Leaf (6) rust pathotypes.

TABLE S6 Correlation analysis of phenotypic data for multiple rusts observed in different environments (*p*-value ≤ 0.001^∗∗∗^, 0.01^∗∗^, 0.05^∗^).

TABLE S7 Phenotypic reactions of the 483 genotypes included in the study to multiple rusts at seedling and adult stage, grouped based on population structure.

TABLE S8 Marker trait associations (MTAs) observed at seedling stage for Rust pathotypes in RAMP at −log_10_*P* ≥ 3.

TABLE S9 Putative QTLs identified significantly associated with seedling stage disease response against multiple rust pathotypes.

TABLE S10 Marker trait associations (MTAs) observed at adult plant stage for multiple rusts in RAMP at −log_10_*P* ≥ 3.

TABLE S11 Putative QTLs identified significantly associated with adult plant stage disease response against the three rusts.

TABLE S12 Putative QTL observed at seedling and adult plant stage for the three rusts.

TABLE S13 Distribution of favorable alleles of representative SNPs in 483 wheat genotypes.

FILE S1 Comparative Q–Q plots of six association models for multiple rust pathotypes and four environments each for YR, LR, and SR. The CMLM was observed as the best fit model.
