# Supplementary figures and images for: Genome-Wide Association Studies in Diverse Spring Wheat Panel for Stripe, Stem, and Leaf Rust Resistance
Source: Front Plant Sci. 2020 Jun 3;11:748. doi: 10.3389/fpls.2020.00748 (PMC7286347; doi:10.3389/fpls.2020.00748)

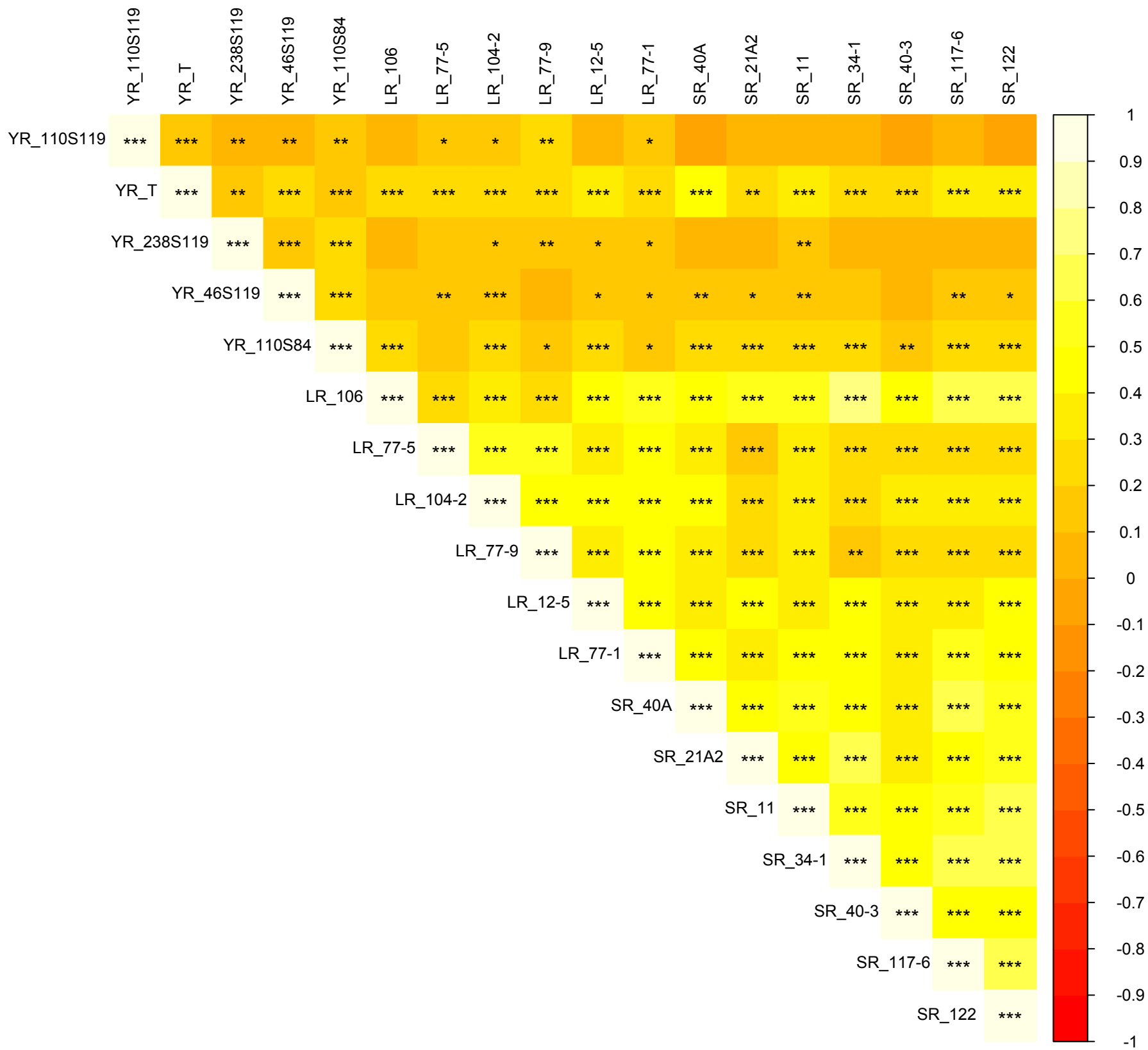

Supplement: FIGURE S1 — Heatmap of correlation coefficients showing the correlation between the 18 pathotypes representing the three rusts used in the study. The magnitude of the correlation is represented by the color gradient legend on the right side of the heatmap. Significant correlations are marked as ∗(p < 0.05), ∗∗(p < 0.01), and ∗∗∗(p < 0.001). [file Image_1.pdf]

# Ranking genotypes for YR response

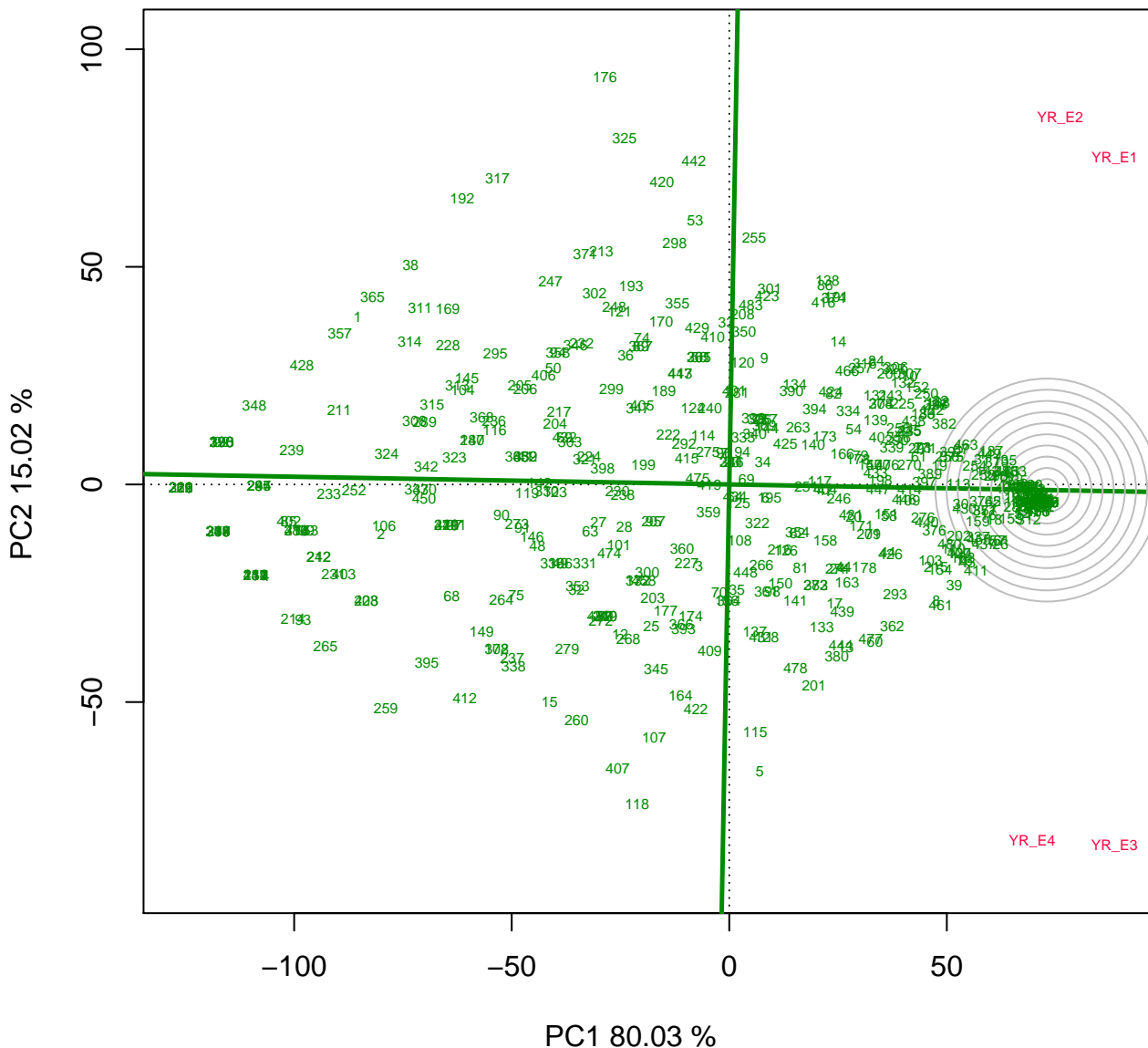



# Ranking genotypes for SR response

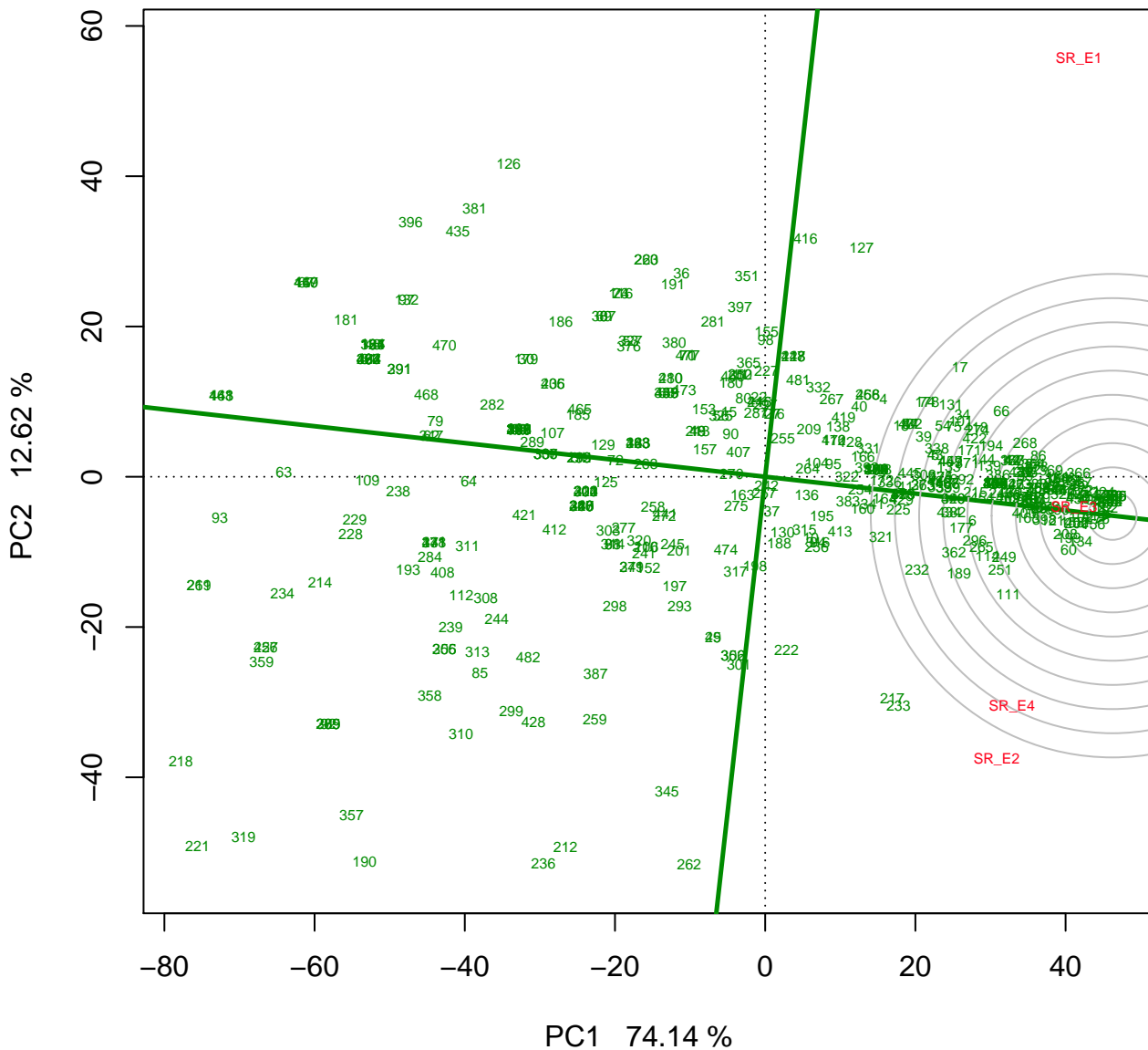

Supplement: FIGURE S2 — GGE Biplot representing variations explained in the first two principal components for field disease response against YR, LR, and SR. Concentric circles represent the ranking of genotypes with respect to being an ideal genotype stable in corresponding environments. [file Image_2.pdf]

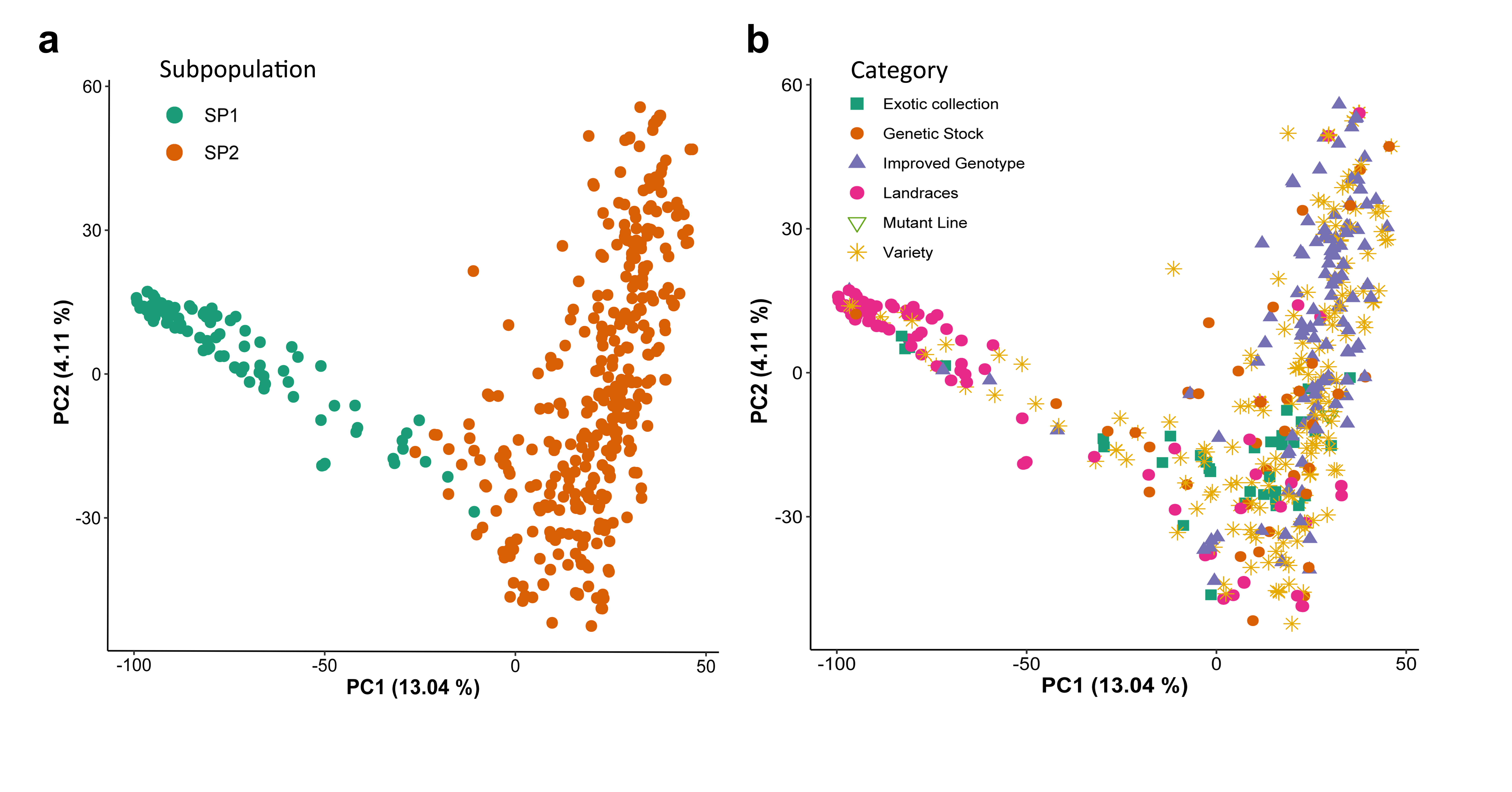

Supplement: FIGURE S3 — Population stratification study by using principal component analysis on 483 genotypes in the panel with variation partitioned between the first two principal components. (A) Two subpopulations observed in STRUCTURE analysis are represented with separate colors in the PCA plot. Also, (B) different categories of genotypes in the panel are represented in different colors. [file Image_1.TIF]

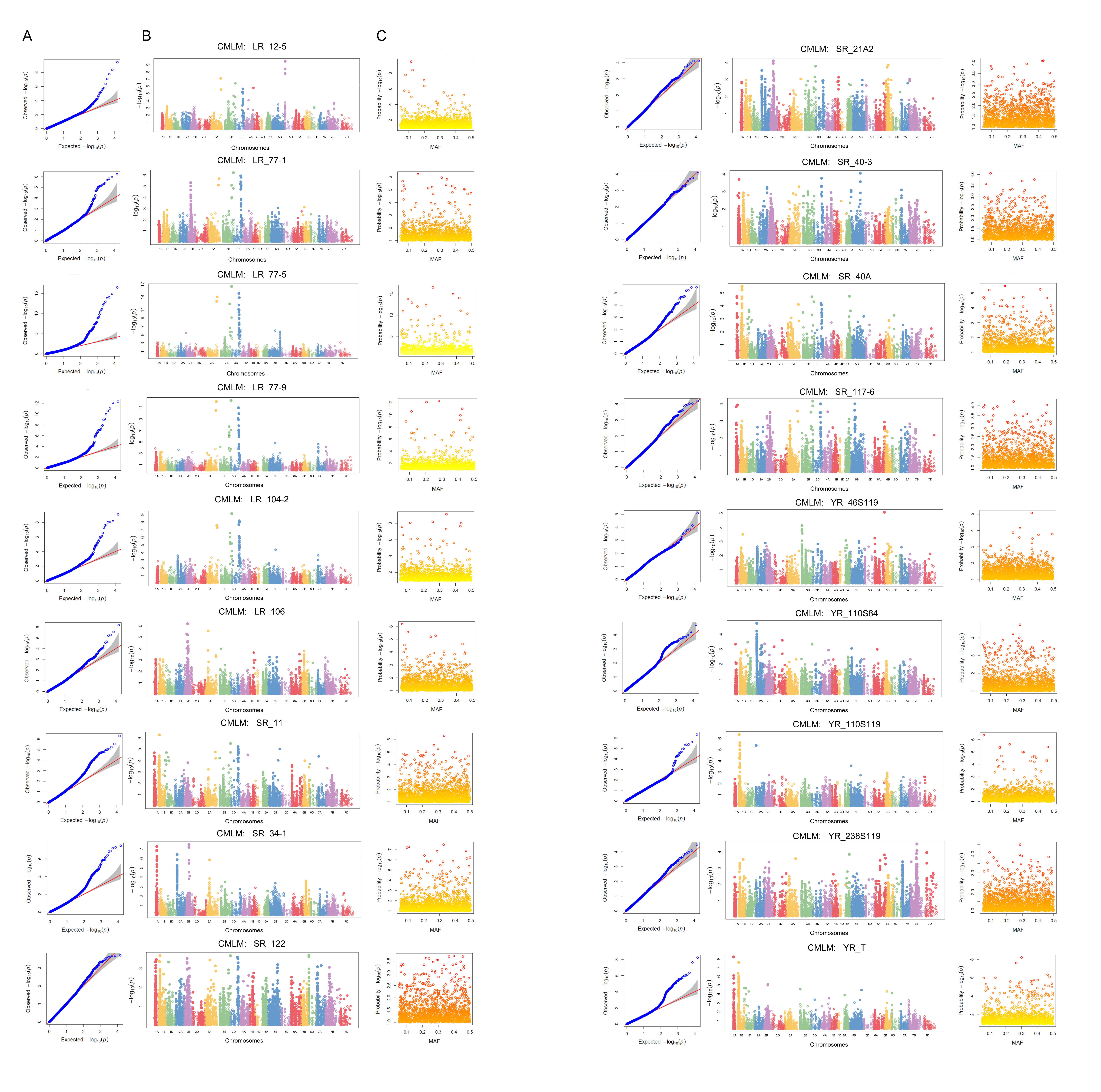

Supplement: FIGURE S4 — (A) Q-Q plot, (B) Manhattan plot, and (C) minor allele frequency of the MTAs observed for seedling stage disease response against 18 pathotypes representing the three rusts using CMLM. [file Image_4.tif]

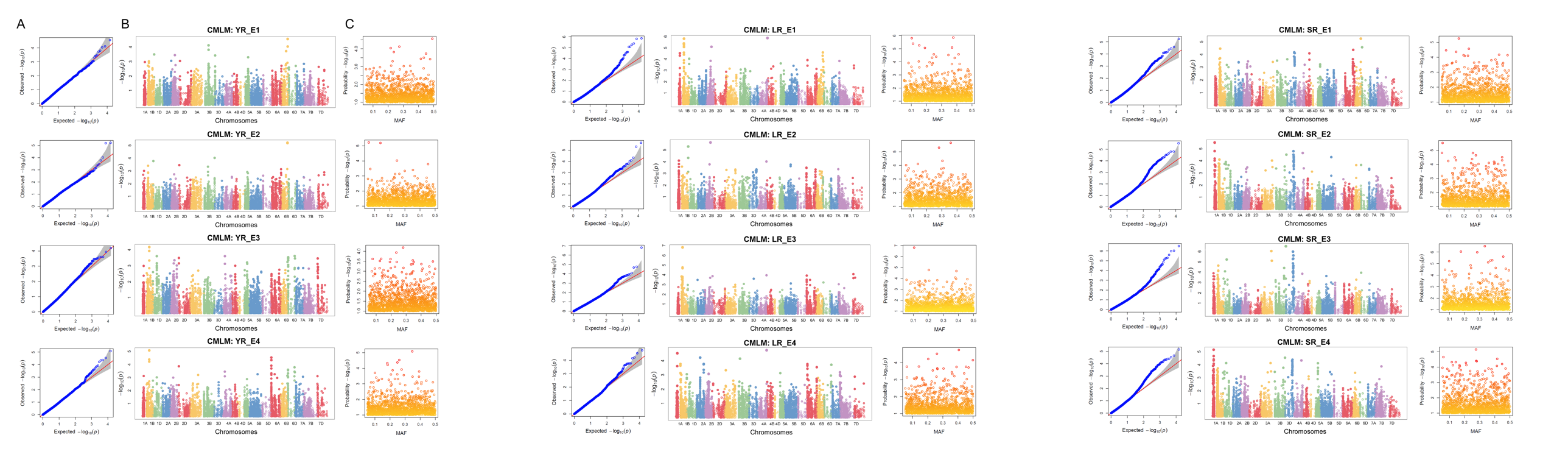

Supplement: FIGURE S5 — (A) Q-Q plot, (B) Manhattan plot, and (C) minor allele frequency of the MTAs observed for adult plant field disease response against the three rusts in different environments using CMLM. [file Image_5.tif]

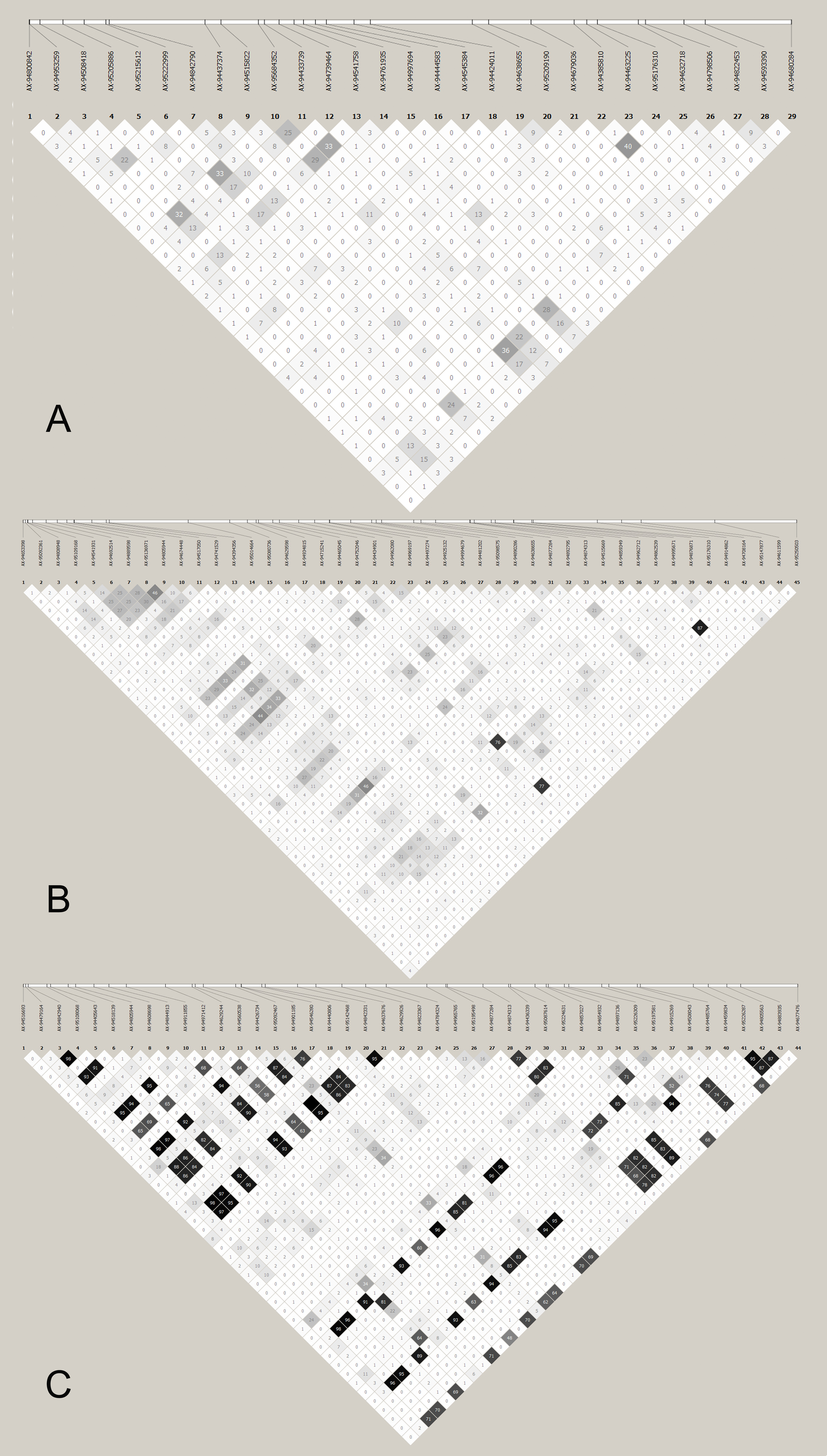

Supplement: FIGURE S6 — Haplotype analysis of the representative SNPs observed for putative QTL in the study for (A) stripe rust, (B) leaf rust, and (C) stem rust field responses. The number and variations in the grayscale represent the percentage magnitude of LD r2-value between marker pairs. [file Image_6.tif]

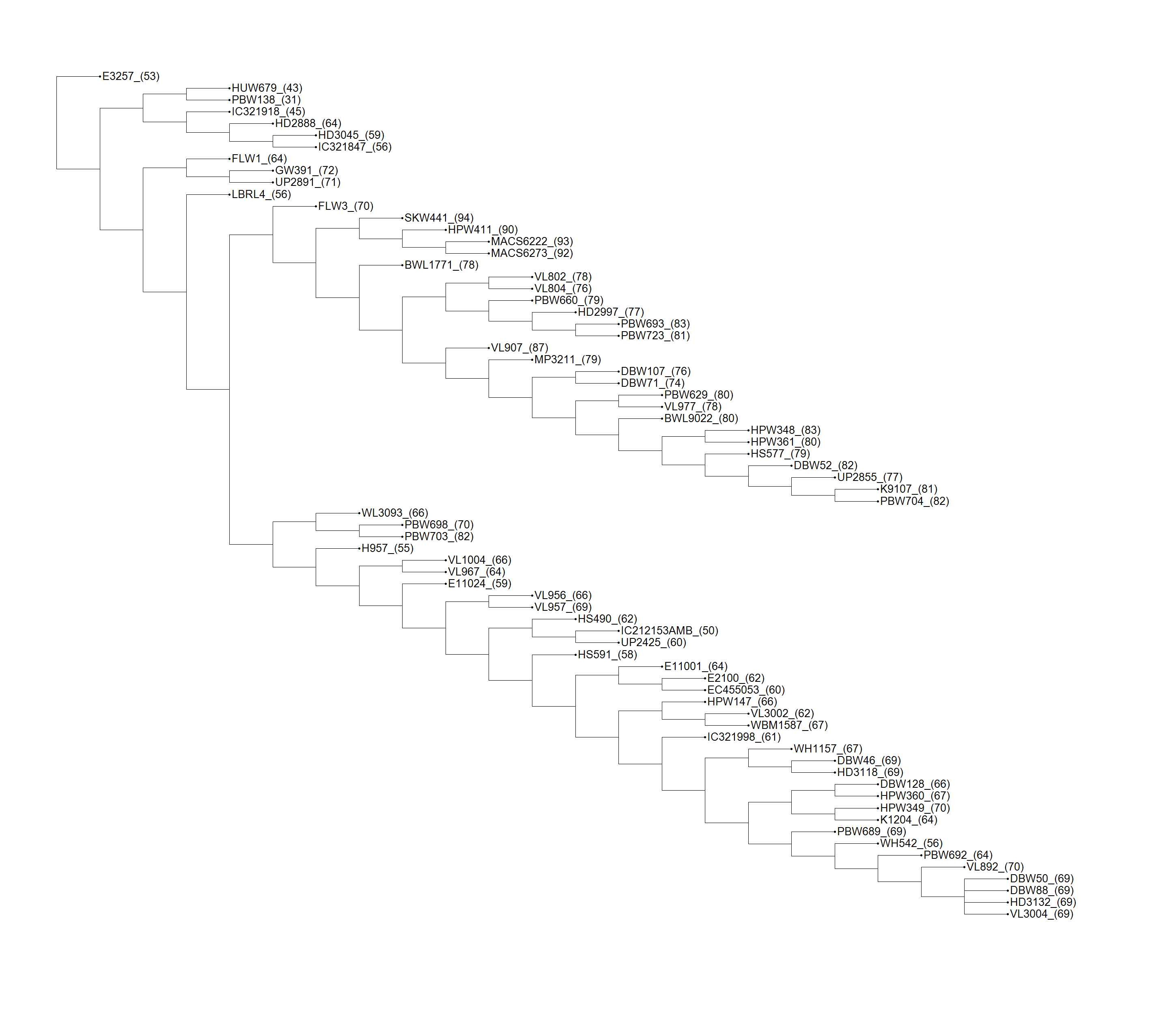

Supplement: FIGURE S7 — Hierarchical clustering based dendrogram representing 72 genotypes resistant to multiple rusts. The numbers suffixed to genotypes in parenthesis represents the total number of favorable alleles observed for field response based QTL representative SNPs. [file Image_7.tif]

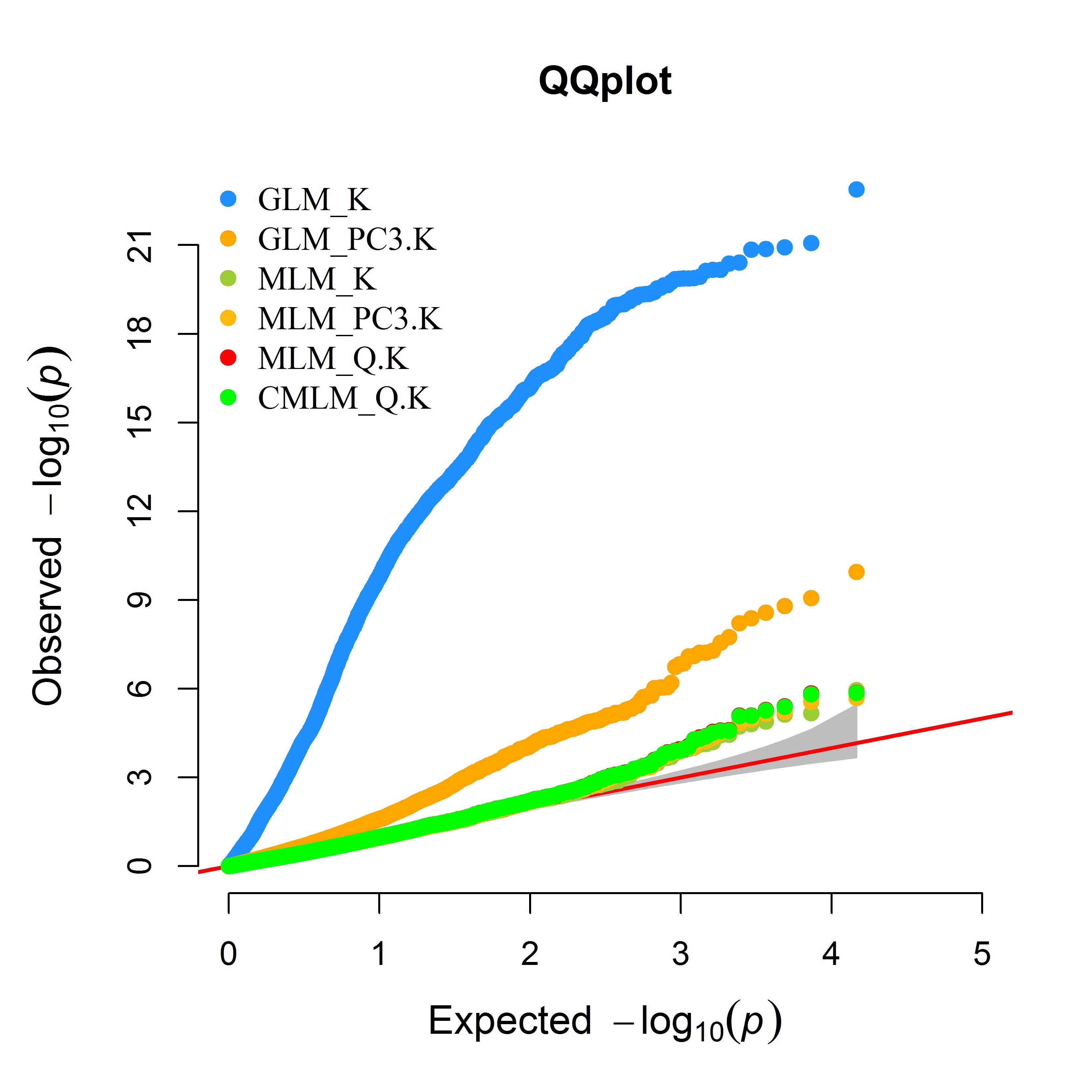

Supplement: FILE S1 — Comparative Q–Q plots of six association models for multiple rust pathotypes and four environments each for YR, LR, and SR. The CMLM was observed as the best fit model. [file Data_Sheet_1.ZIP › Q_Q Plots-Model Comparison/Adult plant stage/LR_E1.jpg]

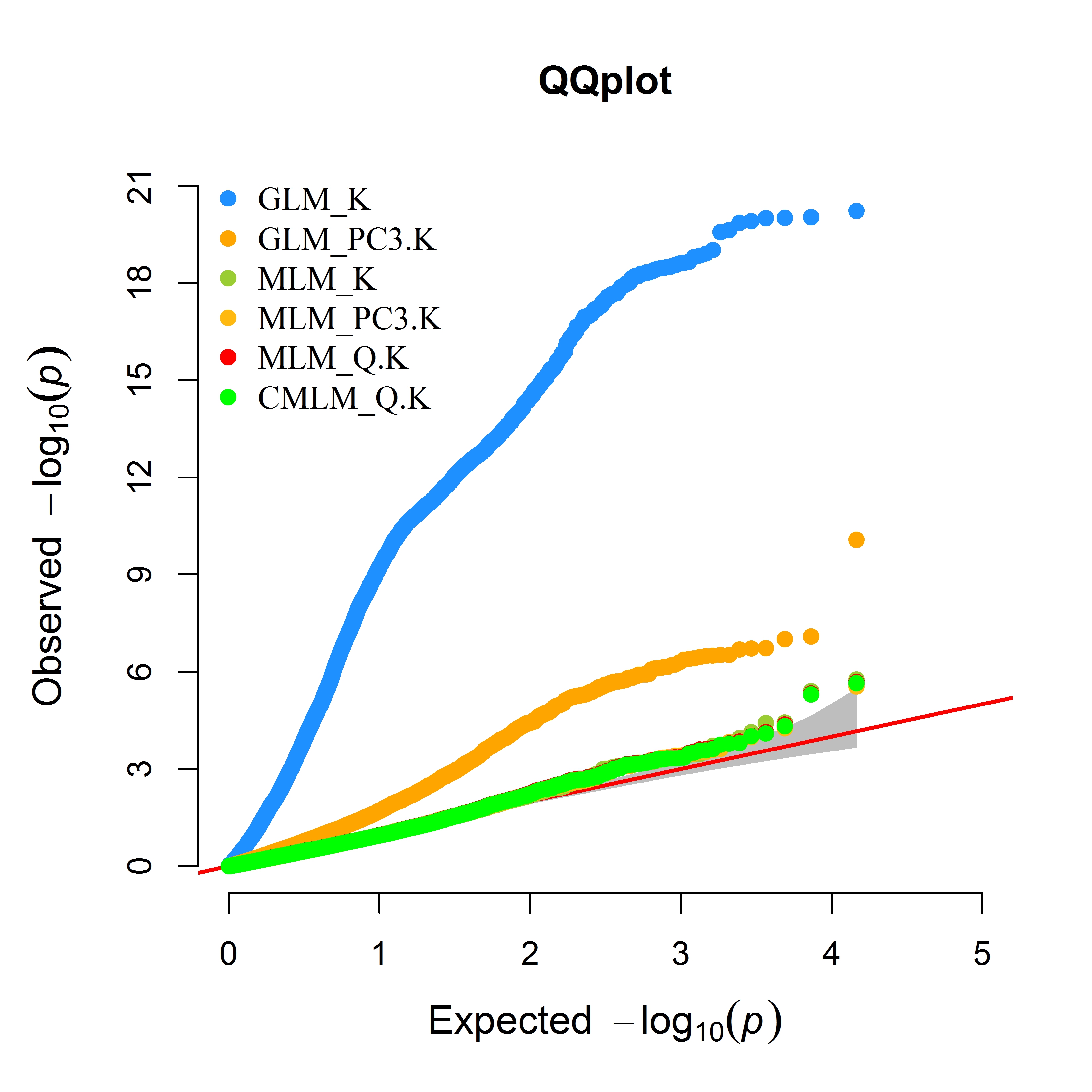

Supplement: FILE S1 — Comparative Q–Q plots of six association models for multiple rust pathotypes and four environments each for YR, LR, and SR. The CMLM was observed as the best fit model. [file Data_Sheet_1.ZIP › Q_Q Plots-Model Comparison/Adult plant stage/LR_E2.jpg]

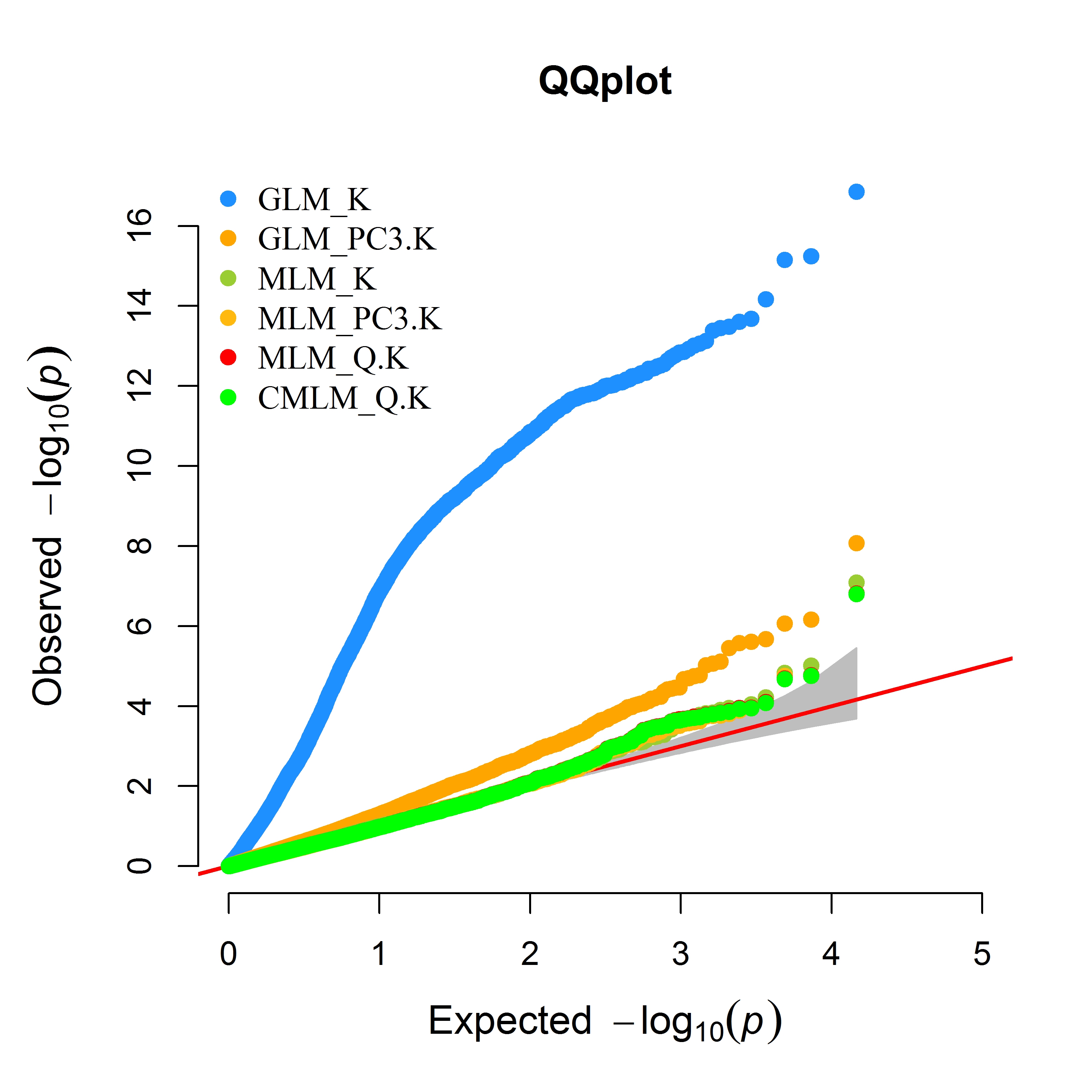

Supplement: FILE S1 — Comparative Q–Q plots of six association models for multiple rust pathotypes and four environments each for YR, LR, and SR. The CMLM was observed as the best fit model. [file Data_Sheet_1.ZIP › Q_Q Plots-Model Comparison/Adult plant stage/LR_E3.jpg]

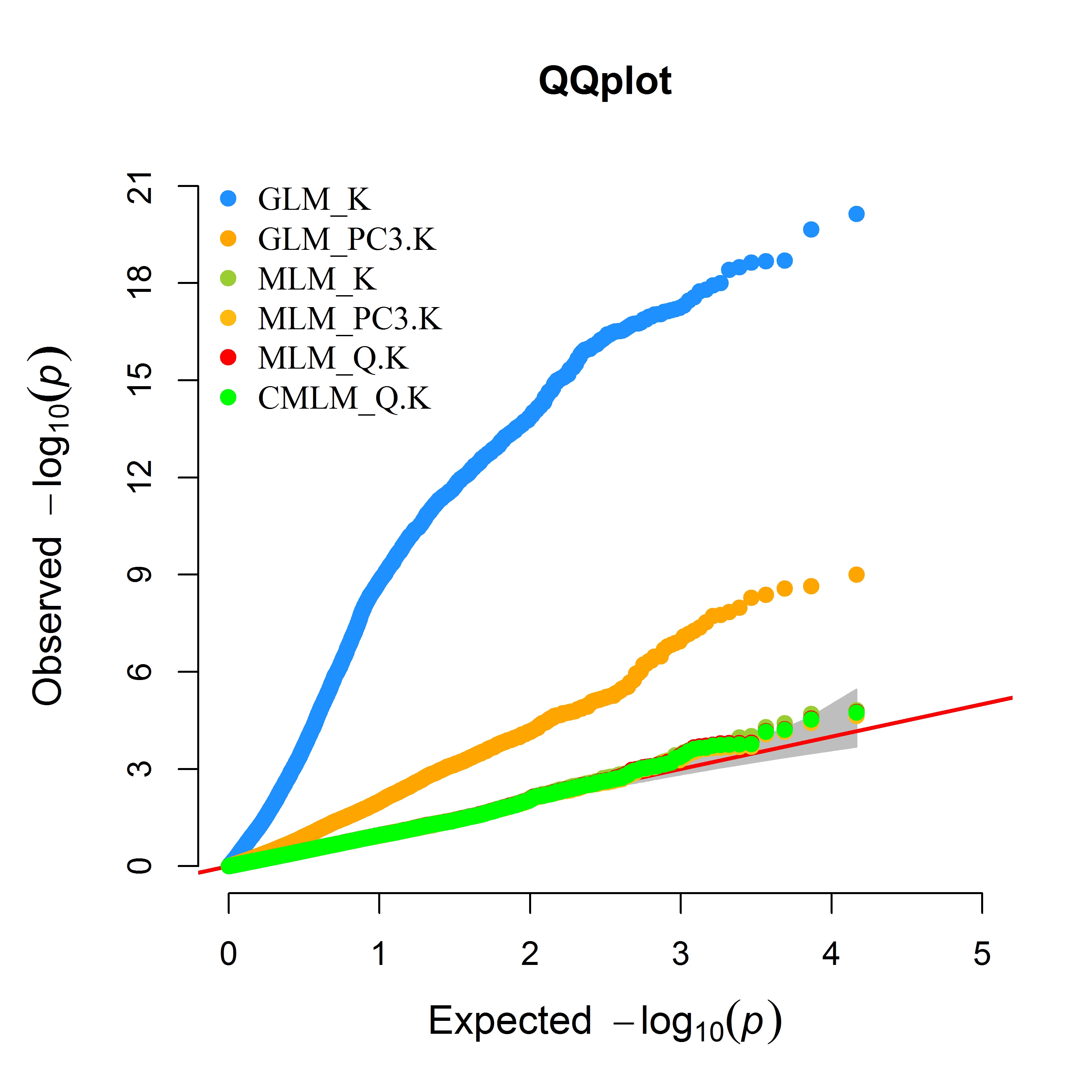

Supplement: FILE S1 — Comparative Q–Q plots of six association models for multiple rust pathotypes and four environments each for YR, LR, and SR. The CMLM was observed as the best fit model. [file Data_Sheet_1.ZIP › Q_Q Plots-Model Comparison/Adult plant stage/LR_E4.jpg]

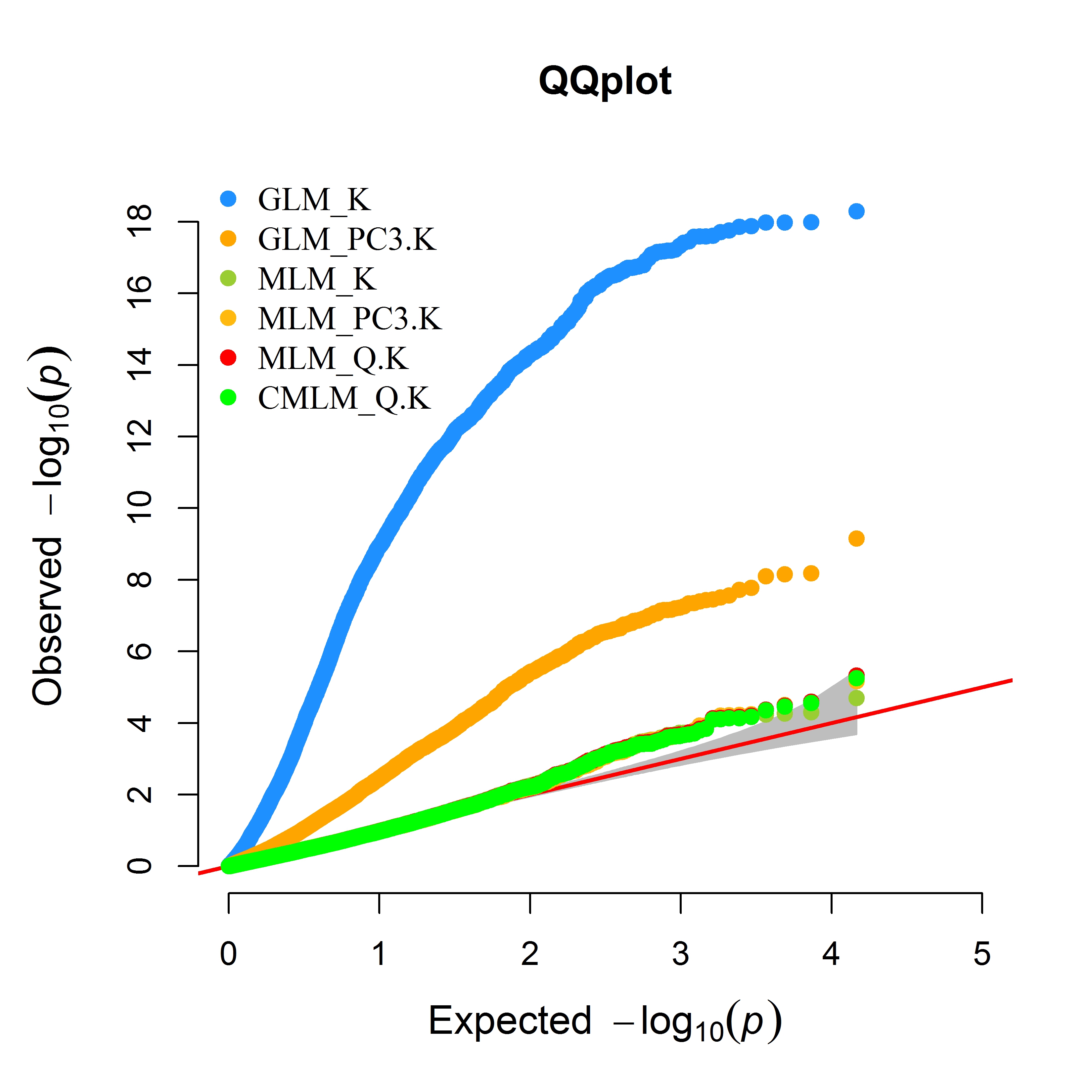

Supplement: FILE S1 — Comparative Q–Q plots of six association models for multiple rust pathotypes and four environments each for YR, LR, and SR. The CMLM was observed as the best fit model. [file Data_Sheet_1.ZIP › Q_Q Plots-Model Comparison/Adult plant stage/SR_E1.jpg]

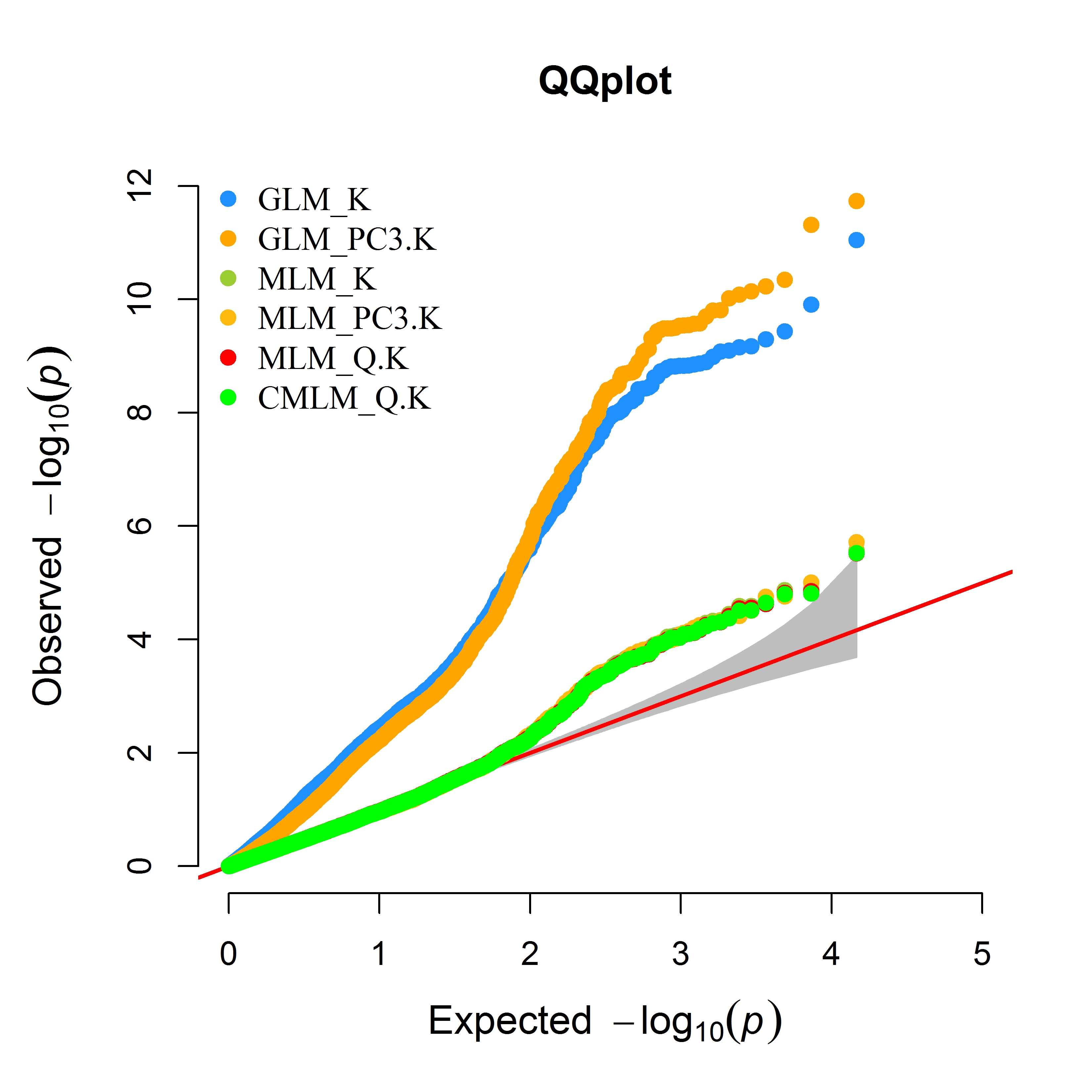

Supplement: FILE S1 — Comparative Q–Q plots of six association models for multiple rust pathotypes and four environments each for YR, LR, and SR. The CMLM was observed as the best fit model. [file Data_Sheet_1.ZIP › Q_Q Plots-Model Comparison/Adult plant stage/SR_E2.jpg]

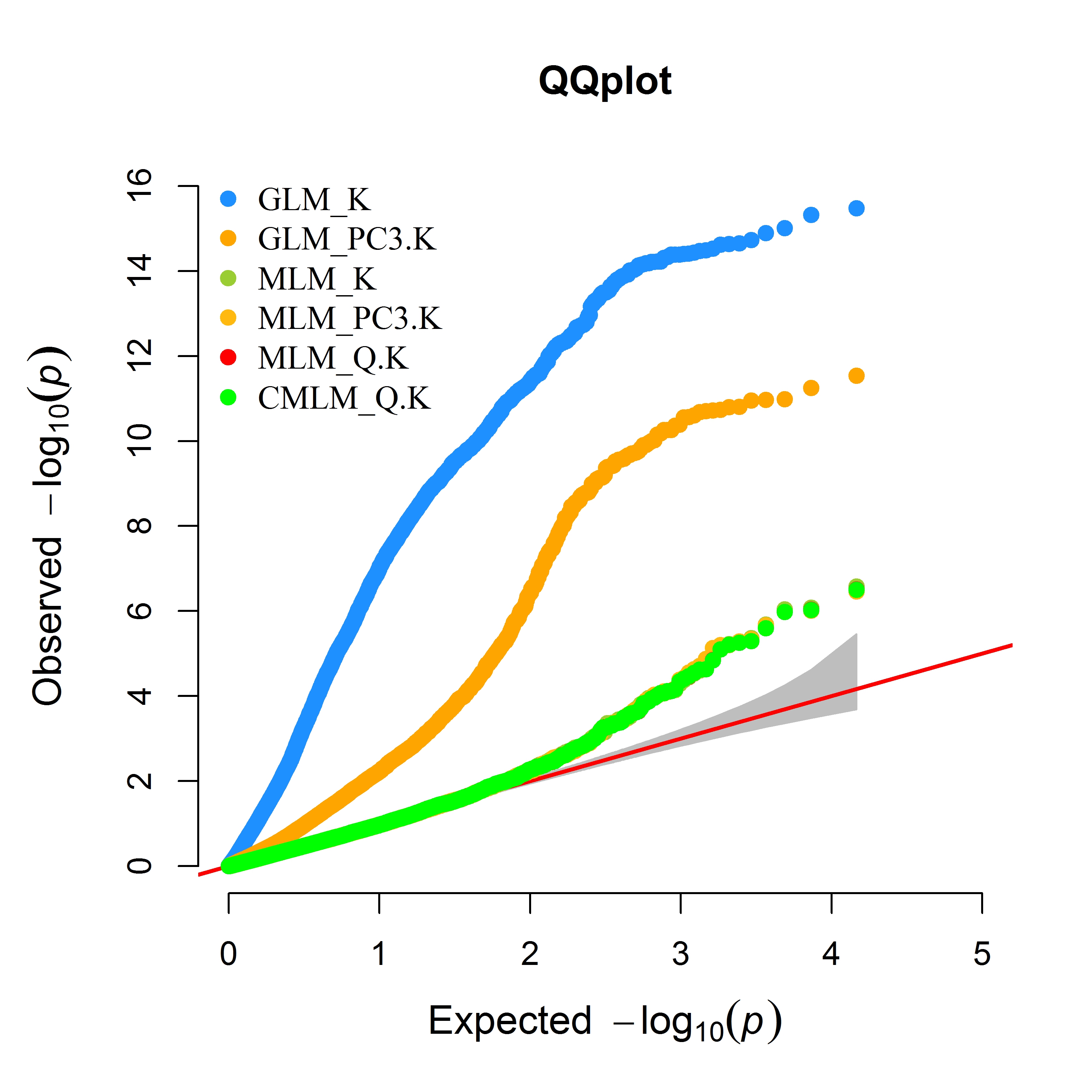

Supplement: FILE S1 — Comparative Q–Q plots of six association models for multiple rust pathotypes and four environments each for YR, LR, and SR. The CMLM was observed as the best fit model. [file Data_Sheet_1.ZIP › Q_Q Plots-Model Comparison/Adult plant stage/SR_E3.jpg]

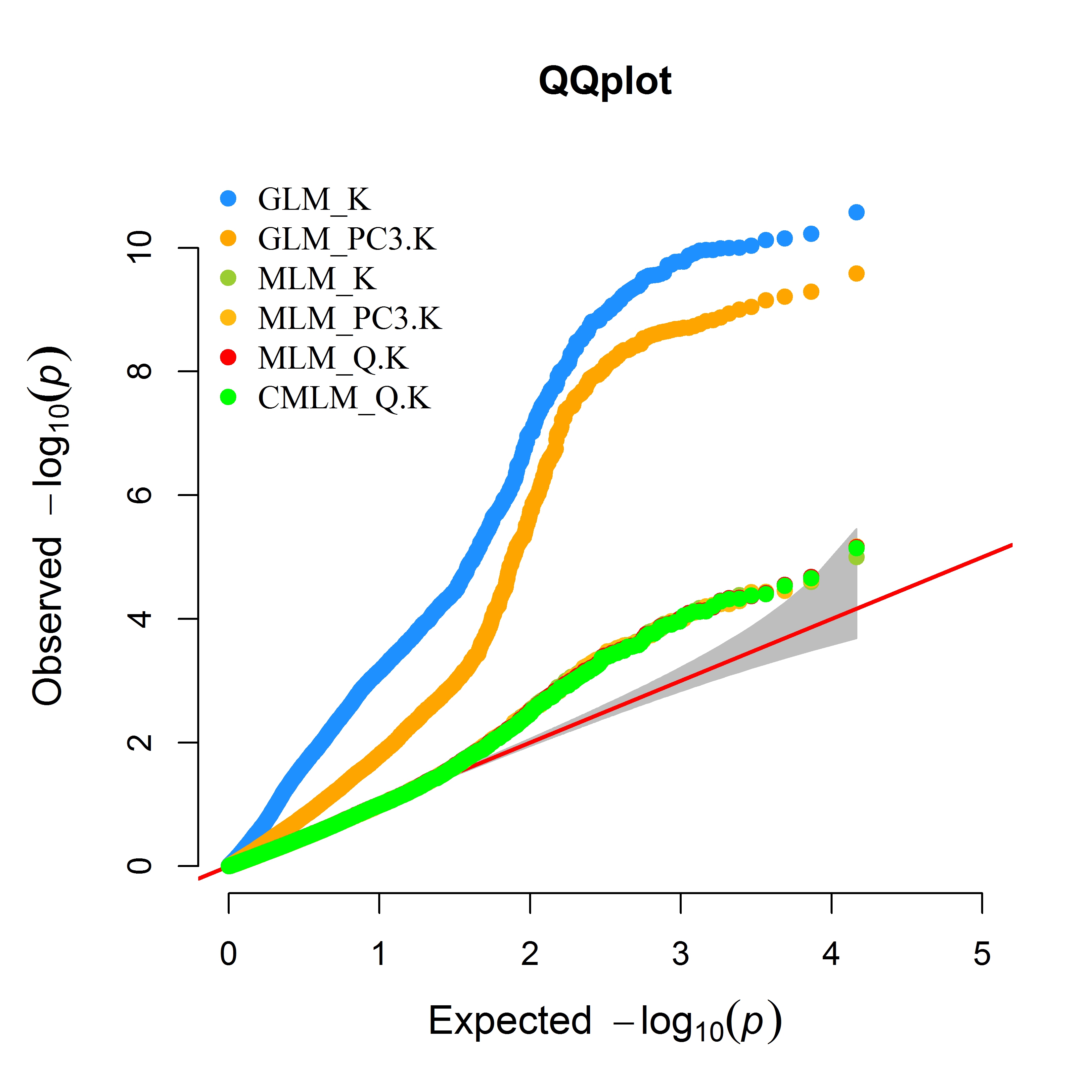

Supplement: FILE S1 — Comparative Q–Q plots of six association models for multiple rust pathotypes and four environments each for YR, LR, and SR. The CMLM was observed as the best fit model. [file Data_Sheet_1.ZIP › Q_Q Plots-Model Comparison/Adult plant stage/SR_E4.jpg]

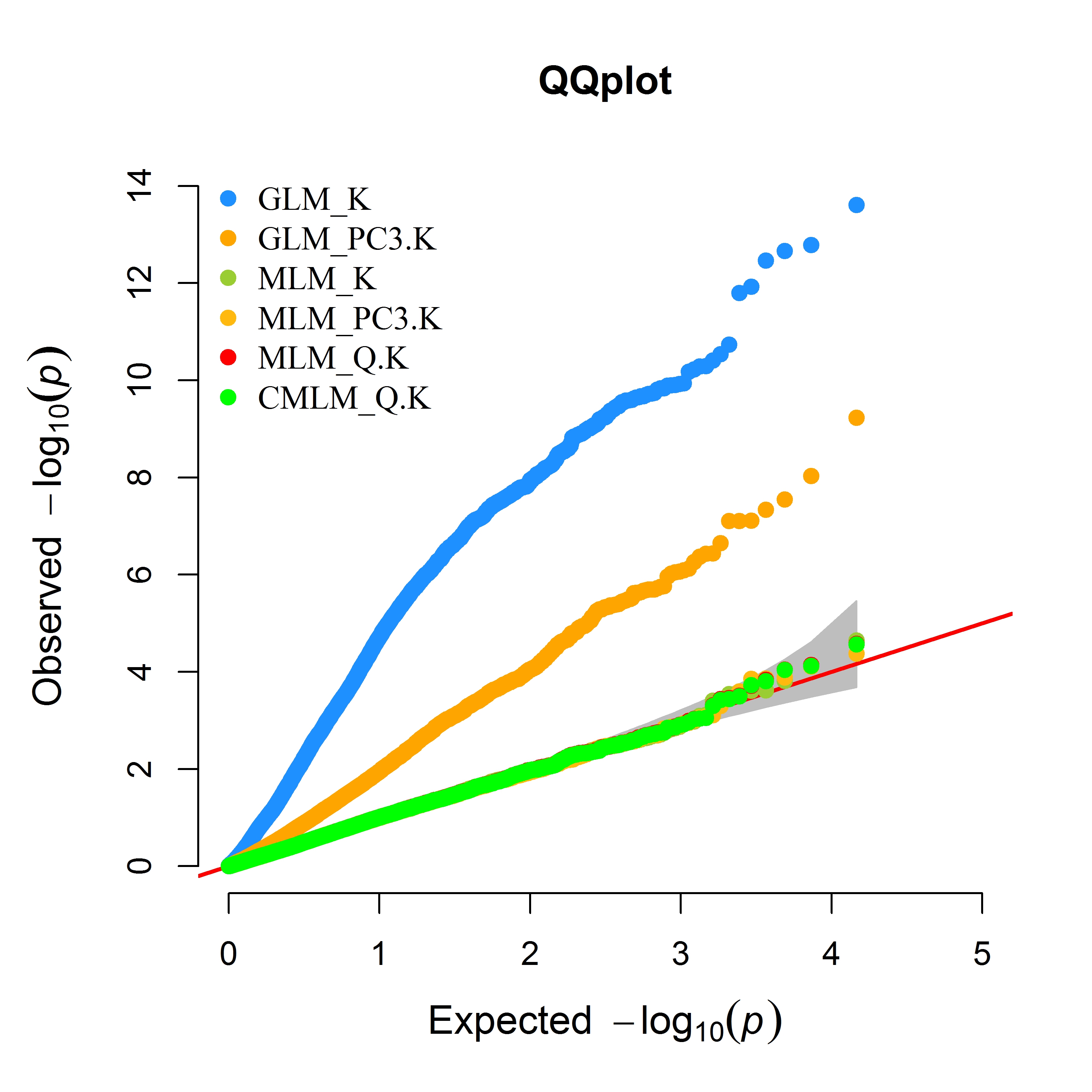

Supplement: FILE S1 — Comparative Q–Q plots of six association models for multiple rust pathotypes and four environments each for YR, LR, and SR. The CMLM was observed as the best fit model. [file Data_Sheet_1.ZIP › Q_Q Plots-Model Comparison/Adult plant stage/YR_E1.jpg]

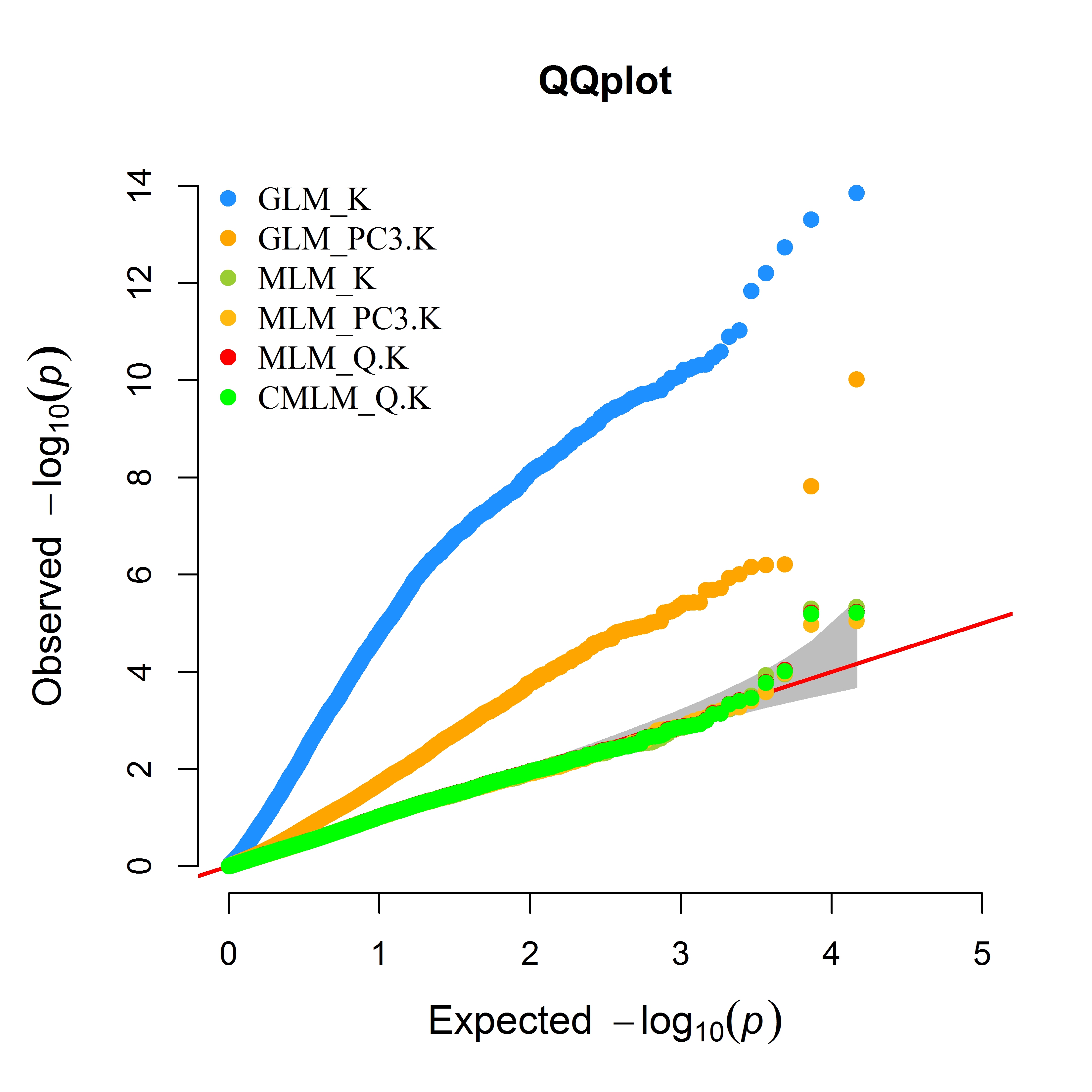

Supplement: FILE S1 — Comparative Q–Q plots of six association models for multiple rust pathotypes and four environments each for YR, LR, and SR. The CMLM was observed as the best fit model. [file Data_Sheet_1.ZIP › Q_Q Plots-Model Comparison/Adult plant stage/YR_E2.jpg]

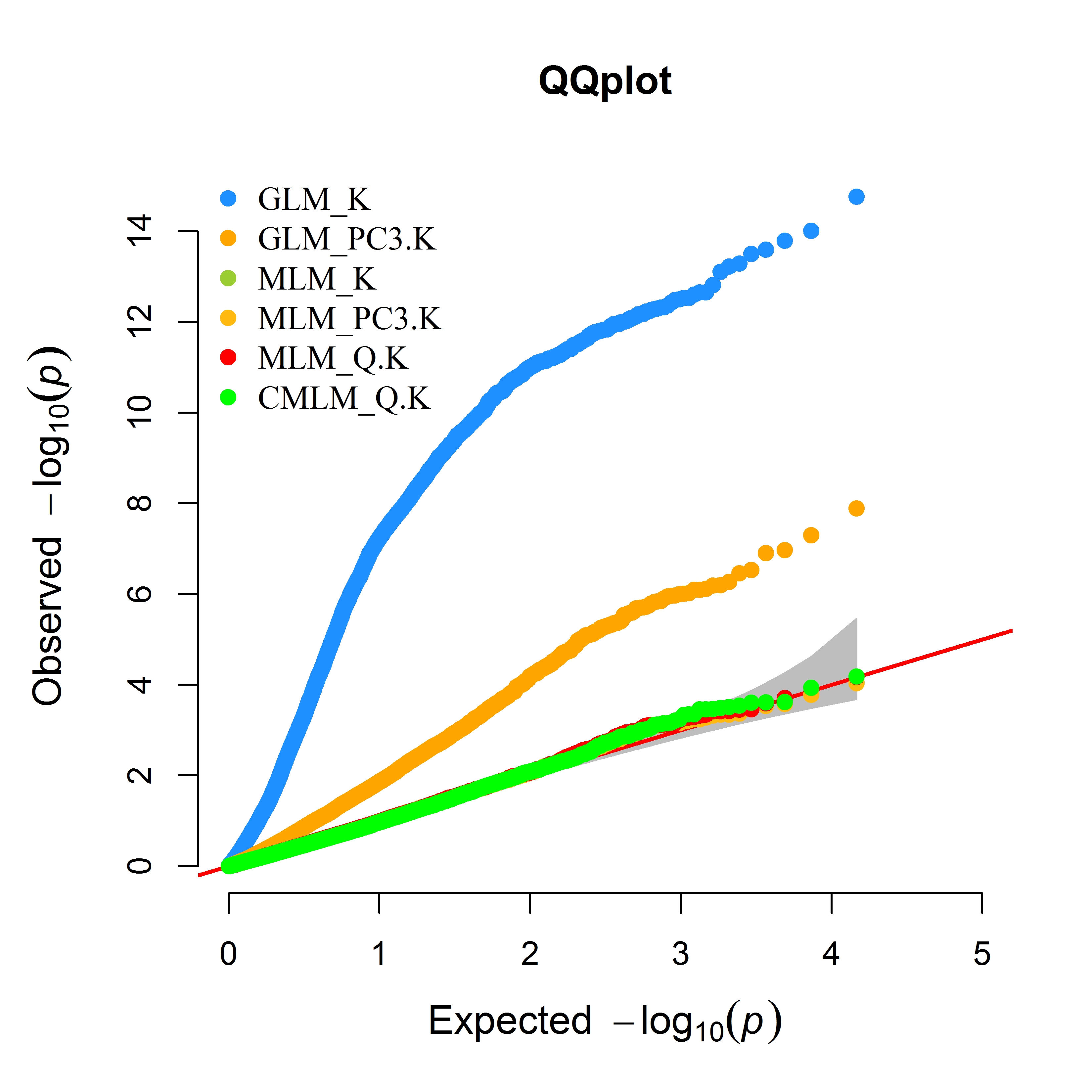

Supplement: FILE S1 — Comparative Q–Q plots of six association models for multiple rust pathotypes and four environments each for YR, LR, and SR. The CMLM was observed as the best fit model. [file Data_Sheet_1.ZIP › Q_Q Plots-Model Comparison/Adult plant stage/YR_E3.jpg]

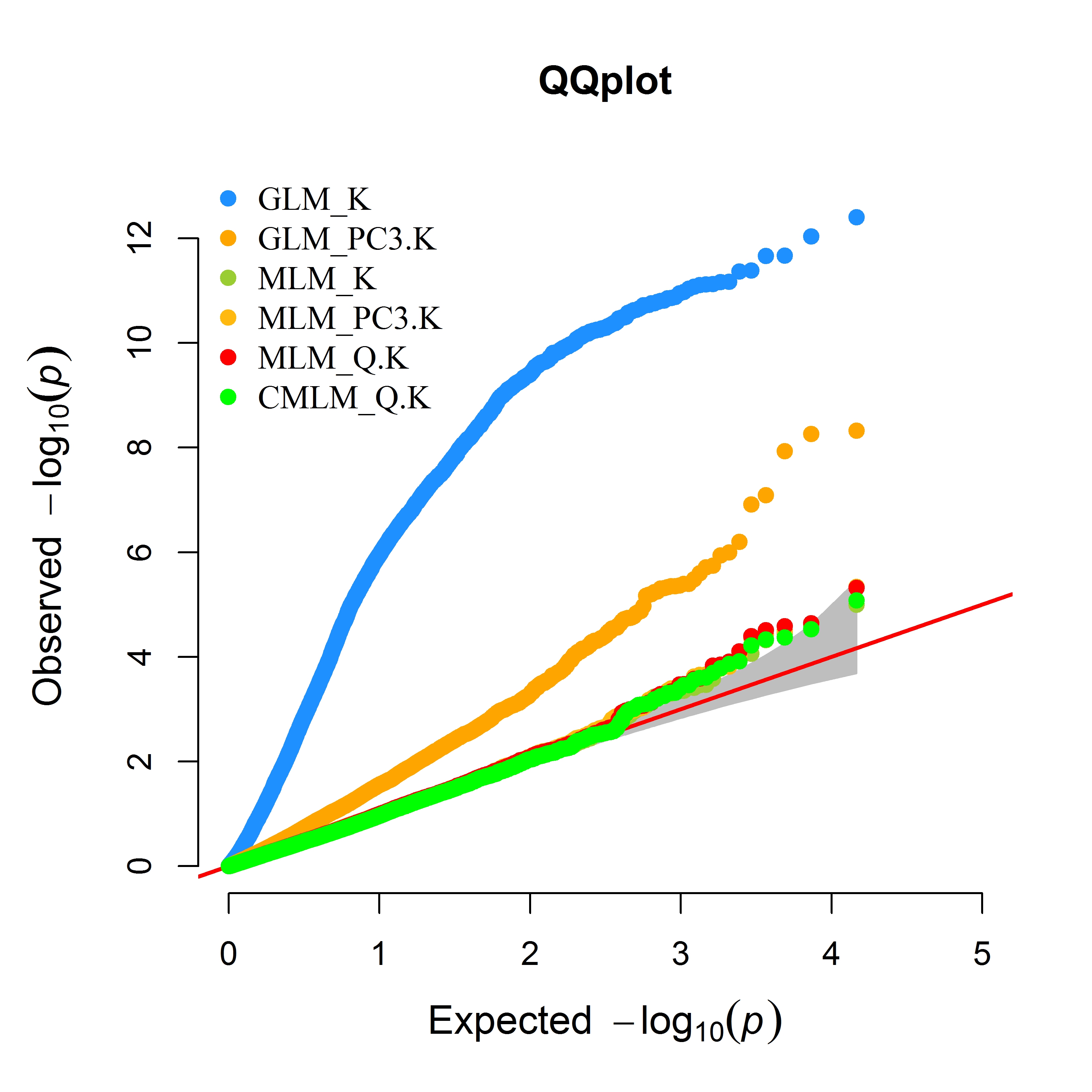

Supplement: FILE S1 — Comparative Q–Q plots of six association models for multiple rust pathotypes and four environments each for YR, LR, and SR. The CMLM was observed as the best fit model. [file Data_Sheet_1.ZIP › Q_Q Plots-Model Comparison/Adult plant stage/YR_E4.jpg]

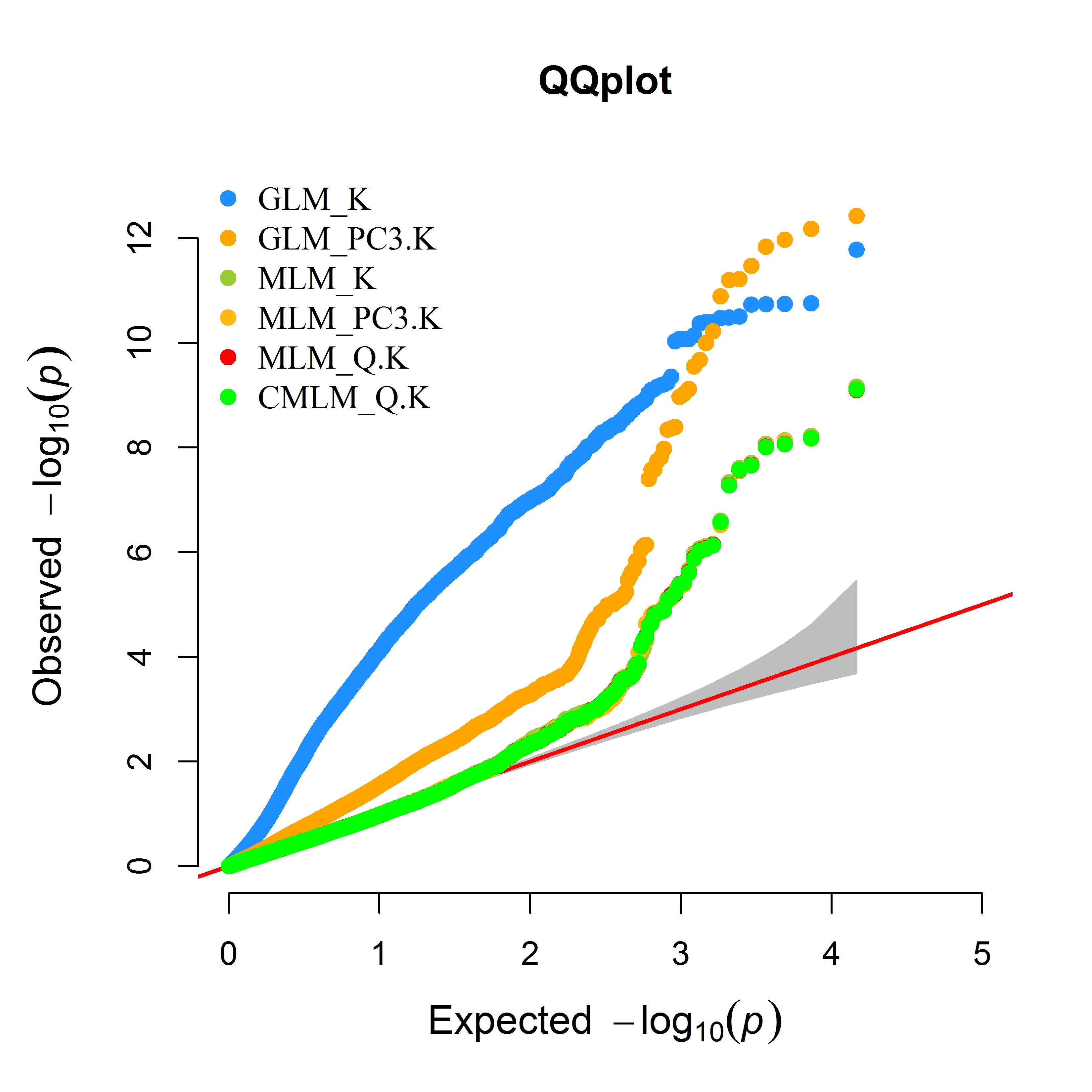

Supplement: FILE S1 — Comparative Q–Q plots of six association models for multiple rust pathotypes and four environments each for YR, LR, and SR. The CMLM was observed as the best fit model. [file Data_Sheet_1.ZIP › Q_Q Plots-Model Comparison/Seedling stage/LR_104-2.jpg]

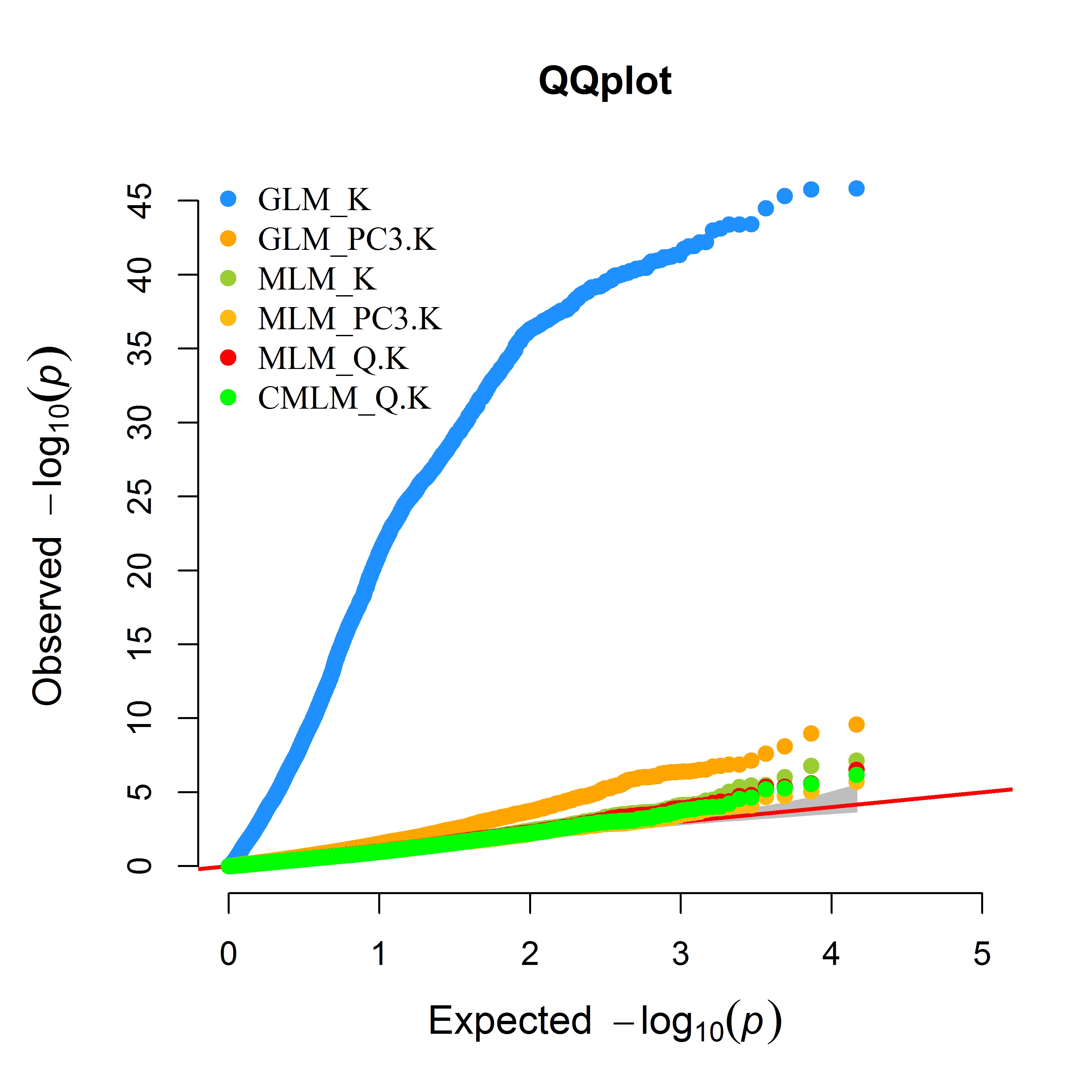

Supplement: FILE S1 — Comparative Q–Q plots of six association models for multiple rust pathotypes and four environments each for YR, LR, and SR. The CMLM was observed as the best fit model. [file Data_Sheet_1.ZIP › Q_Q Plots-Model Comparison/Seedling stage/LR_106.jpg]

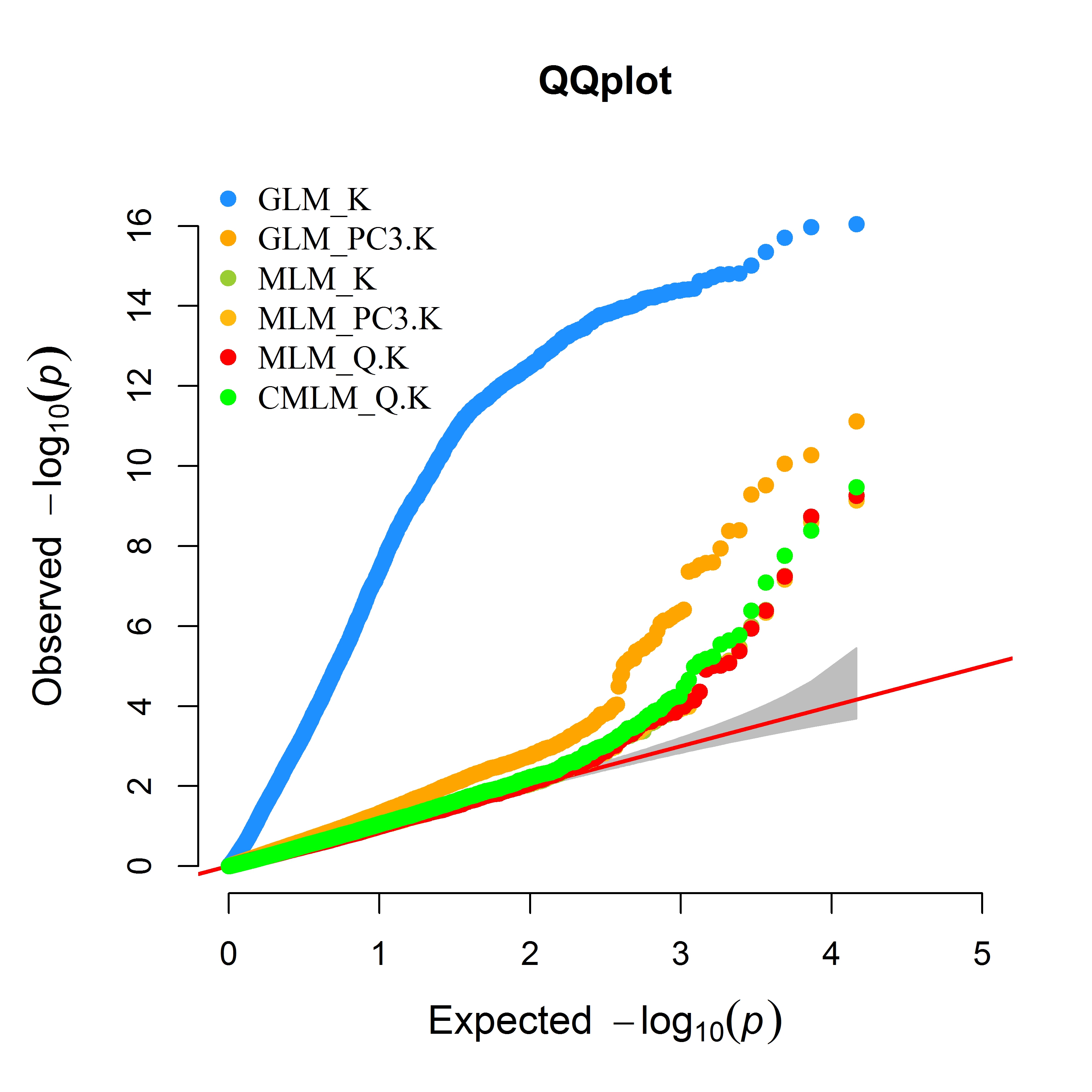

Supplement: FILE S1 — Comparative Q–Q plots of six association models for multiple rust pathotypes and four environments each for YR, LR, and SR. The CMLM was observed as the best fit model. [file Data_Sheet_1.ZIP › Q_Q Plots-Model Comparison/Seedling stage/LR_12-5.jpg]

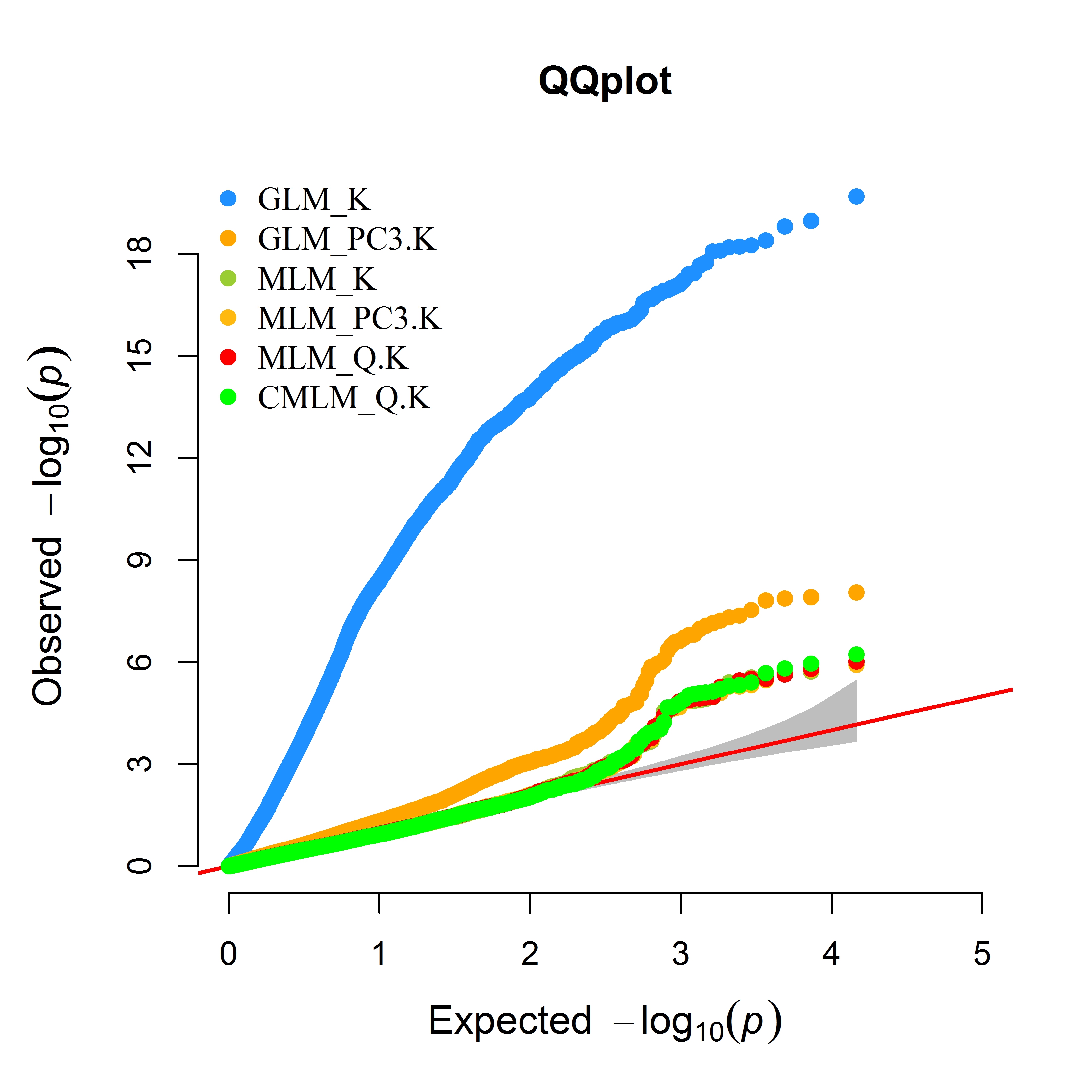

Supplement: FILE S1 — Comparative Q–Q plots of six association models for multiple rust pathotypes and four environments each for YR, LR, and SR. The CMLM was observed as the best fit model. [file Data_Sheet_1.ZIP › Q_Q Plots-Model Comparison/Seedling stage/LR_77-1.jpg]

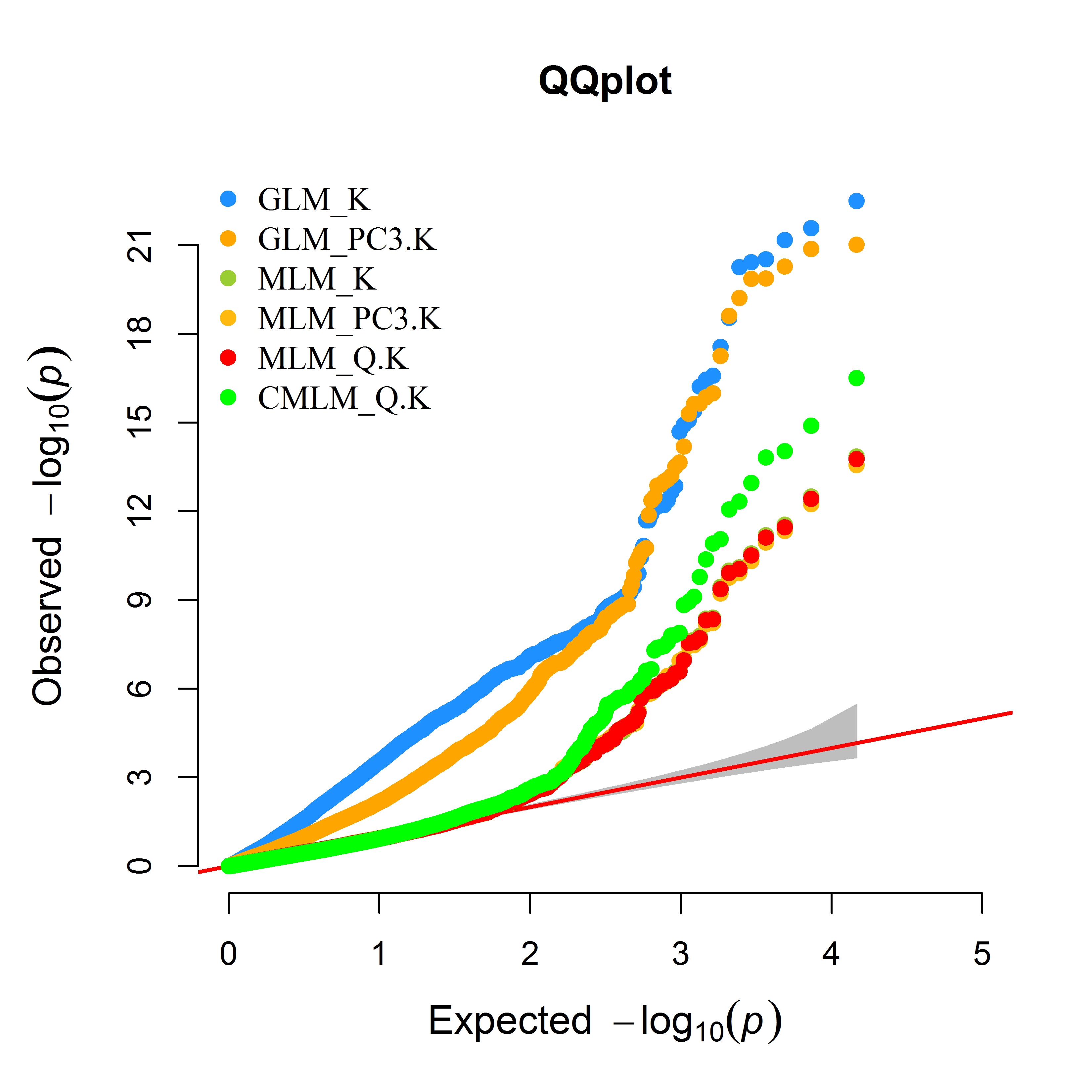

Supplement: FILE S1 — Comparative Q–Q plots of six association models for multiple rust pathotypes and four environments each for YR, LR, and SR. The CMLM was observed as the best fit model. [file Data_Sheet_1.ZIP › Q_Q Plots-Model Comparison/Seedling stage/LR_77-5.jpg]

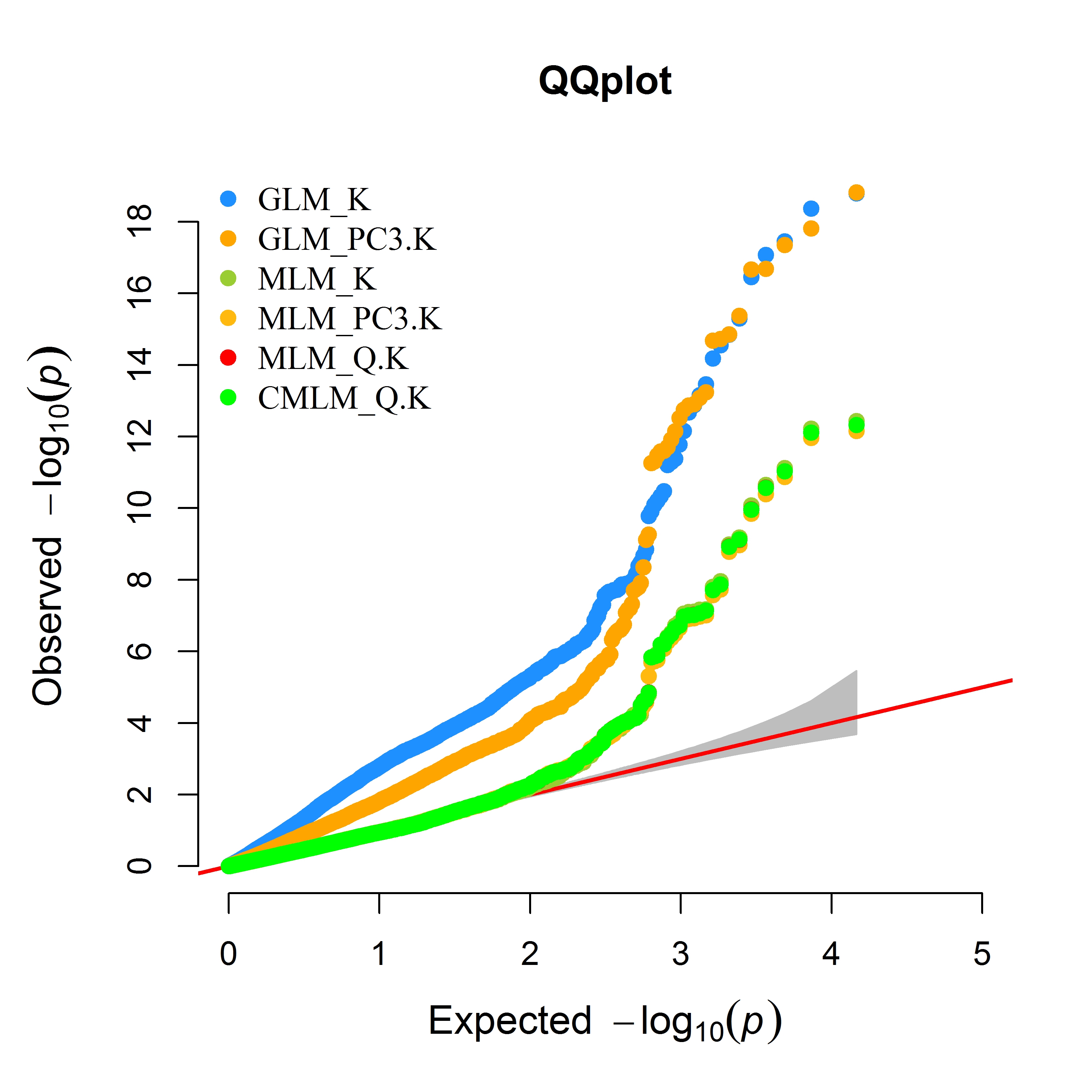

Supplement: FILE S1 — Comparative Q–Q plots of six association models for multiple rust pathotypes and four environments each for YR, LR, and SR. The CMLM was observed as the best fit model. [file Data_Sheet_1.ZIP › Q_Q Plots-Model Comparison/Seedling stage/LR_77-9.jpg]

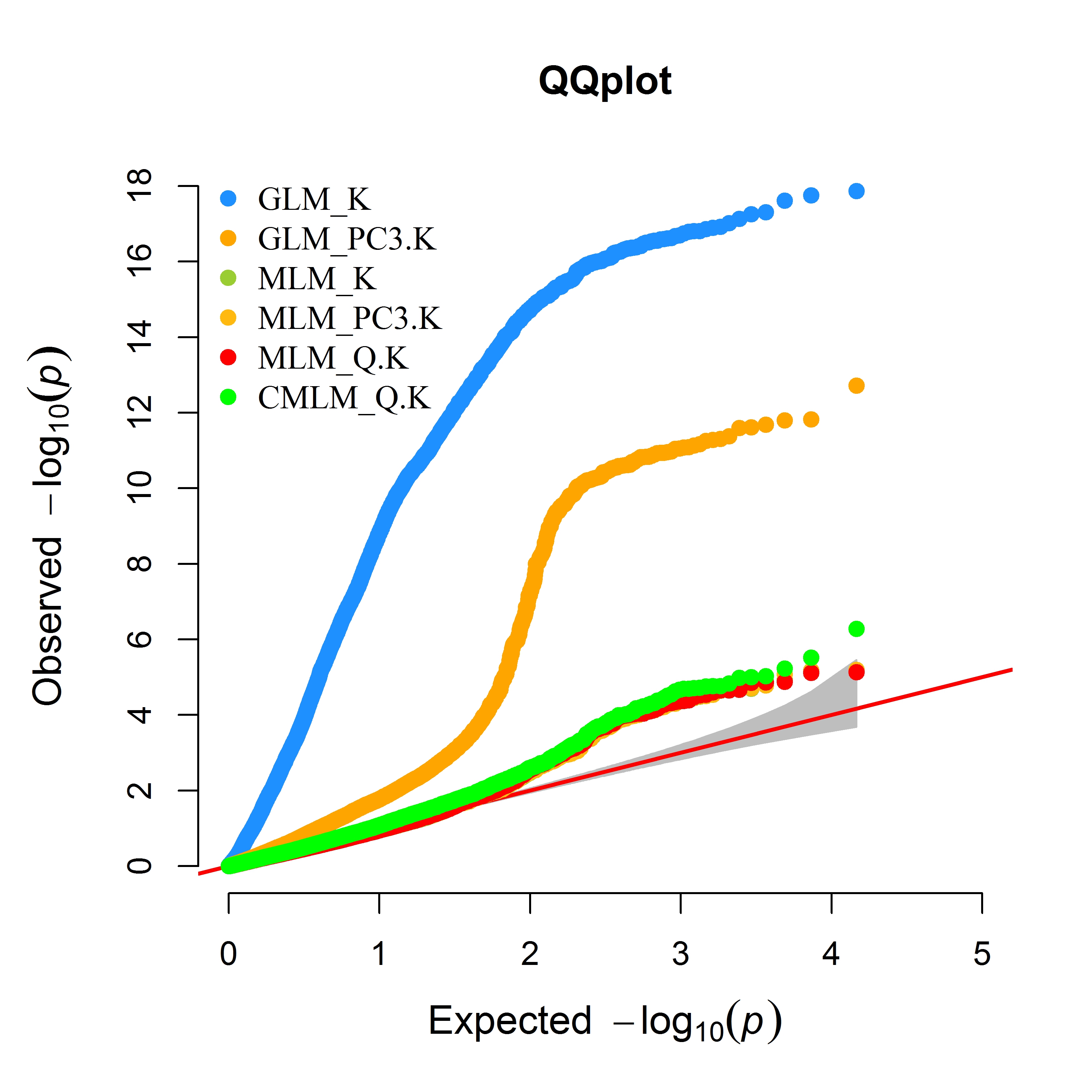

Supplement: FILE S1 — Comparative Q–Q plots of six association models for multiple rust pathotypes and four environments each for YR, LR, and SR. The CMLM was observed as the best fit model. [file Data_Sheet_1.ZIP › Q_Q Plots-Model Comparison/Seedling stage/SR_11.jpg]

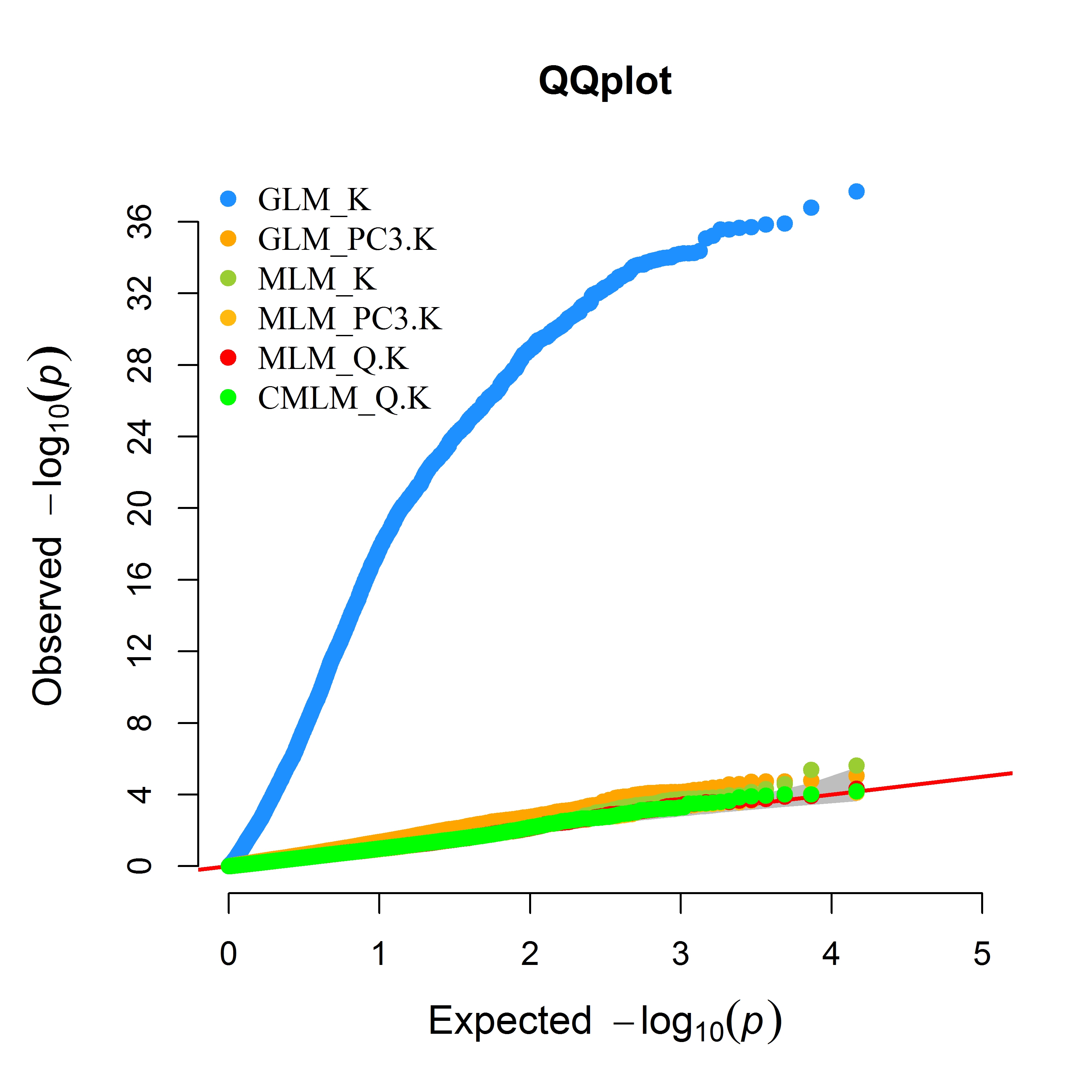

Supplement: FILE S1 — Comparative Q–Q plots of six association models for multiple rust pathotypes and four environments each for YR, LR, and SR. The CMLM was observed as the best fit model. [file Data_Sheet_1.ZIP › Q_Q Plots-Model Comparison/Seedling stage/SR_117-6.jpg]

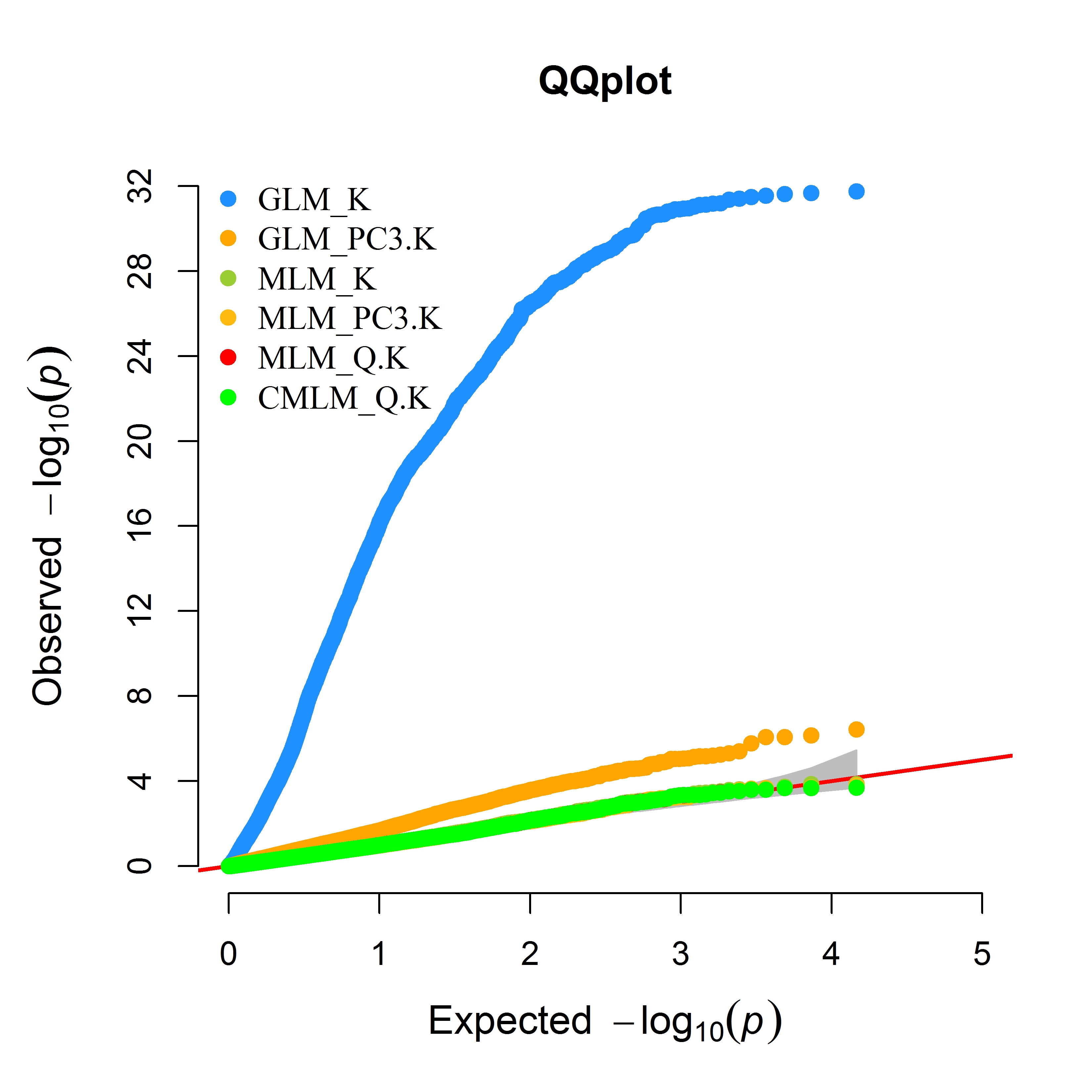

Supplement: FILE S1 — Comparative Q–Q plots of six association models for multiple rust pathotypes and four environments each for YR, LR, and SR. The CMLM was observed as the best fit model. [file Data_Sheet_1.ZIP › Q_Q Plots-Model Comparison/Seedling stage/SR_122.jpg]

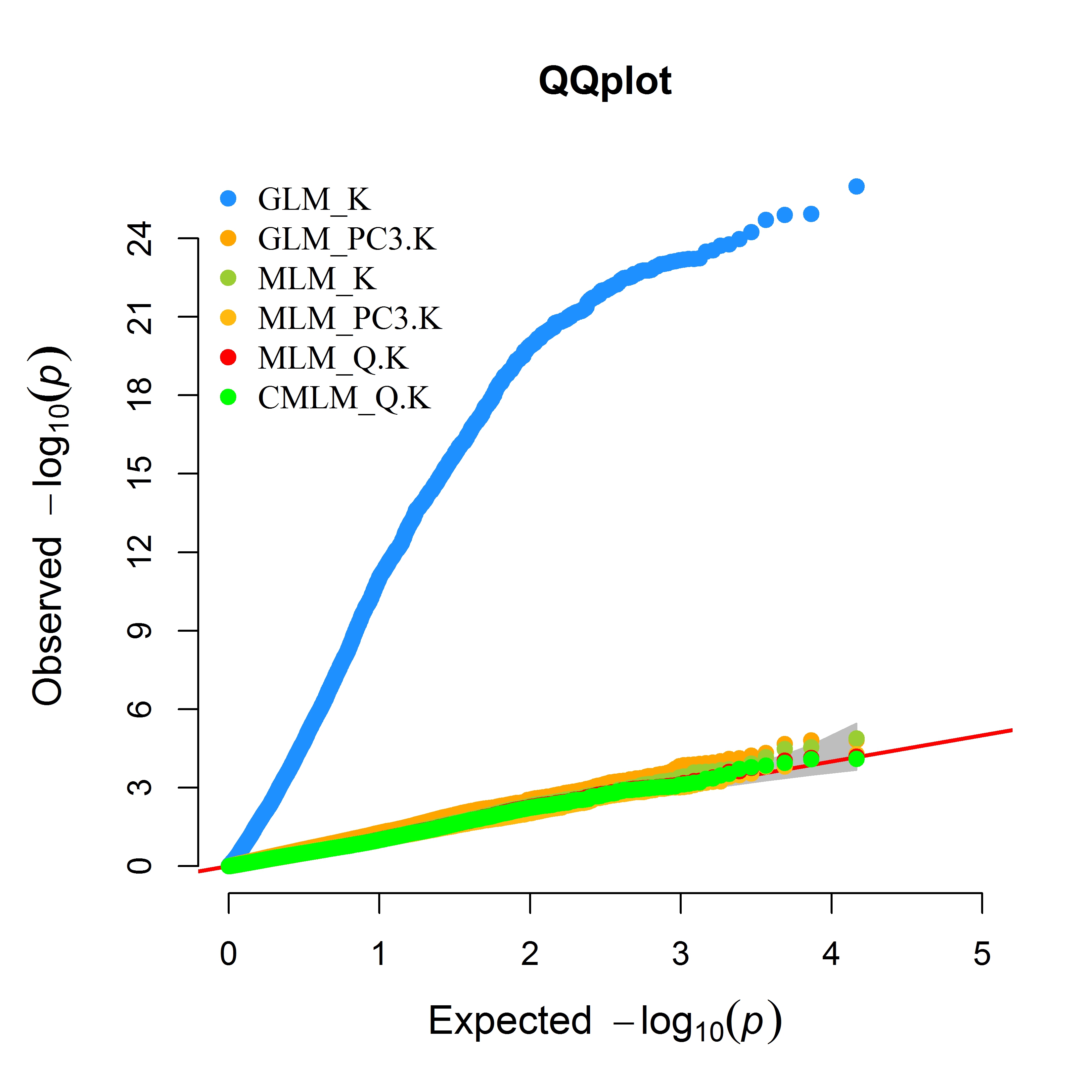

Supplement: FILE S1 — Comparative Q–Q plots of six association models for multiple rust pathotypes and four environments each for YR, LR, and SR. The CMLM was observed as the best fit model. [file Data_Sheet_1.ZIP › Q_Q Plots-Model Comparison/Seedling stage/SR_21A2.jpg]

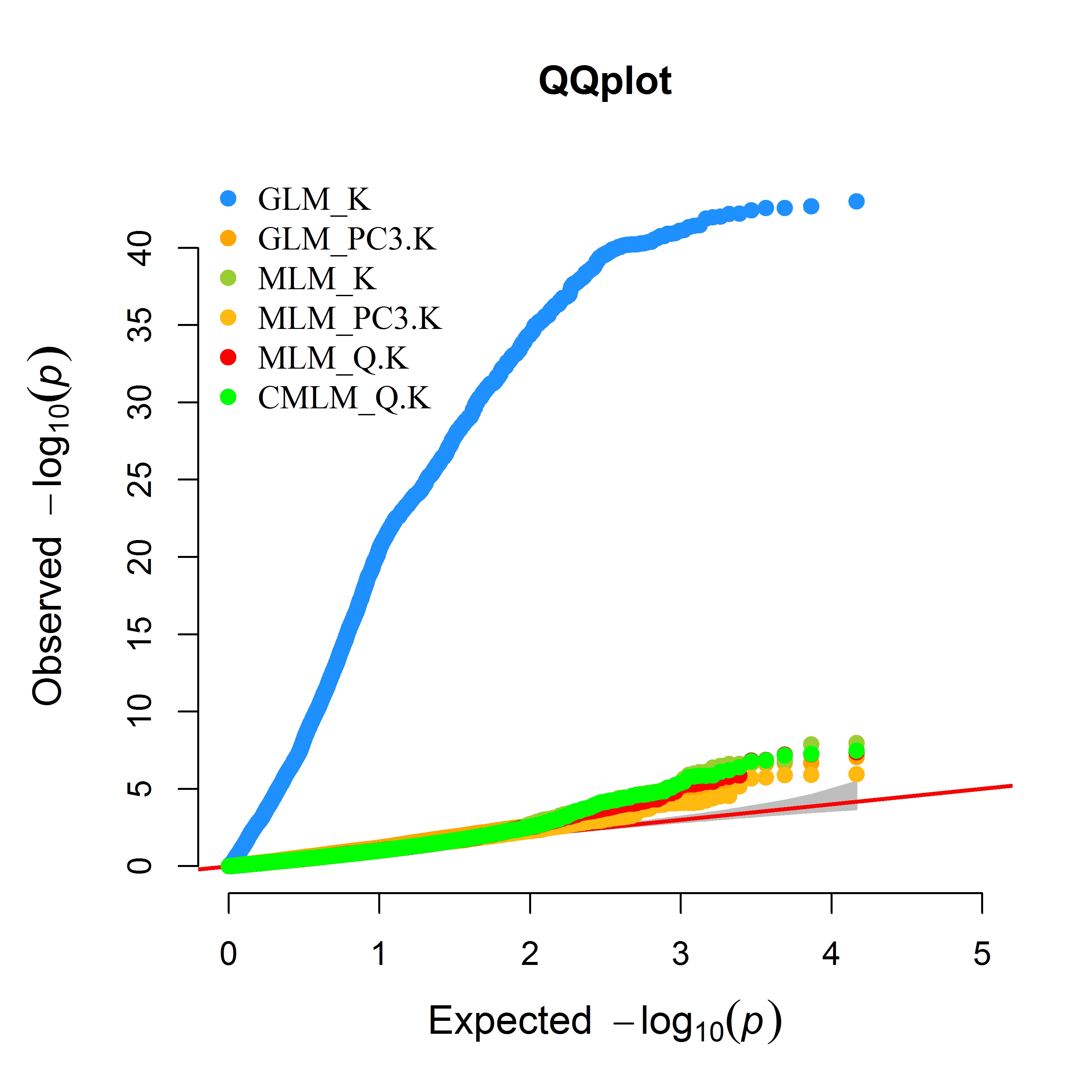

Supplement: FILE S1 — Comparative Q–Q plots of six association models for multiple rust pathotypes and four environments each for YR, LR, and SR. The CMLM was observed as the best fit model. [file Data_Sheet_1.ZIP › Q_Q Plots-Model Comparison/Seedling stage/SR_34-1.jpg]

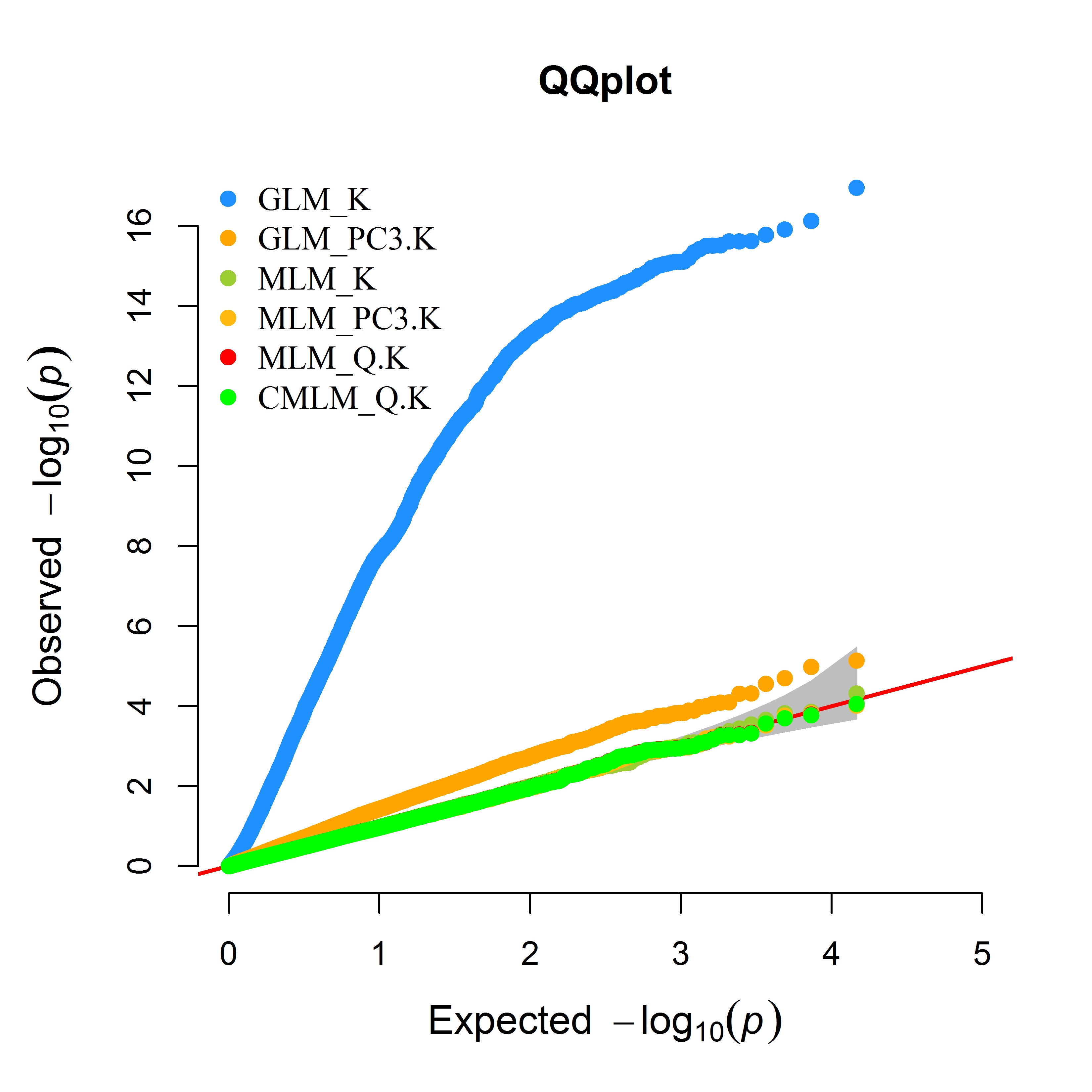

Supplement: FILE S1 — Comparative Q–Q plots of six association models for multiple rust pathotypes and four environments each for YR, LR, and SR. The CMLM was observed as the best fit model. [file Data_Sheet_1.ZIP › Q_Q Plots-Model Comparison/Seedling stage/SR_40-3.jpg]

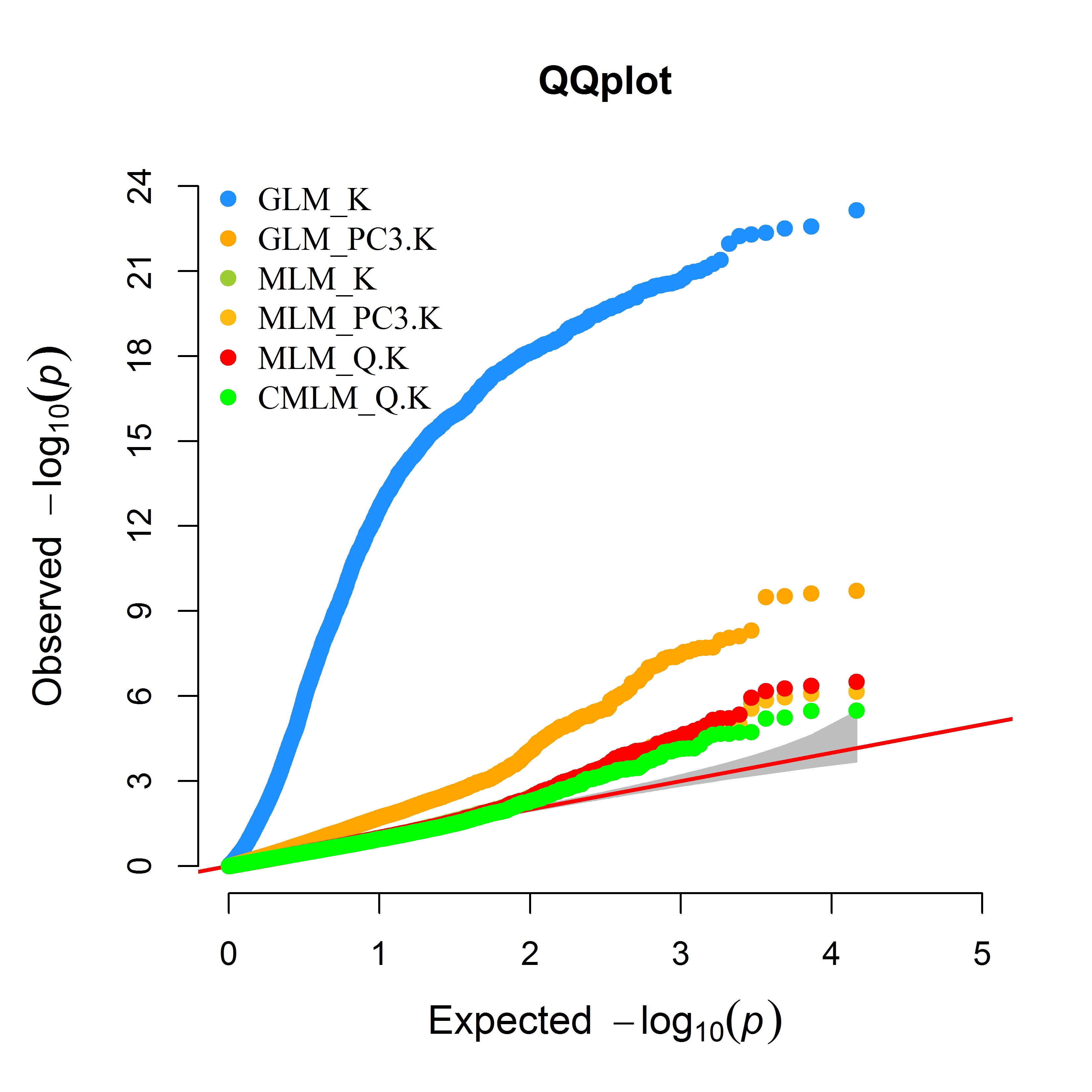

Supplement: FILE S1 — Comparative Q–Q plots of six association models for multiple rust pathotypes and four environments each for YR, LR, and SR. The CMLM was observed as the best fit model. [file Data_Sheet_1.ZIP › Q_Q Plots-Model Comparison/Seedling stage/SR_40A.jpg]

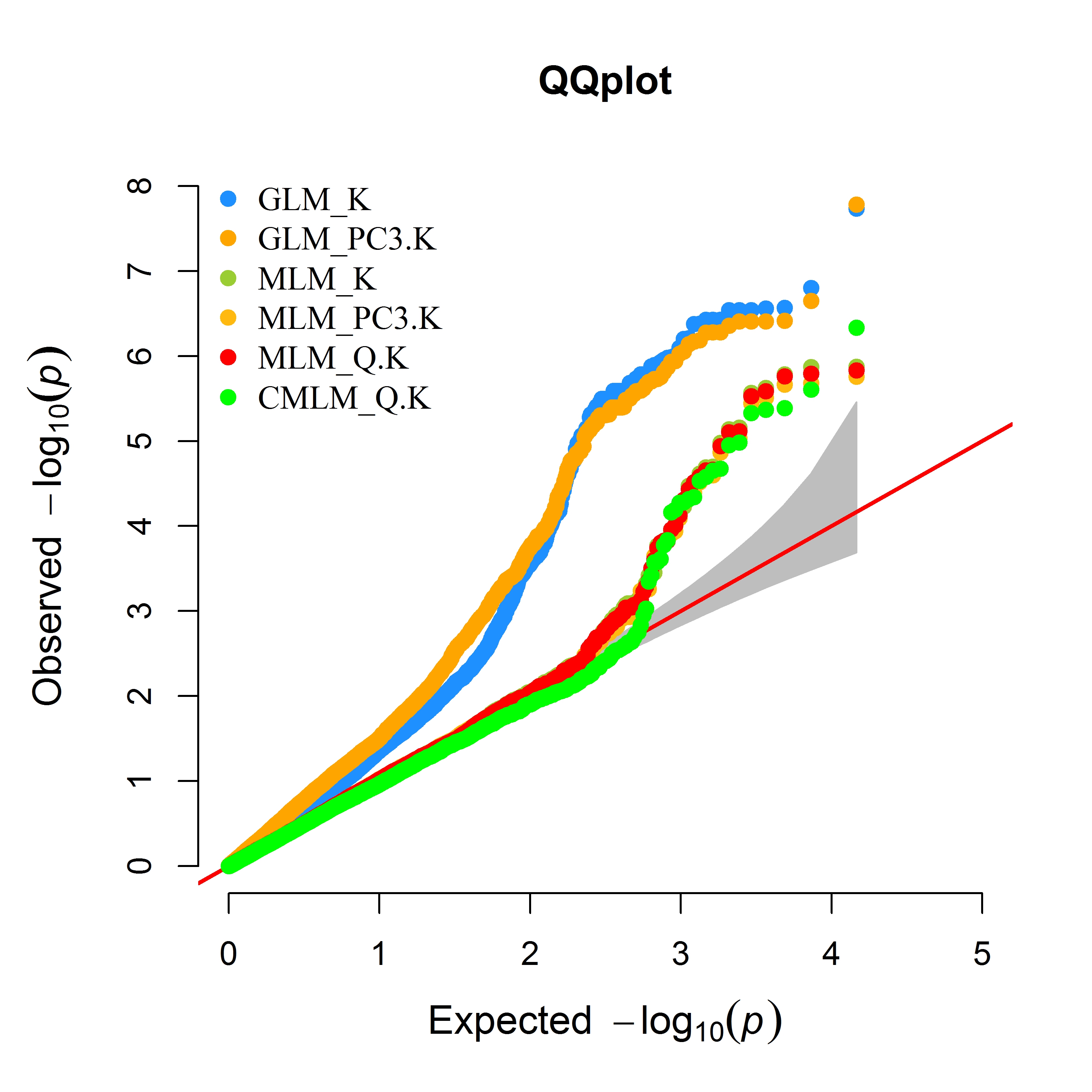

Supplement: FILE S1 — Comparative Q–Q plots of six association models for multiple rust pathotypes and four environments each for YR, LR, and SR. The CMLM was observed as the best fit model. [file Data_Sheet_1.ZIP › Q_Q Plots-Model Comparison/Seedling stage/YR_110S119.jpg]

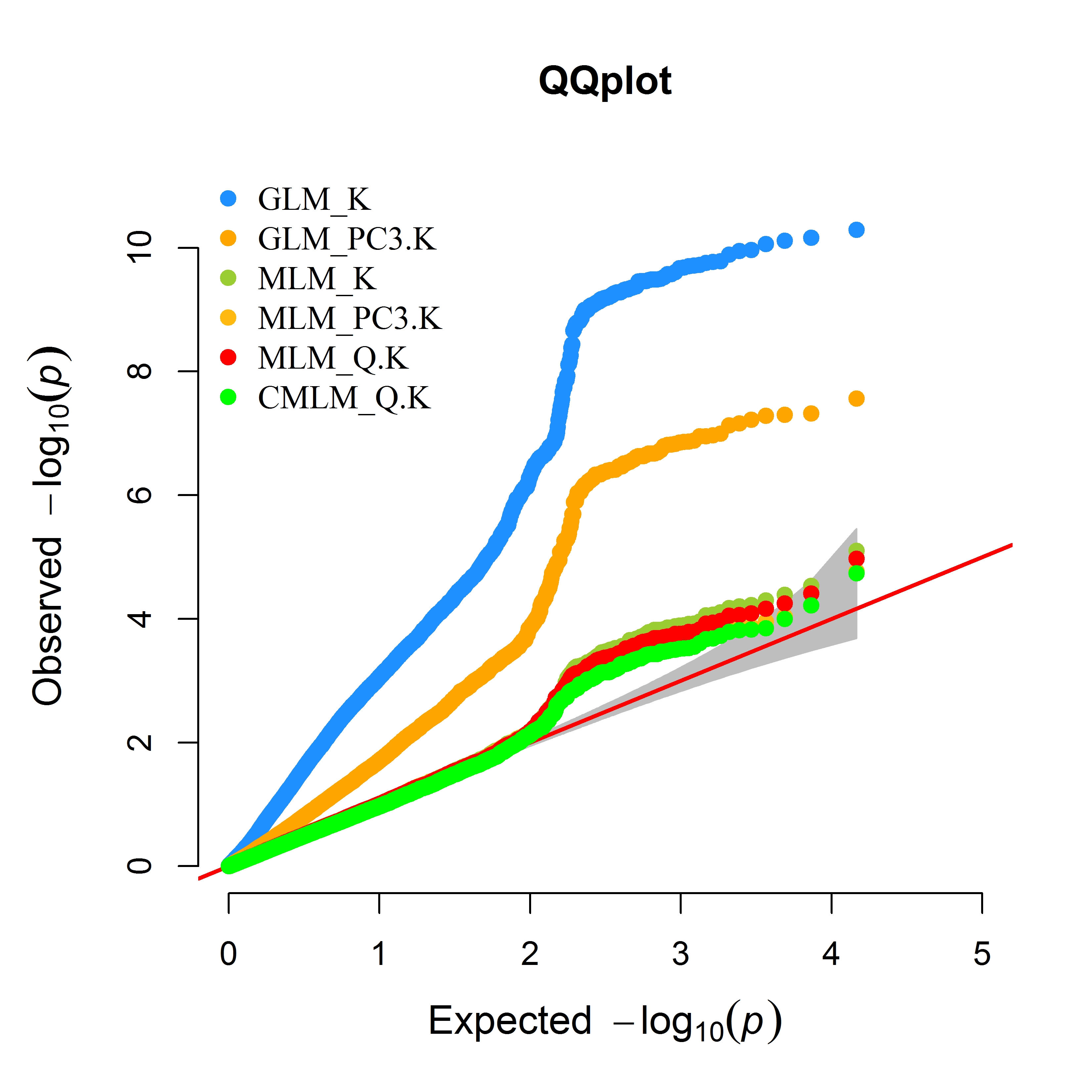

Supplement: FILE S1 — Comparative Q–Q plots of six association models for multiple rust pathotypes and four environments each for YR, LR, and SR. The CMLM was observed as the best fit model. [file Data_Sheet_1.ZIP › Q_Q Plots-Model Comparison/Seedling stage/YR_110S84.jpg]

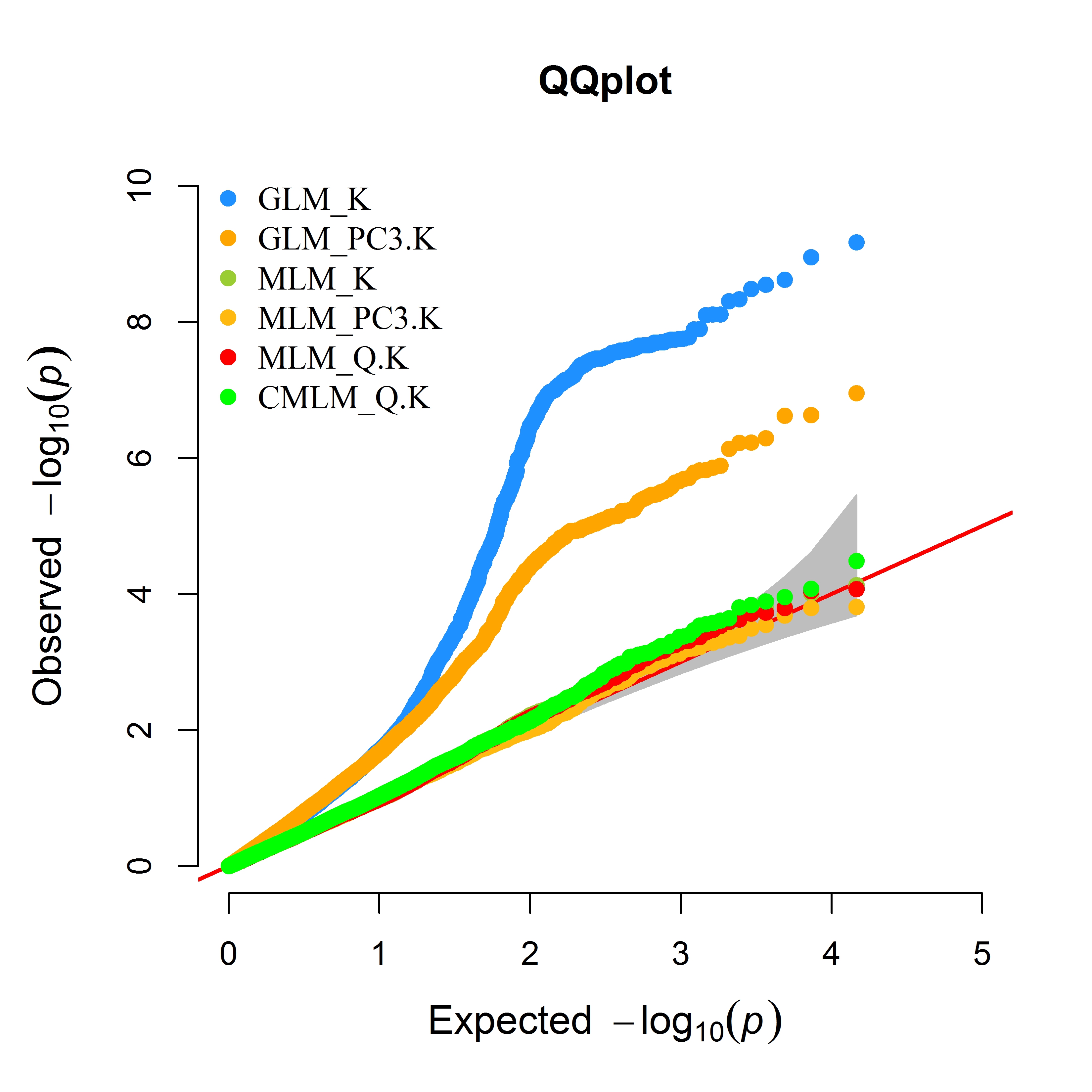

Supplement: FILE S1 — Comparative Q–Q plots of six association models for multiple rust pathotypes and four environments each for YR, LR, and SR. The CMLM was observed as the best fit model. [file Data_Sheet_1.ZIP › Q_Q Plots-Model Comparison/Seedling stage/YR_238S119.jpg]

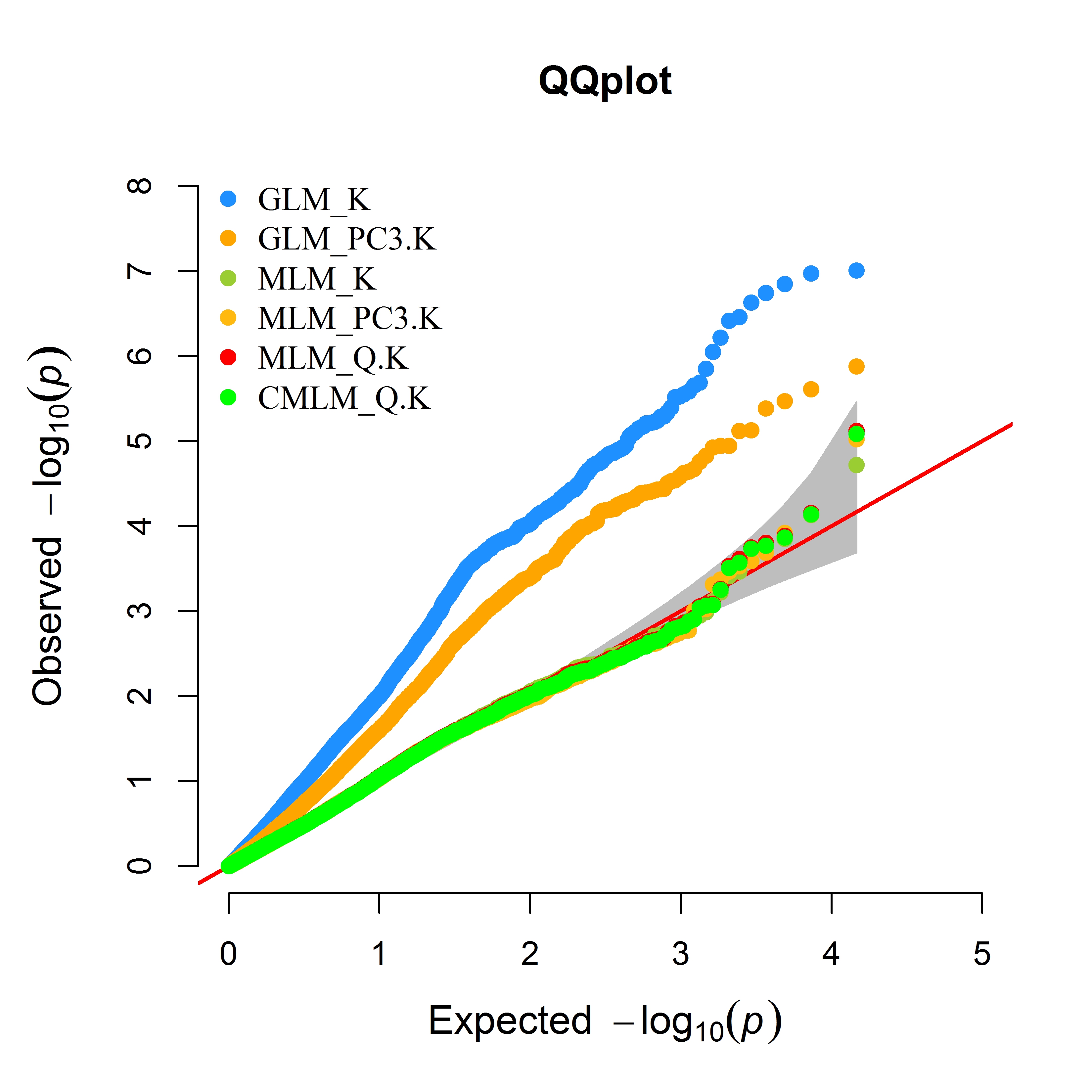

Supplement: FILE S1 — Comparative Q–Q plots of six association models for multiple rust pathotypes and four environments each for YR, LR, and SR. The CMLM was observed as the best fit model. [file Data_Sheet_1.ZIP › Q_Q Plots-Model Comparison/Seedling stage/YR_46S119.jpg]

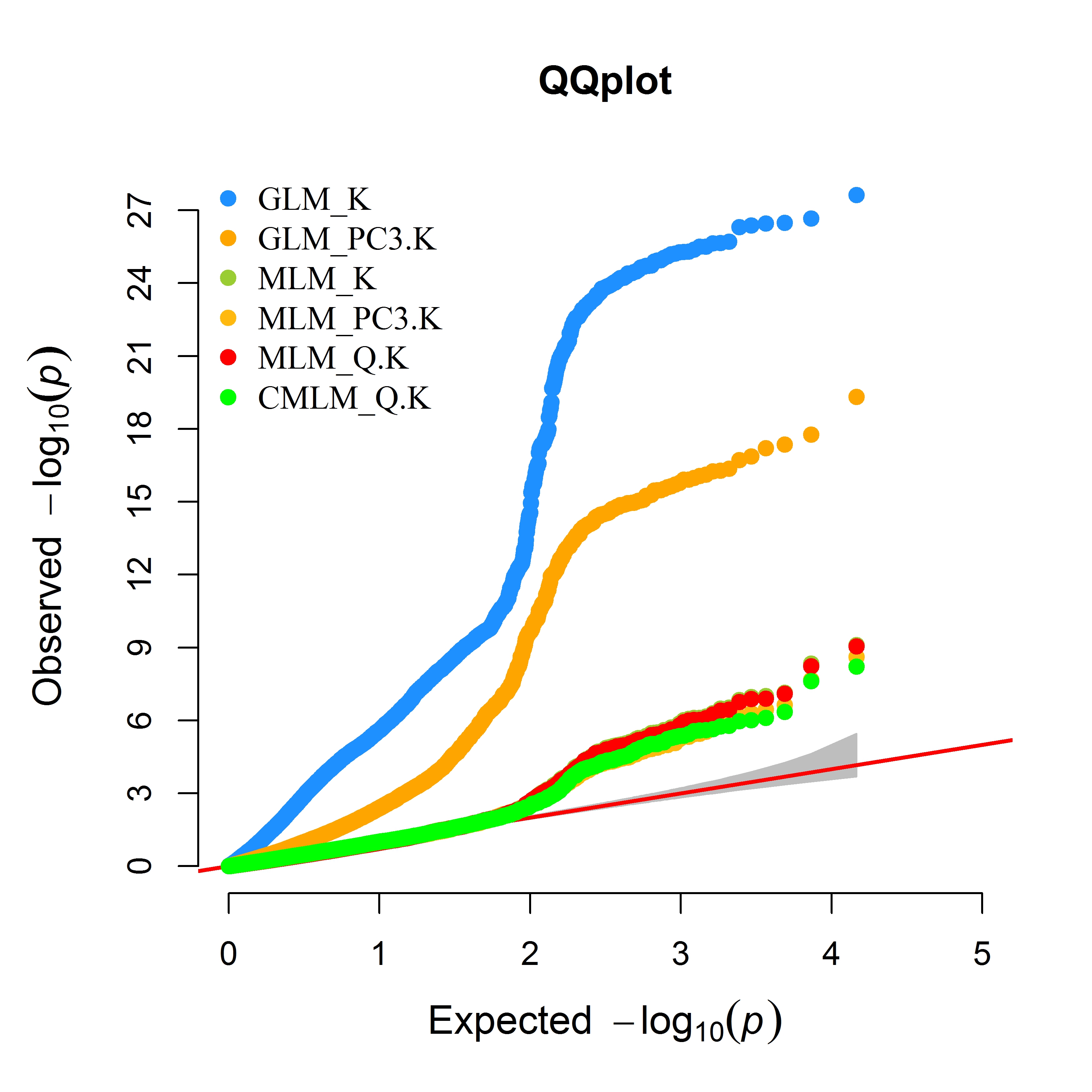

Supplement: FILE S1 — Comparative Q–Q plots of six association models for multiple rust pathotypes and four environments each for YR, LR, and SR. The CMLM was observed as the best fit model. [file Data_Sheet_1.ZIP › Q_Q Plots-Model Comparison/Seedling stage/YR_T.jpg]
