# Supplementary material for: Genome-Wide Association Studies in Diverse Spring Wheat Panel for Stripe, Stem, and Leaf Rust Resistance
Source: Front Plant Sci. 2020 Jun 3;11:748. doi: 10.3389/fpls.2020.00748 (PMC7286347; doi:10.3389/fpls.2020.00748)
Supplement: TABLE S1 — Avirulence/virulence formula of predominant Indian wheat rust pathotypes used in the present study. [file Table_1.DOCX]

Supplementary Table S1: Avirulence/ virulence formula of predominant Indian wheat rust pathotypes used in the present study

| **sl.no.** | **Pathotype** | **Avirulence** | **Virulence** |
| --- | --- | --- | --- |
| ***Puccinia striiformis tritici (Pst)*** | | | |
| 1 | YR_110S84 | *Yr1, Yr4, Yr5, Yr10, Yr13, Yr14, Yr15, Yr16, Yr24, Yr26, Yrsk, YrA* | *Yr2, Yr6, Yr7, Yr8, Yr9, Yr11, Yr12, Yr17, Yr18, Yr19, Yr21, Yr22, Yr23, Yr25, Yr27, Yrso, Yrsd* |
| 2 | YR_110S119 | *Yr1, Yr5, Yr10, Yr13, Yr14, Yr15, Yr16, Yr24, Yr26, Yrsp, Yrsk* | *Yr2, Yr3, Yr4, Yr6, Yr7, Yr8, Yr9, Yr11, Yr12, Yr17, Yr18, Yr19, Yr21, Yr22, Yr23, Yr25, YrA, Yrso* |
| 3 | YR_238S119 | *Yr1, Yr4, Yr5, Yr10, Yr13, Yr14, Yr15, Yr16, Yrsk, YrA* | *Yr2, Yr3, Yr6, Yr7, Yr8, Yr9, Yr11, Yr12, Yr17, Yr18, Yr19, Yr21, Yr22, Yr23, Yr24, Yr25, Yr26, Yr27, Yrri, Yrso, Yrsd* |
| 4 | YR_T (47S103) | *Yr1, Yr4, Yr5, Yr9,Yr10, Yr11, Yr12, Yr13, Yr14, Yr15, Yr16, Yr24, Yr26, Yrsp, Yrso , Yrsk* | *Yr2, Yr3Yr6, Yr7, Yr8, Yr17, Yr18, Yr19, Yr21,*  *Yr22, Yr23, Yr25, YrA, Yrsd, Yrso* |
| 5 | YR_46S119 | *Yr1, Yr5, Yr10, Yr11, Yr12, Yr13, Yr14, Yr15, Yr16, Yr24, Yr26, Yrsp, Yrso, Yrsk* | *Yr2, Yr3, Yr4, Yr6, Yr7, Yr8, Yr9, Yr17, Yr18, Yr19, Yr21, Yr22, Yr23, Yr25, YrA, Yrsd, Yrso* |
| ***Puccinia triticina (Pt)*** | | | |
| 1 | LR_12-5 | *Lr1, Lr2a, Lr9, Lr10, Lr13*, Lr15, Lr19, Lr24, Lr25, Lr28, Lr29, Lr32, Lr36*, Lr39, Lr42, Lr43, Lr45* | *Lr2b, Lr2c, Lr3, Lr11, Lr12, Lr14a, Lr14b, Lr14ab, Lr16, Lr17a*, Lr17b, Lr18, Lr20, Lr21, Lr22a, Lr22b, Lr23, Lr26, Lr27+31, Lr30, Lr33, Lr34, Lr35, Lr37, Lr38, Lr40, Lr44, Lr46, Lr48, Lr49* |
| 2 | LR_77-1 | *Lr9, Lr17a, Lr17b, Lr19, Lr23, Lr24, Lr25, Lr27+31, Lr28, Lr29, Lr32, Lr36, Lr39, Lr42, Lr43, Lr45, Lr47* | *Lr1, Lr2a, Lr2b, Lr2c, Lr3, Lr10, Lr11, Lr12, Lr13, Lr14a, Lr14b, Lr14ab, Lr15, Lr16, Lr18, Lr20, Lr21, Lr22a, Lr22b, Lr26, Lr30, Lr33, Lr35, Lr37, Lr38, Lr44, Lr48, Lr49* |
| 3 | LR_77-5 | *Lr9, Lr18*, Lr19, Lr24, Lr25, Lr28, Lr29, Lr32, Lr40, Lr41, Lr42, Lr45* | *Lr1, Lr2a, Lr2b, Lr2c, Lr3, Lr10, Lr11, Lr12, Lr13, Lr14a, Lr14b, Lr14ab, Lr15, Lr16, Lr17a, Lr20, Lr21, Lr22a, Lr22b, Lr23, Lr26, Lr27+31, Lr30, Lr33, Lr34, Lr35, Lr36, Lr37, Lr38, Lr43, Lr44, Lr48, Lr49* |
| 4 | LR_77-9 | *Lr2a, Lr2b, Lr2c, Lr9, Lr19, Lr24, Lr25, Lr28, Lr32, Lr39, Lr42, Lr45, Lr47* | *Lr1, Lr3, Lr10, Lr11, Lr12, Lr13, Lr14a, Lr14b, Lr14ab, Lr15, Lr16, Lr17a, Lr17b, Lr18, Lr20, Lr21, Lr22a, Lr22b, Lr23, Lr26, Lr27+3, Lr30, Lr33, Lr34, Lr35, Lr36, Lr37, Lr38, Lr44, Lr46, Lr48, Lr49* |
| 5 | LR_104-2 | *Lr9, Lr10*, Lr13*, Lr15, Lr19, Lr20, Lr24, Lr25, Lr28, Lr29, Lr32, Lr36, Lr40, Lr41, Lr42, Lr43, Lr45* | *Lr1, Lr2a*, Lr2b, Lr2c, Lr3, Lr11, Lr12, Lr14a, Lr14b, Lr14ab, Lr16, Lr17a, Lr18, Lr21, Lr22a, Lr22b, Lr23, Lr26, Lr27+31, Lr30, Lr33, Lr34, Lr35, Lr37, Lr38, Lr44, Lr48, Lr49* |
| 6 | LR_106 | *Lr1, Lr2a, Lr2b, Lr3, Lr9, Lr10, Lr11, Lr12, Lr13, Lr14a, Lr14b, Lr14ab, Lr15, Lr16, Lr17, Lr18, Lr19, Lr21, Lr22a, Lr22b, Lr23, Lr24, Lr25, Lr26, Lr27+31, Lr30, Lr33, Lr37, Lr38, Lr44, Lr48* | *Lr2c, Lr20, Lr35* |
| ***Puccinia graminis tritici (Pgt)*** | | | |
| 1 | SR_11 | *Sr7a, Sr8a, Sr8b, Sr9e, Sr22, Sr23, Sr24, Sr25, Sr26, Sr27, Sr31, Sr32, Sr33, Sr35, Sr37, Sr39, Sr40, Sr43, SrTmp, SrTt3* | *Sr2, Sr5, Sr6, Sr7b, Sr9a, Sr9b, Sr9c, Sr9d, Sr9f, Sr9g, Sr10, Sr11, Sr13, Sr14, Sr15, Sr16, Sr17, Sr18, Sr19, Sr20, Sr21, Sr28, Sr29, Sr30, Sr34, Sr36, Sr38, SrMcN* |
| 2 | SR_21A-2 | *Sr5, Sr6, Sr7a, Sr8a, Sr8b, Sr9a, Sr9b, Sr9c, Sr9e, Sr11, Sr12, Sr21, Sr22, Sr23, Sr24, Sr25, Sr26, Sr27, Sr29, Sr30, Sr31, Sr32, Sr33, Sr35, Sr37, Sr38, Sr39, Sr40, Sr43, SrGt, SrTmp, SrTt3* | *Sr2, Sr7b, Sr9d, Sr9f, Sr9g, Sr10, Sr13, Sr14, Sr15, Sr16, Sr17, Sr19, Sr28, Sr34, Sr36, SrMcN* |
| 3 | SR_34-1 | *Sr6, Sr7a, Sr8a, Sr8b, Sr9a, Sr9e, Sr10, Sr11, Sr13, Sr17, Sr19, Sr21, Sr22, Sr23, Sr24, Sr25, Sr26, Sr27, Sr30, Sr31, Sr32, Sr33, Sr35, Sr36, Sr37, Sr39, Sr40, Sr43, SrTmp, SrTt3* | *Sr2, Sr5, Sr7b, Sr9b, Sr9d, Sr9f, Sr9g, Sr14, Sr15, Sr16, Sr18, Sr20, Sr21, Sr28, Sr29, Sr34, Sr38, SrMcN* |
| 4 | SR_40A | *Sr7a, Sr13, Sr21, Sr22, Sr24, Sr25, Sr26, Sr27, Sr30, Sr31, Sr32, Sr33, Sr35, Sr36, Sr37, Sr39, Sr40, Sr43, SrTmp, SrTt3* | *Sr2, Sr5, Sr6, Sr7b, Sr8a, Sr8b, Sr9a, Sr9b, Sr9d, Sr9e, Sr9f, Sr9g, Sr10, Sr14, Sr15, Sr16, Sr17, Sr18, Sr19, Sr20, Sr23, Sr28, Sr29, Sr34, SrMcN* |
| 5 | SR_40-3 | *Sr21, Sr22, Sr24, Sr25, Sr26, Sr27, Sr31, Sr32, Sr33, Sr35, Sr36, Sr37, Sr39, Sr40, Sr42, Sr43, SrTmp, SrTt3* | *Sr2, Sr5, Sr6, Sr7a, Sr7b, Sr8a, Sr8b, Sr9a, Sr9b, Sr9d, Sr9e, Sr9f, Sr9g, Sr10, Sr11, Sr14, Sr15, Sr16, Sr17, Sr18, Sr19, Sr20, Sr23, Sr28, Sr29, Sr30, Sr34, Sr38, Sr44, SrMcN, SrGt* |
| 6 | SR_117-6 | *Sr5, Sr8a, Sr8b, Sr9b, Sr22, Sr24, Sr25, Sr26, Sr27, Sr28, Sr30, Sr31, Sr32, Sr33, Sr35, Sr36, Sr37, SrTmp* | *Sr2, Sr6, Sr7a, Sr7b, Sr9e, Sr9f, Sr9g, Sr10, Sr11, Sr12, Sr13, Sr14, Sr15, Sr16, Sr17, Sr19, Sr21, Sr23, Sr29, Sr34, SrMcN* |
| 7 | SR_122 | *Sr7a, Sr8a, Sr8b, Sr9e, Sr10, Sr12, Sr14, Sr15, Sr16, Sr17, Sr18, Sr19, Sr20, Sr22, Sr24, Sr25, Sr26, Sr27, Sr28, Sr31, Sr32, Sr33, Sr35, Sr36, Sr37, Sr38, Sr39, Sr40, Sr43, SrTmp, SrTt3* | *Sr2, Sr5, Sr6, Sr7bSr9a, Sr9b, Sr9c, Sr9d, Sr9f, Sr9g, Sr11, Sr13, Sr21, Sr23, Sr29, Sr30, Sr34, SrMcN* |
