# Supplementary material for: Genome-Wide Association Studies in Diverse Spring Wheat Panel for Stripe, Stem, and Leaf Rust Resistance
Source: Front Plant Sci. 2020 Jun 3;11:748. doi: 10.3389/fpls.2020.00748 (PMC7286347; doi:10.3389/fpls.2020.00748)
Supplement: TABLE S2 — Nomenclature of environments (12) considered for the evaluation of field disease response at adult plant stage. [file Table_2.DOCX]

Supplementary Table S2: Nomenclature of environments (12) considered for the evaluation of field disease response at adult plant stage.

| **Year** | **Stripe rust (YR)** | | **Leaf rust (LR)** | | **Stem rust (SR)** | |
| --- | --- | --- | --- | --- | --- | --- |
| **2017-18** | **Location** | **Code** | **Location** | **Code** | **Location** | **Code** |
|  | Karnal | YR_E1 | Indore | LR_E1 | Indore | SR_E1 |
|  | Uchani | YR_E2 | Wellington | LR_E2 | Wellington | SR_E2 |
| **2018-19** | Karnal | YR_E3 | Indore | LR_E3 | Indore | SR_E3 |
|  | Uchani | YR_E4 | Wellington | LR_E4 | Wellington | SR_E4 |
