# Supplementary material for: Genome-Wide Association Studies in Diverse Spring Wheat Panel for Stripe, Stem, and Leaf Rust Resistance
Source: Front Plant Sci. 2020 Jun 3;11:748. doi: 10.3389/fpls.2020.00748 (PMC7286347; doi:10.3389/fpls.2020.00748)
Supplement: TABLE S7 — Phenotypic reactions of the 483 genotypes included in the study to multiple rusts at seedling and adult stage, grouped based on population structure. [file Table_7.DOCX]

Supplementary Table S7: Phenotypic reactions of the 483 genotypes included in the study to multiple rusts at seedling and adult stage, grouped based on population structure.

|  | Stripe rust pathotypes (seedling stage) | | | | | | |
| --- | --- | --- | --- | --- | --- | --- | --- |
| Subpopulation | YR_110S119 | YR_T | YR_238S119 | YR_46S119 | YR_110S84 |  |  |
| SP1 | **7.651** | 6.442 | **5.710** | **3.871** | 6.453 |  |  |
| SP2 | **7.538** | 4.742 | **5.622** | **3.414** | 4.925 |  |  |
|  | Leaf rust pathotypes (seedling stage) | | | | | | |
|  | LR_106 | LR_77-5 | LR_104-2 | LR_77-9 | LR_12-5 | LR_77-1 |  |
| SP1 | 5.752 | 7.520 | 7.374 | 7.604 | 7.128 | 6.760 |  |
| SP2 | 0.777 | 6.437 | 5.742 | 6.577 | 4.392 | 3.748 |  |
|  | Stem rust pathotypes (seedling stage) | | | | | | |
|  | SR_40A | SR_21A2 | SR_11 | SR_34-1 | SR_40-3 | SR_117-6 | SR_122 |
| SP1 | 7.406 | 4.936 | 6.723 | 5.159 | 5.131 | 6.943 | 5.779 |
| SP2 | 4.705 | 1.741 | 3.503 | 0.611 | 1.999 | 1.992 | 1.736 |
|  | Stripe rust (adult stage) | | | | | | |
|  | COI_YR_E1 | COI_YR_E2 | COI_YR_E3 | COI_YR_E4 |  |  |  |
| SP1 | 42.019 | 49.981 | 45.264 | 65.019 |  |  |  |
| SP2 | 28.072 | 35.037 | 26.005 | 38.902 |  |  |  |
|  | Leaf rust (adult stage) | | | | | | |
|  | COI_LR_E1 | COI_LR_E2 | COI_LR_E3 | COI_LR_E4 |  |  |  |
| SP1 | 42.245 | 39.695 | 31.375 | 29.179 |  |  |  |
| SP2 | 12.618 | 16.780 | 13.261 | 12.623 |  |  |  |
|  | Stem rust (adult stage) | | | | | | |
|  | COI_SR_E1 | COI_SR_E2 | COI_SR_E3 | COI_SR_E4 |  |  |  |
| SP1 | 39.152 | **23.274** | 39.587 | 26.210 |  |  |  |
| SP2 | 19.840 | **20.112** | 23.469 | 19.546 |  |  |  |

Across individual subpopulations, averaged score of phenotypic data was considered (Supplementary Table S3). Non-significant (*P* > 0.05) differences of mean IT and COI between subpopulations are shown in bold font.
